# Organelle Crosstalk Regulators Are Regulated in Diseases, Tumors, and Regulatory T Cells: Novel Classification of Organelle Crosstalk Regulators

**DOI:** 10.3389/fcvm.2021.713170

**Published:** 2021-07-22

**Authors:** Ming Liu, Na Wu, Keman Xu, Fatma Saaoud, Eleni Vasilopoulos, Ying Shao, Ruijing Zhang, Jirong Wang, Haitao Shen, William Y. Yang, Yifan Lu, Yu Sun, Charles Drummer, Lu Liu, Li Li, Wenhui Hu, Jun Yu, Domenico Praticò, Jianxin Sun, Xiaohua Jiang, Hong Wang, Xiaofeng Yang

**Affiliations:** ^1^Centers for Cardiovascular Research, Lewis Katz School of Medicine at Temple University, Philadelphia, PA, United States; ^2^Department of Cell Biology and Genetics, School of Basic Medical Science, Shanxi Medical University, Taiyuan, China; ^3^Departments of Endocrinology and Emergency Medicine, Shengjing Hospital of China Medical University, Shenyang, China; ^4^Department of Nephrology, The Affiliated People's Hospital of Shanxi Medical University, Taiyuan, China; ^5^Department of Cardiology, The Affiliated People's Hospital of Shanxi Medical University, Taiyuan, China; ^6^Rutgers University, New Brunswick, NJ, United States; ^7^Metabolic Disease Research, Inflammation, Translational & Clinical Lung Research, Thrombosis Research, Lewis Katz School of Medicine at Temple University, Philadelphia, PA, United States; ^8^Alzheimer's Center, Lewis Katz School of Medicine at Temple University, Philadelphia, PA, United States; ^9^Department of Medicine, Center for Translational Medicine, Thomas Jefferson University, Philadelphia, PA, United States

**Keywords:** organelle crosstalk, inflammation, cancers and tumors, viral infections, endothelial cell activation, Treg

## Abstract

To examine whether the expressions of 260 organelle crosstalk regulators (OCRGs) in 16 functional groups are modulated in 23 diseases and 28 tumors, we performed extensive -omics data mining analyses and made a set of significant findings: (1) the ratios of upregulated vs. downregulated OCRGs are 1:2.8 in acute inflammations, 1:1 in metabolic diseases, 1:1.2 in autoimmune diseases, and 1:3.8 in organ failures; (2) sepsis and trauma-upregulated OCRG groups such as vesicle, mitochondrial (MT) fission, and mitophagy but not others, are termed as the cell crisis-handling OCRGs. Similarly, sepsis and trauma plus organ failures upregulated seven OCRG groups including vesicle, MT fission, mitophagy, sarcoplasmic reticulum–MT, MT fusion, autophagosome–lysosome fusion, and autophagosome/endosome–lysosome fusion, classified as the cell failure-handling OCRGs; (3) suppression of autophagosome–lysosome fusion in endothelial and epithelial cells is required for viral replications, which classify this decreased group as the viral replication-suppressed OCRGs; (4) pro-atherogenic damage-associated molecular patterns (DAMPs) such as oxidized low-density lipoprotein (oxLDL), lipopolysaccharide (LPS), oxidized-1-palmitoyl-2-arachidonoyl-sn-glycero-3-phosphocholine (oxPAPC), and interferons (IFNs) totally upregulated 33 OCRGs in endothelial cells (ECs) including vesicle, MT fission, mitophagy, MT fusion, endoplasmic reticulum (ER)–MT contact, ER– plasma membrane (PM) junction, autophagosome/endosome–lysosome fusion, sarcoplasmic reticulum–MT, autophagosome–endosome/lysosome fusion, and ER–Golgi complex (GC) interaction as the 10 EC-activation/inflammation-promoting OCRG groups; (5) the expression of OCRGs is upregulated more than downregulated in regulatory T cells (Tregs) from the lymph nodes, spleen, peripheral blood, intestine, and brown adipose tissue in comparison with that of CD4^+^CD25^−^ T effector controls; (6) toll-like receptors (TLRs), reactive oxygen species (ROS) regulator nuclear factor erythroid 2-related factor 2 (Nrf2), and inflammasome-activated regulator caspase-1 regulated the expressions of OCRGs in diseases, virus-infected cells, and pro-atherogenic DAMP-treated ECs; (7) OCRG expressions are significantly modulated in all the 28 cancer datasets, and the upregulated OCRGs are correlated with tumor immune infiltrates in some tumors; (8) tumor promoter factor IKK2 and tumor suppressor Tp53 significantly modulate the expressions of OCRGs. Our findings provide novel insights on the roles of upregulated OCRGs in the pathogenesis of inflammatory diseases and cancers, and novel pathways for the future therapeutic interventions for inflammations, sepsis, trauma, organ failures, autoimmune diseases, metabolic cardiovascular diseases (CVDs), and cancers.

## Introduction

Cardiovascular diseases (CVDs), such as coronary heart disease, hypertension, stroke, and peripheral artery disease, have become the number 1 cause of death globally (1, 2). We and others recently reported that CVD stressors and risk factors such as hyperlipidemia (3, 4), hyperglycemia (5), hyperhomocysteinemia (6, 7), and chronic kidney disease (8–10) promote atherosclerosis and vascular inflammation via several mechanisms. These mechanisms include innate immune activation (11) of endothelial cells (ECs) (3, 12–15) promoting EC injury (16); Ly6Chigh inflammatory mouse monocyte and CD40^+^ human monocyte differentiation (7, 17–19); disease reprogrammed macrophages (20–22); cytokine and secretome regulation (23–30); decreased/transdifferentiated CD4^+^Foxp3^+^ regulatory T cells (Treg) (24, 31–34); and impaired vascular repairability of bone marrow-derived progenitor cells (35, 36). In addition, we recently proposed new models such as intracellular organelle dangers (37) and reactive oxygen species (ROS) as an integrated sensing system for metabolic homeostasis and alarming (38), which indicated that metabolic reprogramming and dysfunction trigger mitochondrial (MT) ROS (4, 39–42); caspase-1/inflammasome activation (8, 10) downregulated histone modification enzymes (43) and increased expressions of trained immunity pathway enzymes (22, 39, 44–47). These reports have clearly demonstrated that mitochondria play significant roles in connecting metabolic reprogramming and dysfunction to inflammation initiation and gene transcription. However, the question of how organelle crosstalk regulates the progression of various diseases remains poorly characterized.

With progress of super-multiplexed optical imaging and barcoding (48) and other system-level spectral imaging and analyses (49, 50), it has been recognized that the crosstalk between mitochondria and other organelles, such as lysosomes, peroxisomes, and the endoplasmic reticulum (ER) (51), is mediated by transcriptional programs and other signaling mechanisms (52). Organelle exchange materials including lipids, ions, and proteins at the membrane contact sites (MCSs), which may be different from membrane trafficking between organelles by vesiculotubular carriers regulated by Rab GTPase (53). Organelle interactions also likely mediate organelle dysfunction downstream of MT impairments (54). In addition, external or internal stress activates several well-orchestrated processes aimed at either restoring cellular homeostasis or committing cell death (55). These processes include unfolded protein response (UPR) in ER and mitochondria (56), autophagy, hypoxia, and MT function, which underlie the ER stress response (57), suggesting that cellular pathological responses may be mediated in organelle crosstalk mechanisms (58). Accordingly, alterations to these networks, such as impaired ER–mitochondria MCSs, have been linked to several diseases such as neurodegeneration (59, 60), CVD (61), diabetes (62), kidney diseases (63, 64), and cancers (65, 66).

Regardless of significant progress in the field, the following questions remained poorly understood: (1) how many regulators (regulatomes) participate in organelle crosstalk; (2) whether the expressions of organelle crosstalk regulatomes are modulated in various disease conditions such as acute inflammation (AI) and injuries, chronic inflammations, autoimmune diseases (ADs), metabolic diseases (MDs), and cancers; (3) whether the expressions of organelle crosstalk regulatomes are differentially modulated in various cell types in response to pathological stimuli; and (4) what are the potential mechanisms regulating expression of organelle crosstalk regulators (OCRGs). To address these important questions, we examined our novel hypothesis that pathological conditions and various DAMPs significantly modulate the expressions of organelle crosstalk regulators in disease-specific and cell type-specific manner. To test this hypothesis, we collected 260 organelle crosstalk regulators as the regulatomes that participate in 16 organelle crosstalk processes including (1) MT biogenesis, (2) MT fission, (3) MT fusion, (4) MT fission and fusion, (5) mitophagy, (6) MT protein translocation, (7) MT contact site, (8) ER (67)–MT contact (68), (9) sarcoplasmic reticulum–MT (69), (10) ER–plasma conjunctions (70, 71), (11) ER–Golgi interaction, (12) ER–endosome, (13) autophagosome–lysosome fusion, (14) autophagosome–endosome/lysosome fusion (68), (15) endosome–Golgi trafficking, and (16) vesicle (Table 1). We performed an extensive -omics database mining and determined the expressions of 260 organelle crosstalk regulators in more than 50 microarrays from more than 20 diseases and 28 cancers/tumors. Corresponding mechanisms were explored, such as the regulation of (a) (TLRs); (b) caspase-1; (c) ROS; and (d) oncogenes/tumor suppressors and made a set of significant findings. Our findings have provided novel insights on pathophysiological roles of OCRGs in various inflammations, diseases, and cancers and provide novel therapeutic targets and strategies for various inflammations, CVD, MDs, and cancers.

**Table 1:** Two hundred sixty organelle crosstalk regulators (OCRGs) were searched from three databases such as National Center for Biotechnology Information (NCBI), Gene Set Enrichment Analysis (GSEA), and the Human Protein Atlas (HPA) website.

**Classification of OCRGs**	**No**.	**Gene symbol**	**Source**
Mitochondrial biogenesis	5	POLG, VCP, HSPD1, HSPA4, LONP1	PMID: 28094012, 24784582, 22298039, 22424226, 21659532
Mitochondrial fission	28	COX10, DCN, DDHD1, DDHD2, DHODH, DNM1, DNM2, DNM3, GGNBP1, INF2, KDR, LRRK2, MAPT, MIEF1, MIEF2, MTFP1, MTFR1, MTFR1L, MTFR2, MX1, MX2, MYO19, PINK1, PPARGC1A, SLC25A46, STAT2, TMEM135, VPS35	Gene Set Enrichment Analysis (GSEA) (https://www.gsea-msigdb.org/gsea/index.jsp)
Mitochondrial fusion	15	ADCK1, AFG3L2, BAK1, BAX, CHCHD3, MFN1, MFN2, MIGA1, MIGA2, OMA1, PID1, PLD6, STOML2, USP30,VAT1	GSEA (https://www.gsea-msigdb.org/gsea/index.jsp)
Mitochondrial fission and fusion	7	BNIP3, DNM1L, FIS1, GDAP1, MFF, MUL1, OPA1	GSEA (https://www.gsea-msigdb.org/gsea/index.jsp)
Mitophagy	28	PINK1, OPTN, SQSTM1, PARL, USP30, RING1, RNF2, ATG9a, VMP1, CALCOCO2, ATG4a,ATG7, ATG3, ATG12, ATG5, ATG10, ATG13, OMA1, TBK1, GABARAP, MAP1LC3A, VDAC1, BNIP3L, BNIP3, FUNDC1, BCL2L13, FKBP8,	PMID: 27291334, 25479550, 29358684, 29370689, 30540226
Mitochondrial protein translocation	7	TOMM5, TOMM6, TOMM7, TOMM20, TOMM22, TOMM40, TOMM70	PMID: 28301740
Mitochondrial contact site	4	CHCHD3, CHCHD6, APOO, APOOL, IMMT	PMID: 24687277
Endoplasmic reticulum–mitochondria contact	13	RMDN3, VAPB, VAPA, BCAP31, MCU, ITPR1, HSPA9, BAK1, DAPK1, MFN1, MFN2, VDAC1, FIS1	PMID: 28301744, 26627931
Sarcoplasmic reticulum–mitochondria contact	9	VDAC1, HSPA9, ITPR1, SIGMAR1, ATP2A1, VAPB, RMDN3, OSBPL8, OSBPL5	PMID: 28275246
Endoplasmic reticulum–plasma membrane junctions	10	ORAI1, STIM1, SEC61B, JPH1, JPH2, JPH3, JPH4, PTPN1, STX1A, SEC22B	PMID: 26322679, 22914293, 28554772, 28301744
Endoplasmic reticulum–Golgi interaction	6	VAPA, VAPB, PLEKHA8, PITPNM1, OSBP, STX5	PMID: 32065234, 23913272, 24209621, 31357511
Endoplasmic reticulum–endosome	7	STARD3, PTPN1, EGFR, OSBPL1A, STARD3NL, RAB7A, NPC1	PMID: 26627931
Autophagosome–lysosome fusion	4	OCRL, MCOLN1, TLR9, TIRAP	PMID: 27398910
Autophagosome–endosome/lysosome fusion	9	VAMP8, RAB7A, PLEKHM1, RILP, ATG14, VPS39, VPS41, SNAP29, STX17	PMID: 27283760
Endosome–Golgi trafficking	4	TBC1D23, GOLGA1, GOLGA4, FAM91A1	PMID: 29084197
Vesicle	125	AASS, ABCD3, ABHD14A, ACAA1, ACBD5, ADCYAP1R1, AGPS, AKAP9, ANKFY1, ANKRD2, ANKRD6, AP1B1, AP1G1, ARCN1, ATP11A, ATP23, AZU1, BMP2, C7orf43, CAT, CCDC93, CCZ1, CCZ1B, CD24, CDKL1, CHGB, CHIC2, CLIP4, CLOCK, CLTA, CLTC, CNNM2, CSF2, CSTF2T, CTAG2, CTSA, CYB5A, CYP27C1, DAB1, DAB, DBH, DNAJA3, DPP7, DRAM2, DYRK4, ECI2, EDA, EIF4ENIF1, EPS15, ERGIC1, FAF2, FYCO1, GPRC5A, GRN, GTPBP2, HDHD3, HEXIM1, HGS, HIP1, HSD17B4, HSD3B7, IGF2R, ITCH, ITM2B, KLRG1, LAMP3, MBD1, MEF2D, MIA3, MITD1, NACC1, NMRK2, NOV, NSDHL, OSBPL5, P4HA2, PDCD6IP, PDXDC1, PEX14, PICALM, PLCH1, PLIN3, PLIN4, PNPLA2, POU2F2, PPARG, PPOX, PUSL1, RAB11A, RAB11FIP1, RAB11FIP5, RAB20, RAB30, RAB5C, RALY, RANBP2, RANGAP1, RC3H2, RPS6KC1, RPTOR, RUNX1, SAMD9, SEC23IP, SERPINA1, SERTM1, SET, SNX1, SPINK5, SQSTM1, STK11IP, STX16, SYNDIG1, SYNPO2, TBK1, TMEM63A, TNPO3, TYR, VAC14, VPS26A, VTI1B, ZDHHC13, ZFYVE16, ZFYVE9, ZNF266, ZNF326	The human protein atlas (HPA) (https://www.proteinatlas.org/humanproteome/cell/vesicles)

## Methods

### Expression Profile of Organelle Crosstalk Regulator Genes in Microarray Data From Patients With Various Inflammatory Diseases and Cancers

The 25 microarray datasets of AI and injuries, ADs, MDs, and CVDs (Table 2); one microarray dataset of Middle East respiratory syndrome coronavirus (MERS-CoV)-infected human microvascular ECs; one microarray dataset of severe respiratory syndrome coronavirus (SARS-CoV)-infected human airway epithelial cells; two microarray datasets of influenza virus-infected lung epithelial cells, Calu-3 cells (non-small-cell lung cancer cell line), and human umbilical vein ECs (HUVECs); one microarray of Kaposi sarcoma-associated herpes virus-infected primary human dermal ECs (Table 3); six microarrays of ECs treated by pro-atherogenic DAMPs (Table 4); six microarray datasets of Treg cells (Table 5); 15 microarrays of Treg regulator deficiency (Table 6); six microarrays of TLR deficiencies (Table 7); one microarray of ROS negative regulator nuclear factor erythroid 2-related factor 2 (Nrf2) deficiency (Table 8); and two microarrays of caspase-1 deficiency (Table 9) were collected from National Institutes of Health (NIH)–National Center for Biotechnology Information (NCBI)–Gene Expression Omnibus (GEO) databases (https://www.ncbi.nlm.nih.gov/gds/) and analyzed with an online software GEO2R (https://www.ncbi.nlm.nih.gov/geo/geo2r/). In addition, gene expression data from 28 cancers were analyzed with the Gene Expression Profiling Interactive Analysis (GEPIA2) database (http://gepia2.cancer-pku.cn/#index), in which dataset sources were from The Cancer Genome Atlas (TCGA)/Genotype-Tissue Expression (GTEx) data (Table 10). Furthermore, 19 microarray datasets of oncogene and tumor suppressor deficiencies were collected from NCBI–GEO and analyzed with GEO2R (Table 11). The differentially expressed OCRGs and their changes in all microarrays and TCGA datasets are listed in Supplementary Tables 1, 2. The original microarray experiments used different cells, which prevented us from comparing the effects of disease conditions in regulating OCRGs in the same cell types. Of note, our approach was well-justified. For example, as a common practice, we (23) and others (72) often studied gene expression in non-ideal heterogeneous peripheral blood mononuclear cell (PBMC) populations in pathophysiological conditions, which are actually composed of many cell types (also see the Discussion section).

**Table 2:** Twenty-five microarray datasets including acute inflammations, metabolic diseases, autoimmune diseases, and organ failure diseases in the National Institutes of Health (NIH)–National Center for Biotechnology Information (NCBI)–Gene Expression Omnibus (GEO) dataset database (https://www.ncbi.nlm.nih.gov/gds/) were collected to analyze the expression changes of organelle crosstalk regulators (OCRGs).

**GEO ID**	**Disease/phenotype**	**Tissue**	**Comparison**	**Upregulated**	**Downregulated**	**PMID**
**Acute inflammation**						
GSE32707	Lung injury	Whole blood	Sepsis day 0 vs. without sepsis	4	8	22461369
	Lung injury	Whole blood	Sepsis day 7 vs. without sepsis	6	2	22461369
	Lung injury	Whole blood	Sepsis ARDS day 0 vs. without sepsis ARDS	2	10	22461369
	Lung injury	Whole blood	Sepsis ARDS day 7 vs. without Sepsis ARDS	7	5	22461369
GSE13904	Septic shock	Whole blood	Sepsis day 1 vs. normal	13	6	19325468
	Septic shock	Whole blood	Sepsis day 3 vs. normal	3	1	19325468
	Septic shock	Whole blood	Sepsis shock day 1 vs. normal	9	9	19325468
	Septic shock	Whole blood	Sepsis shock day 3 vs. normal	12	13	19325468
GSE5580	Severe trauma	Monocytes	Severe trauma vs. health	4	20	17032758
	Severe trauma	Leukocytes	Severe trauma vs. health	3	21	17032758
	Severe trauma	T cells	Severe trauma vs. health	8	23	17032758
**Metabolic disease**						
GSE2508	Obese	Adipocytes	Non-diabetic obese vs. non-diabetic lean	1	2	16059715
GSE48964	Obese	Adipose stem cells	Morbidly obese vs. nonobese	0	1	24040759
GSE55200	MHO	Subcutaneous adipose	MHO vs. LH	0	1	24933025
	MUO	Subcutaneous adipose	MUO vs. LH	1	1	24933025
GSE94752	Obese IR	Adipocytes	Obese IR vs. lean	2	0	28570579
	Obese IS	Adipocytes	Obese IS vs. lean	0	0	28570579
GSE23343	T2D	Liver	T2D vs. normal glucose tolerance	7	7	21035759
GSE29221	T2D	Skeletal muscle	T2D vs. non-diabetes	10	22	23308243
GSE29226	T2D	Subcutaneous adipose	T2D vs. non-diabetes	6	6	23308243
GSE29231	T2D	Visceral adipose	T2D vs. non-diabetes	17	5	23308243
GSE28829	Atherosclerosis	Carotid artery	Advanced plaque vs. early plaque	3	0	22388324
GSE41571	Atherosclerosis	Macrophages from plaques	Ruptured plaques vs. stable plaque	14	11	23122912
GSE6054	FHC and atherosclerosis	Monocytes	FHC homozygote vs. control	5	9	19040724
GSE6088	FHC and atherosclerosis	T cell	FHC homozygote vs. control	4	7	19040724
GSE1010	FCH	Lymphoblastic cells	FCH vs. normal	1	2	15388524
**Autoimmune disease**						
GSE10500	RA	Macrophages*	RA vs. normal	18	31	18345002
GSE97779	RA	Macrophages*	RA vs. normal	25	40	28813657
GSE109248	ACLE	Skin	ACL vs. normal	15	9	29889098
	CCLE	Skin	CCL vs. normal	15	5	29889098
	Psoriasis	Skin	Psoriasis vs. normal	39	20	29889098
	SCLE	Skin	SCL vs. normal	22	24	29889098
GSE38713	UC	Sigmoid colon or rectum	UC active involved vs. normal	13	13	23135761
GSE3365	UC	PBMC	UC vs. normal	1	4	16436634
	CD	PBMC	CD vs. normal	2	1	16436634
**Organ failure**						
GSE76701	Heart failure	Left ventricle	Failing heart vs. non-failing heart	1	2	26756417
GSE38941	HBV-ALF	Liver	HBV-ALF vs. normal	24	25	23185381
GSE37171	ESRF	Whole blood	Chronic renal failure vs. healthy controls	0	38	23809614
GSE15072	CKD hemodialysis	PBMC	Hemodialysis vs. healthy controls	16	63	19698090

*Expression of OCRGs was examined in a total of 39 comparison datasets. There are significantly expressed OCRGs in 38 datasets (except GSE94752 obese IS vs. lean) (p < 0.05, |logFC| > 1)*.

Macrophages*, macrophages from synovial fluids for RA and blood-derived macrophages for control. All the studies and samples are from humans.

*ARDS, acute respiratory distress syndrome; ALI, acute liver injury; ALF, acute liver failure; PBMC, peripheral blood mononuclear cell; IS, insulin sensitive; IR, insulin resistance; MHO, metabolically healthy obese; MUO, metabolically unhealthy obese; LH, lean health; T2D, type 2 diabetes; FCH, familial combined hyperlipidemia; FHC, familial hypercholesterolemia; RA, rheumatoid arthritis; ACLE, acute cutaneous lupus; CCLE, chronic cutaneous lupus; SCLE, subacute cutaneous lupus; UC, ulcerative colitis; CD, Crohn's Disease; HBV-ALF, hepatitis B virus-associated acute liver failure; ESRF, end-stage renal failure; CKD, chronic kidney disease*.

**Table 3:** Three microarray datasets of time course including MERS coronavirus, SARS coronavirus, avian influenza virus, one dataset of influenza virus infected human umbilical vein endothelial cells and one dataset of Kaposi sarcoma-associated herpes virus infection in the National Institutes of Health (NIH)–National Center for Biotechnology Information (NCBI)–Gene Expression Omnibus (GEO) datasets database (https://www.ncbi.nlm.nih.gov/gds/) were collected to analyze the expression changes of genes that we are interested in.

**GEO ID**	**Comparison**	**Cell/tissue**	**Upregulated**	**Downregulated**	**PMID**
GSE79218	icMERS-inoculated vs. mock-inoculated (0 h)	Human microvascular endothelial cells	0	1	28830941
	icMERS-inoculated vs. mock-inoculated (12 h)	Human microvascular endothelial cells	20	16	28830941
	icMERS-inoculated vs. mock-inoculated (24 h)	Human microvascular endothelial cells	41	44	28830941
	icMERS-inoculated vs. mock-inoculated (36 h)	Human microvascular endothelial cells	39	46	28830941
	icMERS-inoculated vs. mock-inoculated (48 h)	Human microvascular endothelial cells	25	24	28830941
GSE47960	SARS-CoV-infected vs. mock-infected (0 h)	Human airway epithelium cells	3	0	23935999
	SARS-CoV-infected vs. mock-infected (12 h)	Human airway epithelium cells	0	0	23935999
	SARS-CoV-infected vs. mock-infected (24 h)	Human airway epithelium cells	2	1	23935999
	SARS-CoV-infected vs. mock-infected (36 h)	Human airway epithelium cells	1	0	23935999
	SARS-CoV-infected vs. mock-infected (48 h)	Human airway epithelium cells	3	0	23935999
	SARS-CoV-infected vs. mock-infected (60 h)	Human airway epithelium cells	15	0	23935999
	SARS-CoV-infected vs. mock-infected (72 h)	Human airway epithelium cells	4	2	23935999
	SARS-CoV-infected vs. mock-infected (84 h)	Human airway epithelium cells	5	0	23935999
	SARS-CoV-infected vs. mock-infected (96 h)	Human airway epithelium cells	7	1	23935999
	H1N1-infected vs. mock-infected (0 h)	Human airway epithelium cells	1	0	23935999
	H1N1-infected vs. mock-infected (6 h)	Human airway epithelium cells	6	0	23935999
	H1N1-infected vs. mock-infected (12 h)	Human airway epithelium cells	11	6	23935999
	H1N1-infected vs. mock-infected (18 h)	Human airway epithelium cells	20	21	23935999
	H1N1-infected vs. mock-infected (24 h)	Human airway epithelium cells	22	29	23935999
	H1N1-infected vs. mock-infected (36 h)	Human airway epithelium cells	27	30	23935999
	H1N1-infected vs. mock-infected (48 h)	Human airway epithelium cells	18	16	23935999
GSE49840	H7N9-infected vs. mock-infected (3 h)	Calu-3 cells	1	2	24496798
	H7N9-infected vs. mock-infected (7 h)	Calu-3 cells	3	0	24496798
	H7N9-infected vs. mock-infected (12 h)	Calu-3 cells	8	3	24496798
	H7N9-infected vs. mock-infected (24 h)	Calu-3 cells	30	70	24496798
	H7N7-infected vs. mock-infected (3 h)	Calu-3 cells	1	0	24496798
	H7N7-infected vs. mock-infected (7 h)	Calu-3 cells	0	0	24496798
	H7N7-infected vs. mock-infected (12 h)	Calu-3 cells	5	4	24496798
	H7N7-infected vs. mock-infected (24 h)	Calu-3 cells	38	109	24496798
	H5N1-infected vs. mock-infected (3 h)	Calu-3 cells	1	2	24496798
	H5N1-infected vs. mock-infected (7 h)	Calu-3 cells	2	0	24496798
	H5N1-infected vs. mock-infected (12 h)	Calu-3 cells	6	0	24496798
	H5N1-infected vs. mock-infected (24 h)	Calu-3 cells	40	92	24496798
	H3N2-infected vs. mock-infected (3 h)	Calu-3 cells	1	3	24496798
	H3N2-infected vs. mock-infected (7 h)	Calu-3 cells	3	0	24496798
	H3N2-infected vs. mock-infected (12 h)	Calu-3 cells	12	16	24496798
	H3N2-infected vs. mock-infected (24 h)	Calu-3 cells	19	42	24496798
GSE59226	Influenza virus (H9N2)-infected vs. inactivate virus-infected	Human umbilical vein endothelial cells	27	137	25863179
GSE1377	Kaposi sarcoma-associated herpes virus-infected for 7 days vs. uninfected control	Primary human dermal endothelial cells	6	10	15220917

*MERS-CoV, Middle East respiratory syndrome coronavirus; SARS-CoV, severe acute respiratory syndrome coronavirus; H1N1, H7N9, H7N7, H5N1, H3N2, and H9N2, influenza viruses*.

**Table 4:** Six microarray datasets about endothelial cells in pro-atherogenic damage-associated molecular patterns (DAMPs) such as oxLDL, LPS, oxPAPC, and IFN treated conditions in the National Institutes of Health (NIH)–National Center for Biotechnology Information (NCBI)–Gene Expression Omnibus (GEO) datasets database (https://www.ncbi.nlm.nih.gov/gds/) were collected to analyze the changes of organelle interactions and vesicle-related gene.

**GEO ID**	**Comparison**	**Cell/tissue**	**Upregulated**	**Downregulated**	**PMID**
GSE5883	10-ng LPS stimulation for 4 h vs. without LPS stimulation	Human lung microvascular endothelial cells	8	7	NA
	10-ng LPS stimulation for 8 h vs. without LPS stimulation	Human lung microvascular endothelial cells	17	15	NA
	10-ng LPS stimulation for 24 h vs. without LPS stimulation	Human lung microvascular endothelial cells	9	14	NA
GSE3920	1,000 IU IFNα treated for 5 h vs. untreated control	Human umbilical vein endothelial cells	7	0	17202376, 19553003
	1,000 IU IFNβ treated for 5 h vs. untreated control	Human umbilical vein endothelial cells	11	0	17202376, 19553003
	1,000 IU IFNγ treated for 5 h vs. untreated control	Human umbilical vein endothelial cells	6	0	17202376, 19553003
GSE85987	Scrambled control siRNA vs. NOTCH1 siRNA	Human umbilical vein endothelial cells	0	0	29449332
	NOTCH1 siRNA + IL-1β treated for 24 h vs. scrambled siRNA	Human umbilical vein endothelial cells	0	1	29449332
GSE72633	NOTCH1 siRNA vs. scrambled control siRNA	Human aortic endothelial cells	3	6	26552708
	oxPAPC treated vs. scrambled control siRNA	Human aortic endothelial cells	8	6	26552708
GSE26953	Oscillatory shear vs. laminar shear (fibrosa)	Human aortic valvular endothelial cells	0	0	21705672
	Oscillatory shear vs. laminar shear (ventricularis)	Human aortic valvular endothelial cells	0	0	21705672
GSE39264	ApoE KO vs. WT	Mouse aortic endothelial cells	0	0	23990205
	LPS treated for 4 h vs. DMEM treated for 4 h	Mouse aortic endothelial cells	3	5	23990205
	oxLDL treated for 4 h vs. DMEM treated for 4 h	Mouse aortic endothelial cells	0	6	23990205
	oxPAPC treated for 4 h vs. DMEM treated for 4 h	Mouse aortic endothelial cells	7	7	23990205

*LPS, lipopolysaccharide; IFN, interferon; oxPAPC, oxidized 1-palmitoyl-2-arachidonoyl-sn-glycero-3-phosphocholine (inflammatory lipids); oxLDL, oxidized low-density lipoprotein; KO, knockout; WT, wild type; DMEM, Dulbecco's minimum essential medium*.

**Table 5:** Six microarray datasets about Treg cells in the National Institutes of Health (NIH)–National Center for Biotechnology Information (NCBI)–Gene Expression Omnibus (GEO) datasets database (https://www.ncbi.nlm.nih.gov/gds/) were collected to analyze the changes of organelle interactions and vesicle-related gene (*p* < 0.05, |logFC| > 1).

**GEO ID**	**Comparison**	**Tissue**	**Upregulated**	**Downregulated**	**PMID**
GSE37532	CD3^+^CD4^+^CD25^+^ Treg cells vs. CD3^+^CD4^+^CD25^−^ Tconv cells	LN	3	0	25550516
	CD3^+^CD4^+^CD25^+^ Treg cells vs. CD3^+^CD4^+^CD25^−^ Tconv cells	Visceral adipose tissue	12	0	25550516
GSE64909	CD4^+^CD25^+^Foxp3^+^ Treg vs. CD4^+^Foxp3^−^ Tconv (cold)	Brown adipose tissue	2	0	25714366
	CD4^+^CD25^+^Foxp3^+^ Treg vs. CD4^+^Foxp3^−^ Tconv (warm)	Brown adipose tissue	3	0	25714366
	CD4^+^CD25^+^Foxp3^+^ Treg vs. CD4^+^Foxp3^−^ Tconv (warm)	Spleen	3	1	25714366
GSE50096	CD4^+^ Foxp3^+^ Treg cells vs. CD4^+^ Foxp3^−^ Tconv cells (injured 4d)	Skeletal muscle	4	4	24315098
	CD4^+^ Foxp3^+^ Treg cells vs. CD4^+^ Foxp3^−^ Tconv cells (injured 14d)	Skeletal muscle	7	2	24315098
	CD4^+^ Foxp3^+^ Treg cells vs. CD4^+^ Foxp3^−^ Tconv cells (injured 4d)	Spleen	3	0	24315098
	CD4^+^ Foxp3^+^ Treg cells vs. CD4^+^ Foxp3^−^ Tconv cells (injured 14d)	Spleen	3	0	24315098
GSE119169	CD4^+^CD25^+^Foxp3^+^ Treg cells vs. CD4^+^CD25^+^Foxp3^−^ Tconv cells (Female)	Spleen	0	0	30962454
	CD4^+^CD25^+^Foxp3^+^ Treg cells vs. CD4^+^CD25^+^Foxp3^−^ Tconv cells (Male)	Spleen	1	0	
GSE20366	CD4^+^Foxp3^−^GFP^+^ T cells vs. CD4^+^Foxp3^−^GFP^−^ T cells	Small intestinal lamina propria	4	1	25550516
GSE15390	CD4^+^CD25^+^ Treg cells and CD4^+^CD25^−^ cells	Peripheral blood	1	1	21841785

**Table 6:** Fifteen microarrays of regulatory T cell regulator deficiency in the National Institutes of Health (NIH)–National Center for Biotechnology Information (NCBI)–Gene Expression Omnibus (GEO) datasets database (https://www.ncbi.nlm.nih.gov/gds/) were collected to analyze the expression changes of OCRGs (*p* < 0.05, |logFC| > 1).

**GEO ID**	**Comparison**	**Source**	**Cell**	**Upregulated**	**Downregulated**	**PMID**
GSE39864	Gata3 knockout vs. wild type	Spleen and LN	CD4^+^CD25^+^ Treg	0	0	22922362
GSE40493	Bcl6 knockout vs. wild type	Spleen and LN	FoxP3^+^ Treg	7	0	23053511
GSE27896	Hdac6 knockout vs. wild type	Lymphoid tissues	CD4^+^CD25^+^ Treg	1	0	21444725
GSE36095	Hdac9 knockout vs. wild type	Spleen and LN	CD4^+^CD25^+^ Treg	0	0	22715468
GSE11818	Dicer knockout vs. wild type/heterozygote	LN	CD4^+^ T cells	5	1	18725525
GSE14350	IL-2R defective vs. normal	Spleen	CD4^+^CD25^+^ Treg	1	3	19185518
GSE27143	Blimp1 gfp/gfp vs. +/gfp	Bone marrow	CD4^+^CD25^+^ Treg	3	6	21378976
GSE37532	Pparg– vs. Pparg+	LN	CD4^+^ T cells	0	0	22722857, 25550516
	Pparg– vs. Pparg+	VAT	CD4^+^ T cells	1	3	22722857, 25550516
GSE40273	Eos knockout vs. wild type	Spleen	CD4^+^CD25hi Treg	0	0	22961053
GSE40657	Foxo1 knockout vs. wild type	thymus, Spleen and LN	CD4^+^ T cells	6	1	23135404
GSE47989	Ep300–/– vs. wild type	Spleen	CD4^+^CD25^+^ Treg	0	1	23955711
GSE60318	deletion of p300 vs. wild type	Spleen and LN	CD4^+^CD25^+^ Treg	0	1	25154413
GSE18148	Cbfb-deficient vs. control	Peripheral CD4^+^CD25hi cells	FoxP3^+^ Treg	2	0	19800266
GSE32224	Trim28 knockout vs. wild type	Spleen and LN	CD4^+^CD62^+^CD25^+^ Treg	3	5	22544392
GSE61077	TCR knockout vs. wild type	spleen and LN	CD44 high Treg	1	1	25263123

*KO, knockout; WT, wild type; LN, lymph node; VAT, visceral adipose tissue*.

**Table 7:** Six microarrays of toll-like receptor deficiencies in the National Institutes of Health (NIH)–National Center for Biotechnology Information (NCBI)–Gene Expression Omnibus (GEO) datasets database (https://www.ncbi.nlm.nih.gov/gds/) were collected to analyze the expression changes of organelle crosstalk regulators (OCRGs) (p < 0.05, |logFC| > 1).

**GEO ID**	**Comparison**	**Cell/tissue**	**Upregulated**	**Downregulated**	**PMID**
GSE24935	Tlr2 KO+infection of SA vs. WT+infection of SA (6 h)	Glial	6	0	21901759
	Tlr2 KO+infection of SA vs. WT+infection of SA (12 h)	Glial	2	14	21901759
GSE56426	Tlr2 KO+injection of MTX vs. WT+injection of MTX	Proximal jejunum	24	23	25589072
GSE45861	Tlr2 KO+infection of European strain (P1/7) of *Streptococcus suis* vs. WT+infection of European strain (P1/7) of *S. suis*	Spleen	5	1	23724118
	Tlr2 KO+infection of Chinese strain (SC84) of *S. suis* vs. WT+infection of Chinese strain (SC84) of *S. suis*	Spleen	4	6	23724118
GSE31066	Tlr4 KO+LPS treatment vs. WT+LPS treatment	Macrophage	4	10	21865549
	Tlr4 KO+lipid A treatment vs. WT+lipid A treatment	Macrophage	4	10	21865549
GSE103750	Tlr7 KO+infection of STM vs. WT+infection of STM	Macrophage	1	1	29616197
GSE92358	Tlr3/7/9 KO+injection with MOPC cells vs. WT+injection with MOPC cells (day 4)	Tumor tissues	5	7	28300057
	Tlr3/7/9 KO+injection with MOPC cells vs. WT+injection with MOPC cells (day 6)	Tumor tissues	7	8	28300057

*KO, knockout; WT, wild type; SA, Staphylococcus aureus; MTX, methotrexate; STM, Salmonella enterica serovar Typhimurium; MOPC, murine oropharyngeal carcinoma cell*.

**Table 8:** Reactive oxygen species (ROS) negative regulator Nrf2 deficiency microarray (GSE7810) in the National Institutes of Health (NIH)–National Center for Biotechnology Information (NCBI)–Gene Expression Omnibus (GEO) datasets database (https://www.ncbi.nlm.nih.gov/gds/) was collected to analyze the expression changes of organelle interactions and vesicle-related genes (*p* < 0.05, |logFC| > 1).

**GEO ID**	**Comparison**	**Cell**	**Upregulated**	**Downregulated**	**PMID**
GSE7810	Nrf2–/– vs. Nrf2+/+	Mouse type II cells	14	7	17895394

**Table 9:** Caspase-1 deficiency microarrays in the National Institutes of Health (NIH)–National Center for Biotechnology Information (NCBI)–Gene Expression Omnibus (GEO) datasets database (https://www.ncbi.nlm.nih.gov/gds/) were collected to analyze the expression changes of organelle crosstalk regulators (OCRGs) (*p* < 0.05, |logFC| > 1).

**GEO ID**	**Comparison**	**Cell/tissue**	**Upregulated**	**Downregulated**	**PMID**
GSE25205	Casp1 KO vs. WT	Epididymal white adipose tissue	2	3	21876127
GSE32515	Casp1 KO vs. WT	Duodenum	1	1	23160218
	Casp1 KO vs. WT	Jejunum	2	1	23160218
	Casp1 KO vs. WT	Ileum	1	0	23160218
	Casp1 KO vs. WT	Liver	2	1	23160218

*KO, knockout; WT, wild type*.

**Table 10:** Organelle crosstalk regulators (OCRGs) were examined in 28 types of cancers.

**Cancer in TCGA**	**Detail**	**No. of OCRGs (a)**		**No. of total differently**		**Ratio (a/b)%**	
				**expressed genes (b)**			
		**Up**	**Down**	**Up**	**Down**	**Up**	**Down**
ACC	Adrenocortical carcinoma	4	28	545	2,544	0.73	1.10
BLCA	Bladder urothelial carcinoma	6	20	855	1,888	0.70	1.06
BRCA	Breast invasive carcinoma	19	19	1,418	2,137	1.34	0.89
CESC	Cervical squamous cell carcinoma and endocervical adenocarcinoma	18	44	1,851	3,907	0.97	1.13
COAD	Colon adenocarcinoma	33	25	2,649	2,671	1.25	0.94
DLBC	Lymphoid neoplasm diffuse large B-cell lymphoma	133	12	8,804	945	1.51	1.27
ESCA	Esophageal carcinoma	40	12	2,633	1,412	1.52	0.85
GBM	Glioblastoma multiforme	71	24	5,208	2,436	1.36	0.99
HNSC	Head and neck squamous cell carcinoma	17	9	1,488	589	1.14	1.53
KICH	Kidney chromophobe	6	38	741	3,451	0.81	1.10
KIRC	Kidney renal clear cell carcinoma	18	10	1,626	1,322	1.11	0.76
KIRP	Kidney renal papillary cell carcinoma	13	12	986	1,421	1.32	0.84
LAML	Acute myeloid leukemia	30	49	4,706	3,254	0.64	1.51
LGG	Brain lower grade glioma	63	18	3,977	1,762	1.58	1.02
LIHC	Liver hepatocellular carcinoma	19	5	1,475	722	1.29	0.69
LUAD	Lung adenocarcinoma	1	32	1,109	3,130	0.09	1.02
LUSC	Lung squamous cell carcinoma	14	47	1,920	4,035	0.73	1.16
OV	Ovarian serous cystadenocarcinoma	24	54	2,618	5,014	0.92	1.08
PAAD	Pancreatic adenocarcinoma	150	**3**	8,730	478	1.72	0.63
PRAD	Prostate adenocarcinoma	5	20	681	2,324	0.73	0.86
READ	Rectum adenocarcinoma	37	29	2,829	2,935	1.31	0.99
SKCM	Skin cutaneous melanoma	34	33	2,543	3,907	1.34	0.84
STAD	Stomach adenocarcinoma	55	7	3,745	896	1.47	0.78
TGCT	Testicular germ cell tumors	31	65	2,528	11,265	1.23	0.58
THCA	Thyroid carcinoma	6	38	659	3,402	0.91	1.12
THYM	Thymoma	166	10	11,809	934	1.41	1.07
UCEC	Uterine corpus endometrial carcinoma	20	49	2,036	5,088	0.98	0.96
UCS	Uterine carcinosarcoma	19	51	1,916	4,692	0.99	1.09

*Differentially expressed gene lists of these 28 subtypes of cancers were downloaded from EGPIA2 database (http://gepia2.cancer-pku.cn/#index), in which the dataset sources were from The Cancer Genome Atlas (TCGA)/Genotype-Tissue Expression (GTEx) data (adjp < 0.05, |logFC| > 1)*.

*Up, upregulated OCRGs; Down, downregulated OCRGs*.

**Table 11:** Nineteen microarrays of deficiencies of oncogene and tumor suppressor in the National Institutes of Health (NIH)–National Center for Biotechnology Information (NCBI)–Gene Expression Omnibus (GEO) datasets database (https://www.ncbi.nlm.nih.gov/gds/) were collected to analyze the expression changes of organelle crosstalk regulators (OCRGs) (*p* < 0.05, |logFC| > 1).

**GEO ID**	**Comparison**	**Organism**	**Cells/tissue**	**Upregulated**	**Downregulated**	**PMID**
GSE30049	IKK2 knockout vs. wild type	*Mus musculus*	Lung tumor cell lines	5	5	22327365
	IKK2 knockout vs. wild type	*M. musculus*	Lung tumor nodules	1	0	22327365
GSE46250	IKK complex inhibition vs. control	*M. musculus*	Leukemia cells	1	0	24054986
GSE46251	IKK complex inhibition vs. control	*Homo sapiens*	Leukemia cells	0	1	24054986
GSE71444	IKK2 knockdown vs. scramble control	*H. sapiens*	Human MDA-MD-231 cells	0	0	29662632
	RELA knockdown vs. scramble control	*H. sapiens*	Human MDA-MD-231 cells	1	2	29662632
GSE36568	Rela knockout vs. wild type	*M. musculus*	Lung carcinoma cells	5	2	NA
GSE54645	JAK2 knockdown vs. control	*H. sapiens*	AML cell line	0	1	24740812
GSE44652	STAT1 knockdown vs. control	*H. sapiens*	Human T-All cell line	1	5	23471820
GSE75325	Stat3 knockout vs. wild type	*M. musculus*	Mouse mammary tumors	2	1	26719528
GSE48124	STAT3 knockdown vs. control	*H. sapiens*	Urothelial cancer cell line	0	1	24525232
GSE34760	Tp53 knockout vs. wild type	*M. musculus*	Liver tumors	23	48	22342966
	Tp53 knockout vs. wild type	*M. musculus*	Liver	6	13	22342966
GSE40545	Tp53, Rb double knockout vs. wild type	*M. musculus*	Mammary epithelial cells	7	1	25602521
	Tp53 knockout vs. wild type	*M. musculus*	Mammary epithelial cells	15	4	25602521
GSE62694	Mutant Tp53 vs. control	*M. musculus*	Oviductal cells	4	1	25810107
GSE76296	Mutant Tp53 vs. wild type	*M. musculus*	Neural stem cells	0	1	26984279
GSE70262	Tp53 knockout vs. wild type	*M. musculus*	Small intestine	0	0	18533991
	APC knockout vs. wild type	*M. musculus*	Small intestine	6	3	18533991
GSE39955	Tp53 knockout vs. control	*M. musculus*	Neu primary tumor	6	4	25330770
	PTEN knockout vs. control	*M. musculus*	Neu primary tumor	7	4	25330770
GSE54265	PTEN knockdown vs. control	*H. sapiens*	Breast cells lines	5	1	24553445
GSE68869	PTEN knockdown vs. control	*H. sapiens*	Lung adenocarcinoma cells	1	0	25995385
GSE120478	PTEN null vs. wild type	*M. musculus*	Mouse embryonic fibroblasts	1	0	31169889
GSE121217	PTEN knockdown vs. control	*H. sapiens*	Lung adenocarcinoma cells	0	2	31461649

### Metascape Pathway Analysis

We utilized Metascape Pathway Analysis (MPA; http://metascape.org/gp/index.html#/main/step1) (73) to characterize molecular and cellular functions related to the identified genes in our microarray analysis. Differentially expressed genes were identified and uploaded into MPA for analysis. The core and pathways analysis was used to identify molecular and cellular pathways, as we have previously reported (23).

### Protein–Protein Interaction Analysis

Protein–protein interaction (PPI) networks were generated from STRING database (https://string-db.org/). Enrichment analysis results of the shared gene and top 10 connected proteins were downloaded and visualized by using Cytoscape software 3.7.2 (https://cytoscape.org/) (74).

### Immune Infiltrate Analyses

TISIDB database (75) (http://cis.hku.hk/TISIDB/index.php) and Gene Set Cancer Analysis (GSCA) database (http://bioinfo.life.hust.edu.cn/GSCA/#/immune) (76) were used in the immune cell infiltrate analyses.

### Statistical Analysis of Microarray Data

Twelve housekeeping genes including CHMP2A, EMC7, GPI, PSMB2, PSMB4, RAB7A, SNRPD3, VPS29, VCP, ACTB, RPL27, and OAZ1 (Supplementary Table 3 of Housekeeping Genes) in all 85 GEO datasets regardless of species were chosen for this study. The housekeeping gene list was extracted from the list provided by Eisenberg and de Jonge (77, 78). Briefly, the mean fold change (FC) of housekeeping genes between treatment and control groups varies from 0.72 to 1.55. As this variation was out of the range of FC < 0.5 or FC > 2 (|log2FC| > 1), we concluded that the datasets were of high quality. The target genes with expression changes more than 2-fold in microarrays were defined as the upregulated genes, while genes with their expression decreased more than 2-fold in microarrays were defined as downregulated genes (*p* < 0.05, |log2FC| > 1).

## Results

### The Ratios of Upregulated vs. Downregulated Organelle Crosstalk Regulators in Diseases Are Different, and Differentially Expressed Organelle Crosstalk Regulators Are Shared in Diseases

**Figure 1:**
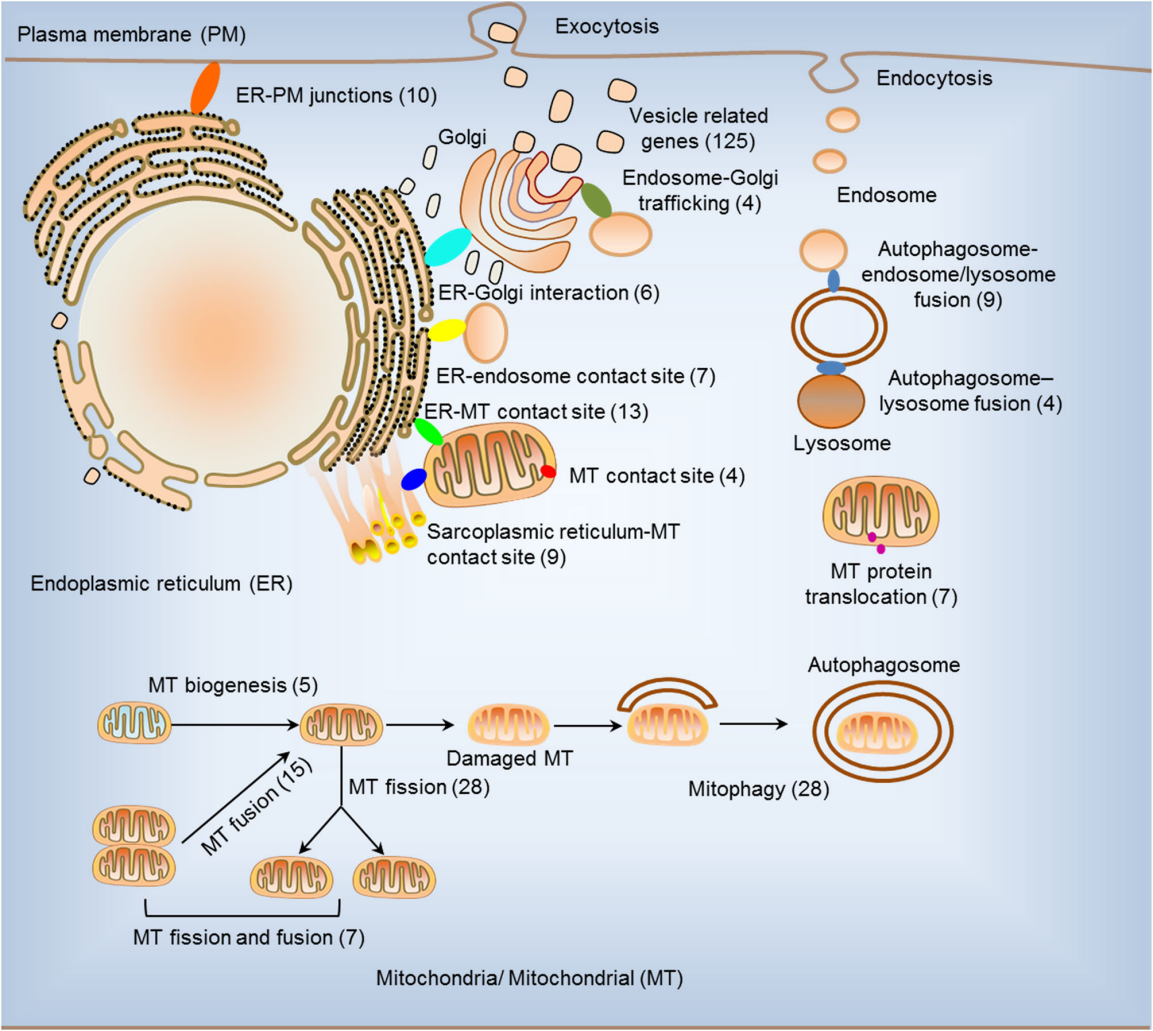
Two hundred sixty organelle crosstalk regulators (OCRGs) in 16 groups were analyzed. There are 16 classification or function of these genes, including nine mitochondria (MT)-related gene populations: MT biogenesis (five genes), MT fusion (15 genes), MT fission (28 genes), MT fission and fusion (seven genes), mitophagy (28 genes), ER–MT contact site (13 genes), MT contact site (four genes), sarcoplasmic reticulum–MT contact site (nine genes), and MT protein translocation (seven genes); three endoplasmic reticulum (ER)-related populations except ER–MT contact site: ER–endosome contact site (seven genes), ER–Golgi interact genes (6), and ER–PM junctions (10 genes); endosome–Golgi trafficking (four genes); autophagosome–lysosome fusion (four genes); autophagosome–endosome/lysosome fusion (nine genes); vesicle-related (125 genes) coded proteins are enhanced by the Human Protein Atlas. Because some genes have more than one function, the final selection is 260 non-repetitive genes. The detailed gene list is in Table 1.

We hypothesized that pathological conditions significantly modulate the expressions of organelle crosstalk regulators in disease-specific and cell type-specific manner. To examine this hypothesis, we collected 260 organelle crosstalk regulators (regulatomic genes and OCRGs) in 16 groups (Figure 1) and were effectively detected (Table 1). Among the OCRGs, 19 genes can be classified into two groups, and one gene can be classified into three groups (Supplementary Figure 1), so that a total of 281 genes were calculated when using a donut chart to show percentages of the gene classifications. The OCRGs included (1) five genes in MT biogenesis (2%), (2) 28 genes in MT fission (10%), (3) 15 genes in MT fusion (5%), (4) seven genes in MT fission and fusion (2%), (5) 28 genes in mitophagy (10%), (6) seven genes in MT protein translocation (2%), (7) four genes in MT contact site (1%), (8) 13 genes in ER–MT contact (5%), (9) nine genes in sarcoplasmic reticulum–MT (3%), (10) 10 genes in ER–plasma conjunctions (3%), (11) six genes in ER–Golgi interaction (2%), (12) seven genes in ER–endosome (2%), (13) four genes in autophagosome–lysosome fusion (1%), (14) nine genes in autophagosome–endosome/lysosome fusion (3%), (15) four genes in endosome–Golgi trafficking (1%), and (16) 125 genes in vesicle (44%). To confirm the functional focuses of the OCRGs, we performed the MPA using the database (http://metascape.org/gp/index.html#/main/step1) (73). Our analysis showed that 260 OCRGs were indeed enriched in MT organization, MT fission, autophagy, vesicle organization, and organelle organization, which can be viewed in Supplementary Figure 2. As shown in Table 2, the expressions of 260 OCRGs were examined in four major categories of diseases, in a total of 23 types of diseases. These diseases and cell types were (a) three types of AIs such as lung injury (whole blood), septic shock (whole blood), and severe trauma (monocytes, leukocytes, and T cells); (b) nine types of MDs including obesity (adipocytes and adipose stem cells) and metabolically healthy obesity (MHO; subcutaneous adipose). Based on the criteria, patients with MHO have no metabolic syndrome (MetS) and insulin resistance (IR) (20) and are metabolically unhealthy obese (MUO; subcutaneous adipose). The difference between MUO and MHO can be found as cited (79), obese with IR (adipocytes), obese with insulin sensitivity (IS) (adipocytes), type 2 diabetes (T2D; liver, skeletal muscle, subcutaneous adipose, and visceral adipose), atherosclerosis (carotid artery plaques, macrophages, and T cells), atherosclerosis and familial combined hyperlipidemia (FCH; plaques and monocytes), and familial hypercholesterolemia (FHC; lymphoblastic cells); (c) seven types of ADs including rheumatoid arthritis (RA; macrophages), acute cutaneous lupus (ACLE; skin), chronic cutaneous lupus (CCLE; skin), psoriasis (skin), subacute cutaneous lupus (SCLE; skin), ulcerative colitis (UC; colon/rectum and PBMCs), and Crohn's disease (CD; PBMC); and (d) *four* types of organ failures (OFs) such as heart failure (left ventricle), hepatitis B virus-associated acute liver failure (liver), end-stage renal failure (ESRF; whole blood), and chronic kidney disease (CKD) hemodialysis (PBMC). In a total of 39 comparison datasets, we made the following findings: (i) the expressions of OCRGs were modulated except obese with IS; (ii) the expressions of OCRGs in AIs such as septic shock, trauma, ADs, and OFs except heart failure were changed more than those in MDs; (iii) among 38 datasets (except obese IS vs. lean), the downregulations of OCRGs were more than the upregulations of OCRGs in 21 datasets; (iv) the most significant downregulation of OCRGs was found in monocytes, leukocytes, and T cells in severe trauma, skeletal muscle in T2D, and whole blood and PBMC in end-stage renal disease and CKD hemodialysis; (v) the expression changes of OCRGs in T cells in severe trauma were much more than those in T cells in atherosclerosis, suggesting that OCRG expression changes were more disease-dependent rather than cell type-dependent; and (vi) adipocytes had less OCRG expression changes than other cell types.

**Figure 2:**
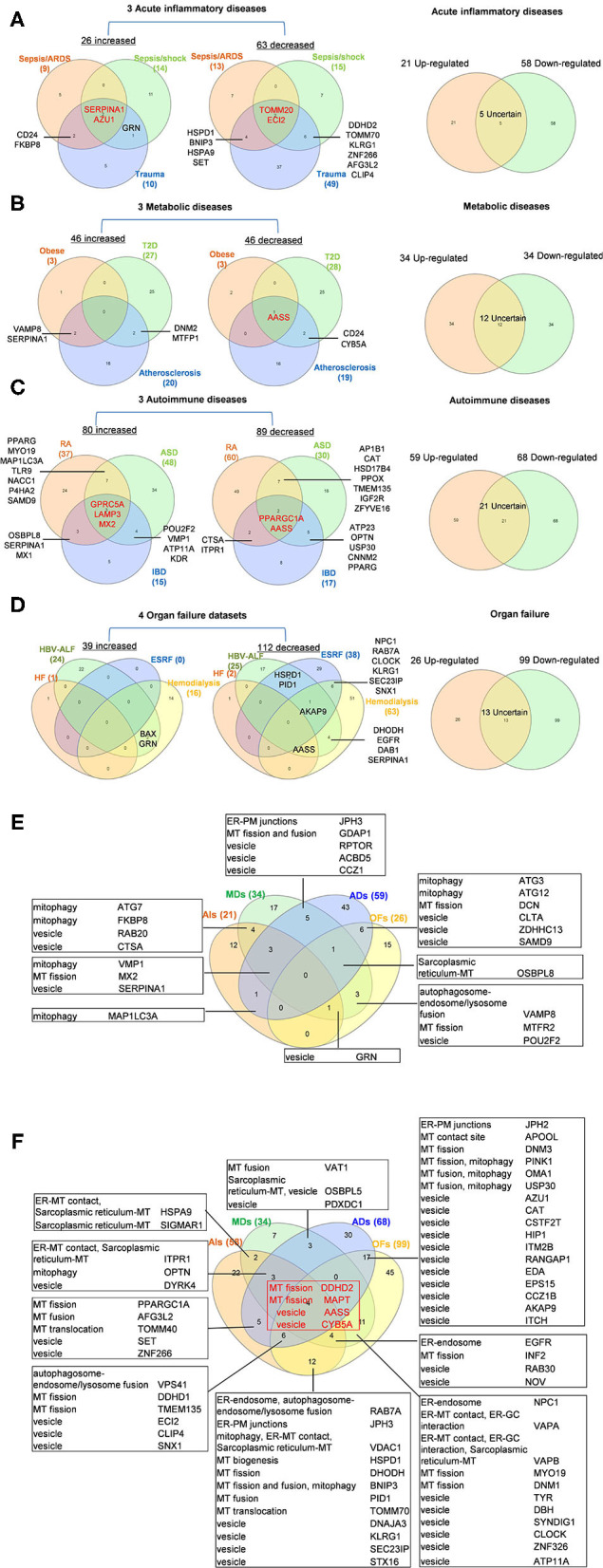
Venn diagram shows that there are several shared OCRGs in acute inflammations (AIs), metabolic diseases (MDs), autoimmune diseases (ADs), and organ failures (OFs). **(A)** Venn diagram shows 17 genes are shared among acute inflammatory diseases (AIs). SERPINA1 and AZU1 are shared upregulated genes; TOMM20 and ECI2 are shared downregulated genes by sepsis/ARDS, sepsis/shock, and trauma. CD24, FKBP8, and GRN are shared upregulated genes by sepsis and trauma. HSPD1, BNIP3, HSPA9, SET, DDHD2, TOMM70, KLRG1, ZNF266, AFG3L2, and CLIP4 are 10 genes that are commonly downregulated in sepsis and trauma groups. After removing five uncertain genes (upregulated in one type of disease and downregulated in another type of disease), 21 genes are upregulated and 58 genes are downregulated in AIs. **(B)** Venn diagram shows seven genes are shared among metabolic diseases (MDs). VAMP8 and SERPINA1 are shared upregulated genes by obese and atherosclerosis; DNM2 and MTFP1 are shared upregulated genes by type 2 diabetes (T2D) and atherosclerosis. AASS is shared a downregulated gene by three metabolic diseases, and CD24 and CYB5A are shared downregulated genes by T2D and atherosclerosis. After removal of 12 uncertain genes, 34 genes are upregulated and 34 genes are downregulated in MDs. **(C)** Venn diagram shows 33 genes are shared among autoimmune diseases. GPRC5A, LAMP3, and MX2 are shared upregulated genes; PPARGC1A and AASS are shared downregulated genes by rheumatoid arthritis (RA), autoimmune skin disease (ASD), and inflammatory bowel disease (IBD). Other 28 genes are common genes between two autoimmune diseases. After removal of 21 uncertain genes, 59 genes are upregulated, and 68 genes are downregulated in ADs. **(D)** Venn diagram shows 16 genes are shared in organ failure datasets. BAX and GRN are shared upregulated genes by hepatitis B virus-associated acute liver failure (HBV-ALF). AKAP9 is the common downregulated gene in HBV-ALF, end-stage renal failure (ESRF), and hemodialysis; NPC1, RAB7A, CLOCK, KLRG1, SEC23IP, and SNX1 are the common downregulated genes in ESRF and hemodialysis. After removal of 13 uncertain genes, 26 genes are upregulated and 99 genes are downregulated in OFs. **(E)** Venn diagram shows the shared and exclusive upregulated and downregulated OCRGs in these four diseases. In upregulated OCRGs, a total of 24 genes were shared by two or three different diseases. AIs and MDs shared four genes, two vesicle-related genes (RAB20 and CTSA), and two mitophagy genes (FKBP8 and ATG7). AIs and ADs shared mitophagy gene (MAP1LC3A). AIs, MDs, and OFs shared vesicle genes (GRN). MDs and OFs shared three genes autophagosome–endosome/lysosome fusion regulator (VAMP8), MT fission gene (MTFR2), and vesicle gene (POU2F2). AIs, MD, and ADs shared mitophagy (VMP1), MT fission (MX2), and vesicle (SERPINA1) genes. MDs, ADs, and OFs shared sarcoplasmic reticulum–MT gene (OSBPL8). ADs and OFs shared two mitophagy genes (ATG3 and ATG12), one MT fission gene (DCN), and three vesicle genes (CLTA, ZDHHC13, and SAMD9). MDs and ADs shared five genes including one ER–PM junctions gene (JPH3), one MT fission and fusion regulator (GDAP1), and three vesicle genes (RPTOR, ACBD5, and CCZ1). Other exclusive upregulated genes in these four diseases are listed in Supplementary Table 4A. **(F)** In downregulated OCRGs, a total of 67 genes were shared by two or more than two different diseases. Two MT fission genes (DDHD2 and MAPT) and two vesicle genes (AASS and CYB5A), were shared by four types of diseases (red box). ADs and OFs shared 17 OCRGs, including one ER–PM junction regulator (JPH2) and one MT contact site gene (APOOL), two MT fission regulators (DNM3 and PINK1) (also mitophagy regulator), two genes with MT fusion and mitophagy function (OMA1 and USP30), and 11 vesicle genes. AIs and OFs shared 12 OCRGs, RAB7A (ER–endosome and autophagosome–endosome/lysosome fusion), JPH3 (ER–PM junctions), VDAC1 (mitophagy, ER–MT contact, and sarcoplasmic reticulum–MT), HSPD1 (MT biogenesis), DHODH (MT fission), BNIP3 (MT fission and fusion, and mitophagy), PID1 (MT fusion), TOMM70 (MT translocation), and four vesicle genes (DNAJA3, KLRG1, SEC23IP, and STX16). MDs and OFs shared 11 OCRGs including ER–endosome genes (NPC1), ER–MT contact and ER–GC interaction regulator VAPA, ER–MT contact, ER–GC interaction and sarcoplasmic reticulum–MT regulator VAPB, two MT fission genes MYO19 and DNM1, and six vesicle genes. AIs and MDs, AIs and ADs, and MDs and ADs shared two, five, and three downregulated OCRGs, respectively. AIs, MDs, and ADs shared three downregulated OCRGs, ITPR1 (ER–MT contact, sarcoplasmic reticulum–MT), OPTN (mitophagy), and DYRK4 (vesicle gene). AIs, MDs, and OFs shared four OCRGs (EGFR, INF2, RAB30, and NOV). AIs, ADs, and OFs shared six OCRGs, VPS41 (autophagosome–endosome/lysosome fusion), two MT fission regulators DDHD1 and TMEM135, and three vesicle regulators (ECI2, CLIP4, and SNX1). Other exclusive downregulated genes in these four diseases are listed in Supplementary Table 4B. Abbreviations: AIs, acute inflammations; MDs, metabolic diseases; ADs, autoimmune diseases; OFs, organ failures; ARDS, acute respiratory distress syndrome; T2D, type 2 diabetes; HF, heart failure; HBV-ALF, hepatitis B virus-associated acute liver failure; ESRF, end-stage renal failure.

To determine whether among 260 OCRGs upregulated and downregulated regulators were shared in disease categories, we performed a Venn diagram analysis. As shown in Figures 2A–D, in 26 upregulated OCRGs in AIs, the upregulation of two regulators, SERPINA1 and AZU1, was shared among acute respiratory distress syndrome (ARDS), sepsis, and trauma; upregulation of CD24 and FKBP8 was shared among ARDS and trauma; and upregulation of GRN was shared between sepsis and trauma. In 46 upregulated OCRGs in MDs, upregulation of two regulators VAMP8 and SERPINA1 was shared between obese and atherosclerosis; and two upregulated regulators DNM2 and MTFP1 were shared between T2D and atherosclerosis. In 80 upregulated OCRGs in ADs, three regulators (i.e., GPRC5A, LAMP3, and MX2) were shared among RA, autoimmune skin disease (ASD), and inflammatory bowel disease (IBD); three regulators (i.e., OSBPL8, SERPINA1, and MX1) were shared between RA and IBD; seven regulators (i.e., PPARG, MYO19, MAP1LC3A, TLR9, NACC1, P4HA2, and SAMD9) were shared between RA and ASD; and four regulators (i.e., POU2F2, VMP1, ATP11A, and KDR) were shared between ASD and IBD. In 39 upregulated OCRGs in OFs, two regulators (i.e., BAX and GRN) were shared between CKD hemodialysis and hepatitis B virus liver failure. These results have demonstrated that first, the majority of the upregulated OCRGs was disease-specific; second, ADs shared more upregulated OCRGs than other diseases; and third, the ratios of upregulated OCRGs vs. downregulated OCRGs were 1:2.8 in AIs, 1:1 in MDs, 1:1.2 in ADs, and 1:3.8 in OFs, suggesting that AIs and OFs had downregulated OCRGs much more than those of upregulated in comparison with those of others. We then used a Venn diagram to analyze the overlapping OCRGs and their classifications among AIs, MDs, ADs, and OFs (Figure 2E) and listed the exclusively expressed OCRGs upregulated and downregulated in these four types of diseases in Supplementary Tables 4A,B. The results showed that a total of 24 upregulated OCRGs were shared by two or three different diseases. AIs and MDs, and AIs and ADs shared three and one OCRGs, respectively. MDs and ADs, and MDs and OFs shared five and three OCRGs, respectively. ADs and OFs shared six OCRGs. AIs, MDs, and ADs shared three OCRGs. AIs, MDs, and OFs shared one OCRG. MDs, ADs, and OFs shared one OCRG. There were 12, 17, 43, and 15 exclusively upregulated OCRGs in AIs, MDs, ADs, and OF diseases, respectively (Supplementary Table 4A). A total of 67 downregulated OCRGs were shared by two or more than two different diseases (Figure 2F). The four OCRGs including two MT fission genes (i.e., DDHD2 and MAPT) and two vesicle genes (i.e., AASS and CYB5A) were downregulated by four types of diseases. OFs and other diseases (AIs, MDs, and ADs) shared more downregulated OCRGs (12, 11, and 17, respectively). There were 22, 7, 30, and 45 downregulated OCRGs in AIs, MDs, ADs, and OFs, respectively (Supplementary Table 4B). These results showed that differentially classified OCRGs were shared in different diseases.

### Sepsis and Trauma-Upregulated Organelle Crosstalk Regulator Groups Are Classified as the Acute Crisis-Handling Organelle Crosstalk Regulators; and Organ Failure-Upregulated Organelle Crosstalk Regulators Groups Are Classified as the Cell Failure-Handling Organelle Crosstalk Regulators

**Figure 3:**
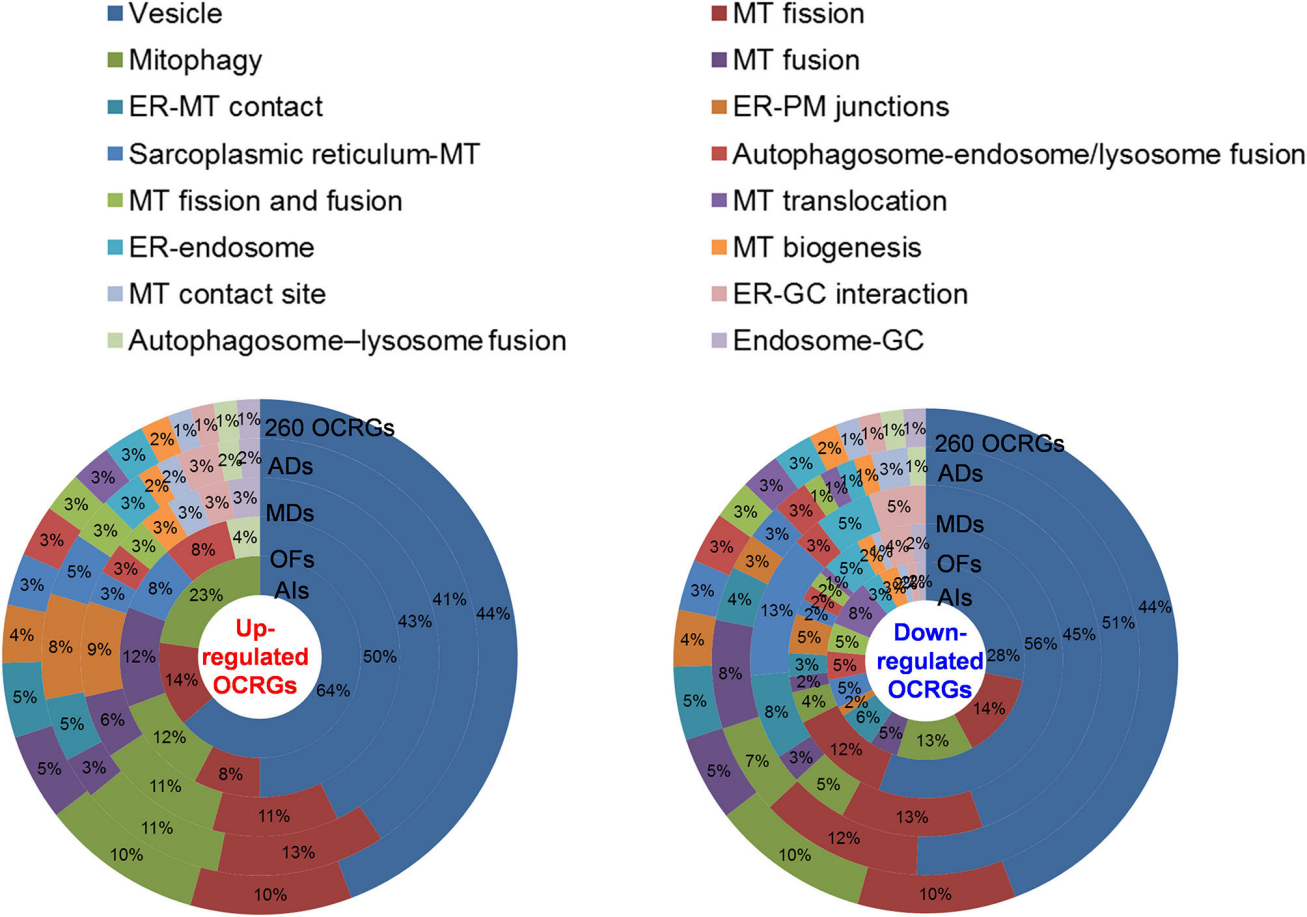
Donut chart shows the different classification/function ratios of upregulated and downregulated OCRGs in acute inflammations (AIs), metabolic diseases (MDs), autoimmune diseases (ADs), and organ failures (OFs). From the center to the outside of the donut chart, the circle represents the ratios of OCRGs in acute inflammations (AIs), organ failures (OFs), metabolic diseases (MD), autoimmune diseases (ADs), and all 260 OCRGs in turn. Groups of upregulated OCRGs in AIs (3) and OFs (7) are less than in MDs (12) and OFs (14) (left). In the 21 upregulated genes in AIs, vesicle-related genes account for 64%, and mitophagy and mitochondrial (MT) fission genes account for 14 and 23%, respectively. In the 26 upregulated genes in OFs, the top 3 high-number genes are vesicle- (50%), mitophagy- (12%), and MT fusion-related (12%) OCRGs. In the 34 upregulated genes in MDs, vesicle- (43%), MT fission- (11%), and mitophagy-related (11%) genes are the top 3 genes with high proportion. In the 59 upregulated genes of ADs, vesicle-related genes account for 41%, MT fission genes account for 13%, and mitophagy genes account for 11%. The downregulated OCRGs distributed in AIs, OFs, MDs, and ADs are 15, 15, nine, and 14 groups, respectively (right). In the 58 downregulated genes in AIs, the top 3 high-number genes are vesicle- (28%), MT fission- (14%), and mitophagy-related (13%) genes. In the 99 downregulated genes in OFs, the proportions of vesicle (56%), MT fission (12%), and ER–plasma membrane (PM) junctions (5%) genes are higher than those of others. In the 34 downregulated genes in MDs, the numbers of vesicle (45%), MT fission (13%), and endoplasmic reticulum (ER)–MT contact genes (13%) are higher. In ADs, vesicle (51%), MT fission (12%), and MT fusion genes (8%) are the top 3. These results show that different diseases have different expression patterns of OCRGs and that the classification proportion of upregulated and downregulated genes in the same type of diseases is different, which may explain the different functions of OCRGs in the occurrence and development of diseases. Abbreviations: ER, endoplasmic reticulum; GC, Golgi complex; MT, mitochondria; PM, ER–plasma membrane.

We then hypothesized that every major disease group modulates differentially 16 OCRG groups. We used the donut chart analysis. As shown in Figure 3, the 21 upregulated OCRGs in AIs were distributed in three categories including vesicle (64%), mitophagy (23%), and MT fission (14%). The 26 upregulated OCRGs in OFs were distributed in seven groups including vesicle (50%), MT fusion (12%), mitophagy (12%), MT fission (8%), sarcoplasmic reticulum–MT (8%), autophagosome–endosome/lysosome fusion (8%), and autophagosome–lysosome fusion (4%). The 34 upregulated OCRGs in MDs were distributed in 12 groups, including vesicle (43%), MT fission (11%), mitophagy (11%), ER–PM junctions (9%), and MT fusion (6%), and in the rest of the seven groups (3% of each). The 59 upregulated OCRGs in ADs were distributed in 14 groups, including vesicle (41%), MT fission (13%), mitophagy (11%), ER–PM junctions (8%), ER–MT contact (5%), and sarcoplasmic reticulum–MT (5%), and in the rest of other groups (2–3% of each). However, the downregulated OCRGs in AIs, OFs, MDs, and ADs were distributed in 15, 15, 9, and 14 groups, respectively. These results have demonstrated that *first*, vesicle, MT fission, and mitophagy were three top groups of organelle crosstalk regulators upregulated in all four major diseases. Since these three groups of OCRGs are only upregulated in AIs, sepsis, and trauma, we also classify those three groups including vesicle, MT fission, and mitophagy as the cell crisis-handling OCRGs, at least in the partial functions of the three groups, which were well-correlated with a previous report that mitophagy regulator PINK1 is an MT quality control gate keeper (80); *second*, ER crosstalk regulators were only upregulated in MD and AD groups but not in AIs and OFs; *third*, a few groups of OCRGs were upregulated in AIs (three groups) and OFs (seven groups), but the other two disease groups had more groups of OCRGs upregulated (MDs had 12 groups and ADs had 14 groups). Therefore, similar to the cell crisis-handling OCRGs that we defined, we also classify vesicle, MT fission, mitophagy, sarcoplasmic reticulum–MT, MT fusion, autophagosome–lysosome fusion, and autophagosome/endosome–lysosome fusion as the cellular failure-handling OCRGs, at least in the partial functions of seven groups; *fourth*, MT fusion regulators and sarcoplasmic reticulum–MT regulators were upregulated in MDs, ADs, and OFs; and *fifth*, AIs, MDs, ADs, and OFs downregulated 15, 9, 14, and 15 OCRG groups, respectively, suggesting that AIs and OFs upregulate less OCRG groups but downregulate more OCRG groups than MDs and ADs.

### The Majority of Upregulated Pathways Are Disease Group-Specific, and Some Upregulated Pathways Are Shared by Acute Inflammations, Metabolic Diseases, Autoimmune Diseases, and Organ Failures

**Figure 4:**
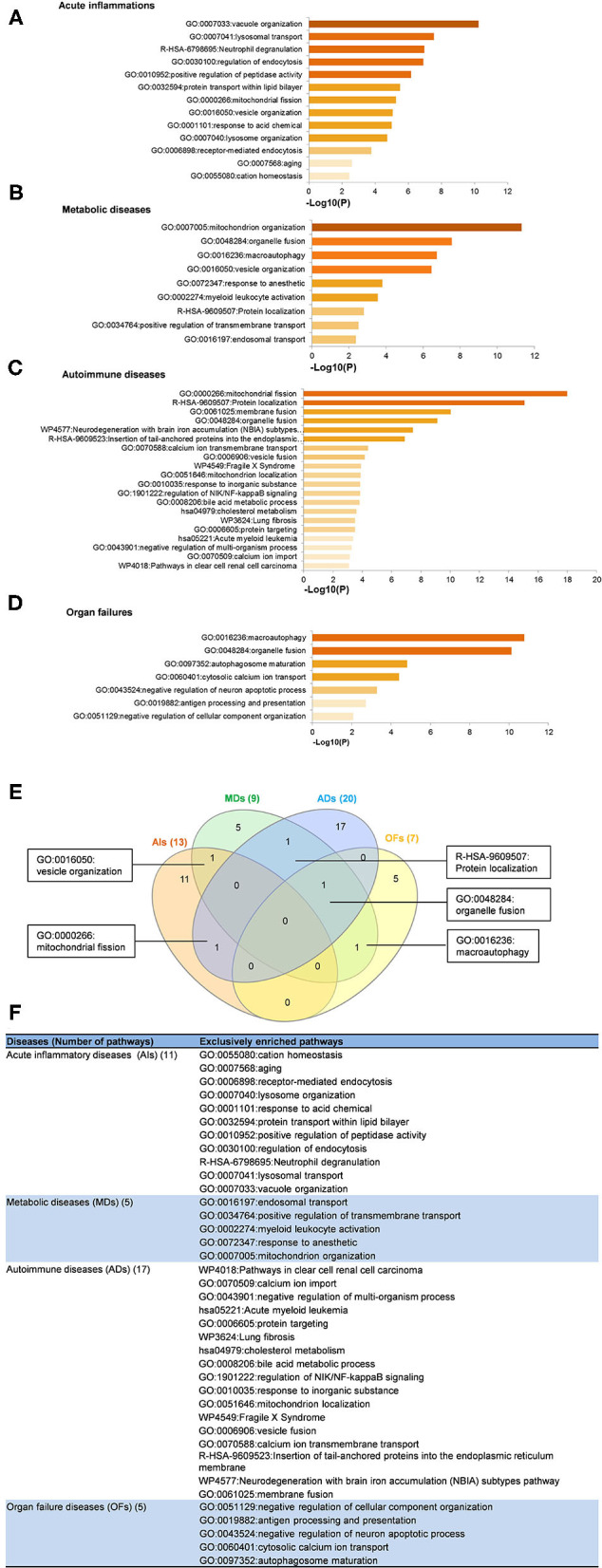
Enrichment analysis results show that several pathways of upregulated OCRGs were involved in acute inflammations (AIs), metabolic diseases (MDs), autoimmune diseases (ADs), and organ failures (OFs). Gene Ontology (GO)-based enrichment of significant upregulated OCRGs in different types of diseases was analyzed using Metascape software (http://metascape.org/gp/index.html#/main/step1; PMID: 30944313). **(A)** There are 14 significant GO enrichment results in 21 upregulated OCRGs in acute inflammations (AIs). The top 3 GO enrichments of OCRGs are vacuole organization, lysosomal transport, and neutrophil degranulation. Except for neutrophil degranulation, relative to acute inflammatory diseases, regulation of endocytosis and receptor-mediated endocytosis are significant GO enrichment results, which show that endocytosis is increased in AIs. **(B)** There are nine significant GO enrichment results in 34 upregulated OCRGs in metabolic diseases (MDs). The top 3 GO enrichments of OCRGs are mitochondrion organization, organelle fusion, and macroautophagy. Some genes are enriched in myeloid leukocyte activation, which suggested that these genes are involved in chronic low-grade inflammation. **(C)** There are 20 significant GO enrichment results in 60 upregulated OCRGs in autoimmune diseases (ADs). Mitochondrial fission, protein localization, and macroautophagy are the top 3 GO enrichments. Additionally, calcium ion transmembrane transport and regulation of NIK/NF-kappaB signaling are the significant GO enrichment results. **(D)** There are seven significant GO enrichment results in 26 upregulated OCRGs in organ failures (OFs). The upregulated genes are enriched in macroautophagy organelle fusion, autophagosome maturation, cytosolic calcium ion transport, negative regulation of neuron apoptotic process, antigen processing and presentation, and negative regulation of cellular component organization. **(E)** There are shared and exclusive pathways in four types of diseases in upregulated OCRGs. Venn diagram shows that there were five significant pathways shared by acute inflammations (AIs), metabolic diseases (MDs), autoimmune diseases (ADs), and organ failures (OFs). One pathway vesicle organization was shared by AIs and MDs. Mitochondrial fission pathway was shared by AIs and ADs. Macroautophagy pathway was shared by MDs and OFs. Organelle fusion was shared by MDs, ADs, and OFs. **(F)** The exclusive upregulated pathways in AIs, MDs, ADs, and OFs were 11, five, 17, and five, respectively.

**Figure 5:**
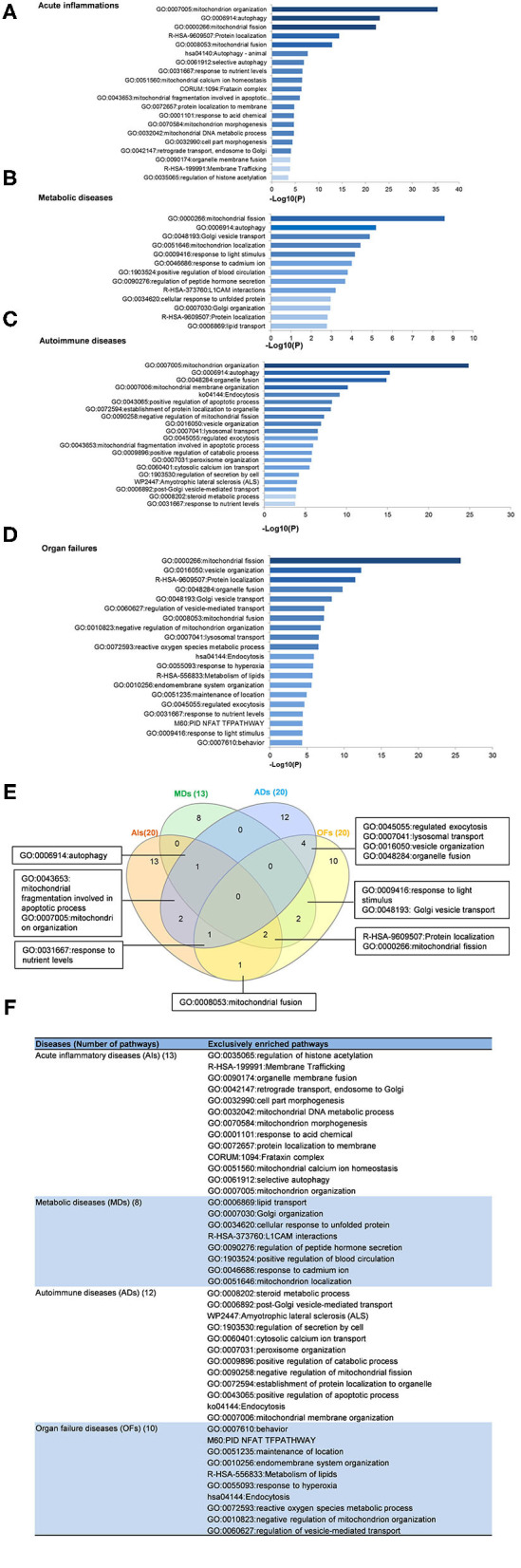
Enrichment analysis results show that several pathways of downregulated OCRGs were involved in acute inflammations (AIs), metabolic diseases (MDs), autoimmune diseases (ADs), and organ failures (OFs). Gene Ontology-based enrichment of significant downregulated OCRGs in different types of diseases was analyzed by using Metascape software (http://metascape.org/gp/index.html#/main/step1; PMID: 30944313). **(A)** In the 20 significant GO enrichment results of 58 downregulated OCRGs in acute inflammations (AIs), the top 3 of downregulated OCRGs are enriched in mitochondrion organization, autophagy, and mitochondrial fission. Mitochondrial calcium ion homeostasis is downregulated in acute inflammation diseases. **(B)** There are 13 significant GO enrichment results in 34 downregulated OCRGs in metabolic disease (MDs). Mitochondrial fission, autophagy, and Golgi vesicle transport are the top 3 downregulated signaling that enriched downregulated OCRGs. Additionally, lipid transport is suppressed. **(C)** In the 20 significant GO enrichment results of 67 downregulated OCRGs in autoimmune diseases (ADs), regulation of mitochondrion organization, organelle fusion, and autophagy are the top 3 downregulated enrichment signaling. Cytosolic calcium ion transport is suppressed, while calcium ion transmembrane transport is active in ADs (Figure 5). That is, calcium transporting between membrane-bounded organelles is active. **(D)** The 20 significant GO enrichment results of 99 downregulated OCRGs in organ failures (OFs). Top 3 GO enrichments of OCRGs are mitochondrial fission, vesicle organization, and protein localization. Reactive oxygen species metabolic process and metabolism of lipids were suppressed in organ failure diseases. **(E)** There are shared and exclusive pathways in four types of diseases in downregulated OCRGs. Venn diagram shows 13 significant pathways shared by acute inflammations (AIs), metabolic diseases (MDs), autoimmune diseases (ADs), and organ failures (OFs). Mitochondrial fragmentation involved in apoptotic process and mitochondrion organization pathways are downregulated in AIs and ADs. Autophagy signaling is decreased in AIs, MDs, and ADs. Mitochondrial fusion pathway is shared by AIs and OFs. Response to nutrient levels is downregulated in AIs, ADs, and OFs. Four pathways regulated exocytosis, lysosomal transport, vesicle organization, and organelle fusion and are downregulated in ADs and OFs. Response to light stimulus and Golgi vesicle transport are downregulated in MDs and OFs. Downregulation of R-HSA-9609507:Protein localization and mitochondrial fission are shared by AIs, MDs, and OFs. **(F)** The exclusive downregulated pathways in AIs, MDs, ADs, and OFs are 13, eight, 12 and 10, respectively.

To determine the functions of upregulated OCRGs, we performed MPAs. As shown in Figures 4A–D, AIs upregulated the top pathways including vacuole organization, lysosomal transport, neutrophil degranulation, regulation of endocytosis, positive regulation of peptidase activity, protein transfer within lipid bilayer, MT fission, vesicle organization, response to acid chemical, lysosomal organization, and receptor-mediated endocytosis. MDs upregulated the top pathways including MT organization, organelle fusion, macroautophagy, vesicle organization, response to anesthesia, myeloid leukocyte activation, protein localization, positive regulation of transmembrane transport, and endosomal transport. ADs upregulated the top pathways including MT fission, protein localization, membrane fusion, organelle fusion, neurodegeneration with brain iron accumulation (NBIA) subtype pathway, insertion of tail-anchored proteins, calcium ion transmembrane transport, vesicle fusion, Fragile X syndrome, MT localization, response to inorganic substance, regulation of NIK/NF-kB signaling, bile acid metabolic process, cholesterol metabolism, lung fibrosis, and protein targeting. OFs upregulated the top pathways including macroautophagy, organelle fusion, autophagosome maturation, cytosolic calcium ion transport, negative regulation of neuron apoptotic process, antigen processing and presentation, and negative regulation of cellular component organization. In Figures 5A–D, AIs downregulated the top 10 pathways including mitochondrion organization, autophagy, MT fission, protein localization, MT fusion, autophagy–animal, selective autophagy, response to nutrient levels, MT calcium ion homeostasis, and frataxin complex. MDs downregulated the top pathways including MT fission, autophagy, Golgi vesicle transport, mitochondrion localization, response to light stimulus, response to cadmium ion, positive regulation of blood circulation, regulation of peptide hormone secretion, L1CAM interactions, and cellular response to unfolded protein. ADs downregulated the top pathways including mitochondrion organization, autophagy, organelle fusion, MT membrane organization, endocytosis, positive regulation of apoptotic process, establishment of protein localization to organelle, negative regulation of MT fission, vesicle organization, and lysosomal transport. OFs downregulated the top pathways including MT fission, vesicle organization, protein localization, organelle fusion, Golgi vesicle transport, regulation of vesicle-mediated transport, MT fusion, negative regulation of mitochondrion organization, lysosomal transport, ROS metabolic process, endocytosis, and response to hyperoxia.

A Venn diagram was used to analyze the overlapping pathways of upregulated and downregulated OCRGs among these four major types of diseases. As shown in Figure 4E, for upregulated OCRGs, vesicle organization was shared by AIs and MDs; MT fission was shared by AIs and ADs; macroautophagy was shared by MDs and OFs; organelle fusion was shared by MDs, ADs, and OFs. Figure 4F lists the exclusively enriched upregulated pathways in these four diseases. In Figure 5E, for downregulated OCRGs, MT fragmentation involved in the apoptotic process and mitochondrion organization was shared by AIs and ADs; autophagy signaling was shared by AIs, MDs, and ADs; MT fusion was shared by AIs and OFs; response to nutrient levels was shared by AIs, ADs, and OFs; regulated exocytosis, lysosomal transport, vesicle organization, and organelle fusion were shared by ADs and OFs; response to light stimulus and Golgi vesicle transport were shared by MDs and OFs; protein localization and MT fission were shared by AIs, MDs, and OFs. Figure 5F lists the exclusively enriched downregulated pathways in these four diseases. These results have demonstrated that (1) the majority of signal pathways for upregulated OCRGs are the major disease group-specific; (2) upregulated pathway organelle fusion is shared by three disease groups such as MDs, ADs, and OFs; (3) upregulated MT fission is shared by AIs and ADs; (4) upregulated vesicle organization is shared by AIs and MDs; (5) upregulated protein localization is shared by MDs and ADs; and (6) more downregulated pathways are shared than upregulated pathways among diseases.

### Decreased Autophagosome–Lysosome Fusion Is Required for Viral Replications, Which Classify This Decreased Group as the Viral Replication-Suppressed Organelle Crosstalk Regulators

It has been reported that using single organelle multispectral flow cytometry identified altered energy metabolism, changes in MT size, and MT membrane potential in viral infected cells (81). We hypothesized that organelle crosstalk is modulated in cells infected by viruses. We collected 38 microarray datasets for comparison with seven groups of virus-infected cells including MERS-CoV infected human microvascular ECs (82) (0, 12, 24, 36, and 48 h post infection), SARS-CoV-infected human airway epithelium cells (83) (0, 12, 24, 36, 48, 60, 72, 84, and 96 h post infection), human influenza virus H1N1 infected human airway epithelium cells (83) (0, 6, 12, 18, 24, 36, and 48 h post infection), avian influenza virus A H7N9-infected Calu-3 human lung epithelium cells (84) (3, 7, 12, and 24 h post infection), avian influenza virus H7N7 infected Calu-3 human lung epithelium cells (84) (3, 7, 12, and 24 h post infection), avian influenza virus H5N1-infected Calu-3 human lung epithelium cells (84) (3, 7, 12, and 24 h post infection), human influenza virus H3N2-infected Calu-3 human lung epithelium cells (84) (3, 7, 12, and 24 h post infection), and H9N2-infected HUVECs (24 h). As shown in Table 3, our results indicated that (1) MERS-CoV infection in human microvascular ECs gradually modulated OCRG expressions in 12, 24, 36, and 48 h post infection with the peaks at 24 and 36 h (upregulated 41 and downregulated 44 OCRGs at 24 h and upregulated 39 and downregulated 46 OCRGs, respectively); (2) SARS-CoV infection slightly modulated OCRG expressions in human airway epithelial cells in 0, 24, 36, 48, 60, 72, 84, and 96 h post infection with the peak at 60 h post infection (upregulated 15 OCRGs and downregulated zero OCRG); (3) human influenza virus H1N1 infection significantly modulated OCRG expressions in human airway epithelial cells in 0, 6, 12, 18, 24, 36, and 48 h post infection with the peaks at 18, 24, 36, and 48 h post infection (upregulated 20, 22, 27, and 18 OCRGs and downregulated 21, 29, 30, and 16 OCRGs, respectively); (4) infections of avian influenza virus strains H7N9, H7N7, and H5N1 in human lung Calu-3 epithelium cells and strain H9N2 in HUVECs significantly modulated OCRG expressions at 24 h post infection with upregulation of 30, 38, 40, and 27 OCRGs and downregulation of 70, 109, 92, and 137 OCRGs, respectively; and (5) infection of human influenza virus strains H3N2 in human lung Calu-3 epithelium cells significantly modulated OCRG expressions at 12 and 24 h post infection with upregulation of 12 and 19 OCRGs and downregulation of 16 and 42 OCRGs, respectively.

**Figure 6:**
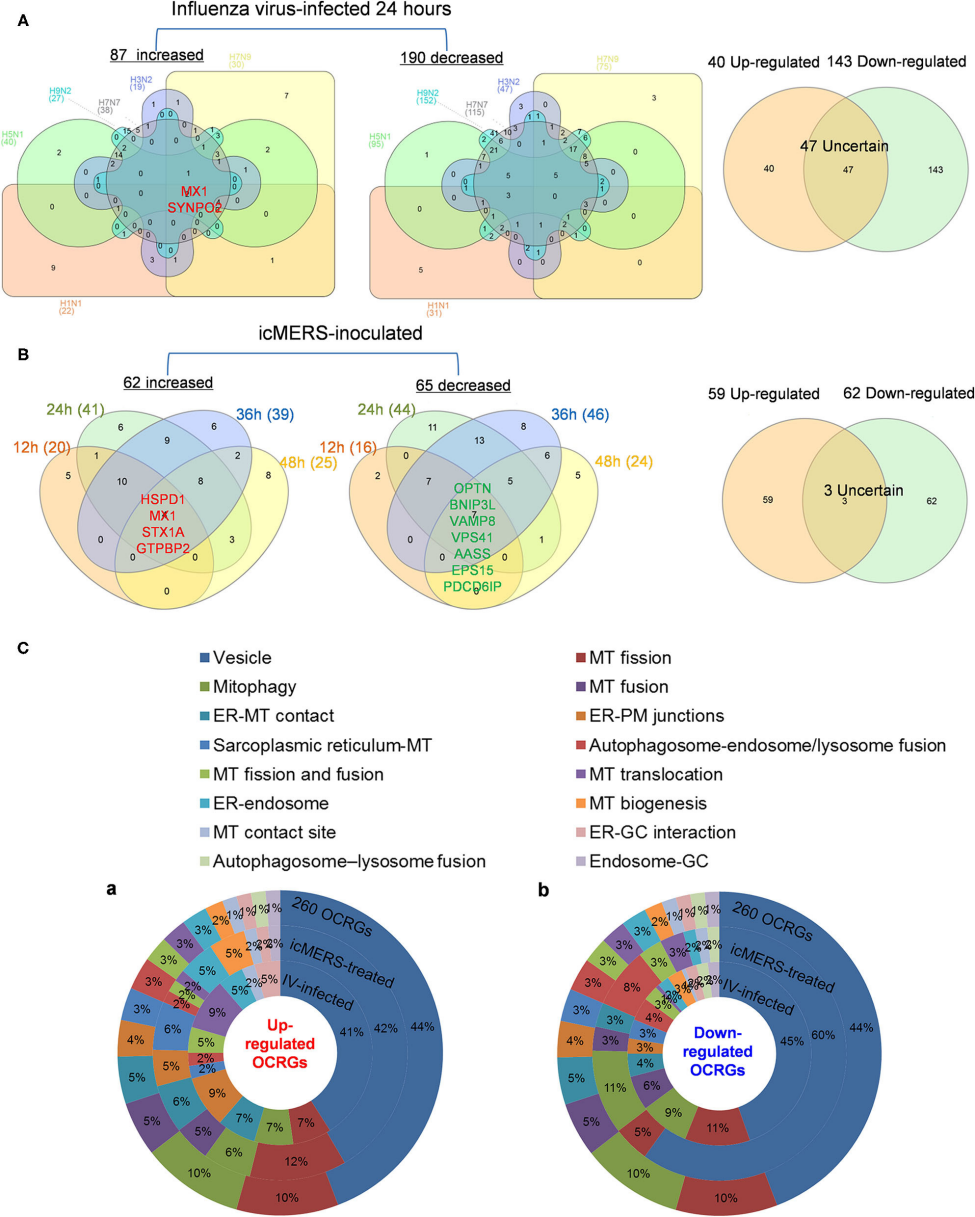
More OCRGs were significantly expressed in influenza virus- and icMERS-inoculated cells. **(A)** In H1N1-infected human microvascular endothelial cells, H7N9-, H7N7-, H5N1-, and H3N2-infected Calu-3 cells and H9N2-infected human umbilical vein endothelial cells, MX1 and SYNPO2 were the common upregulated OCRGs shared by these six types of flu virus-treated cells; no common downregulated genes were shared by these six flu virus-treated cells. When the overlapped 47 genes were removed, 40 genes are upregulated and 143 genes are downregulated in these five influenza virus-infected cells. **(B)** In time course, MERS coronavirus-inoculated human microvascular endothelial cells, HSPD1, MX1, STX1A, and GTPBP2 were the common upregulated OCRGs; and OPTN, BNIP3L, VAMP8, VPS41, AASS, EPS15, and PDCD6IP were the common downregulated OCRGs. When the overlapped three genes were removed, 59 genes are upregulated and 62 genes are downregulated in MERS coronavirus-inoculated cells. **(C)** Donut chart shows the ratio of classification of upregulated OCRGs **(A)** and downregulated OCRGs **(B)** in virus-treated cells. Classifications of upregulated OCRGs in icMERS- and influenza virus-inoculated cells are 15 and 12, respectively. Classifications of downregulated OCRGs in icMERS- and influenza virus-inoculated cells are 11 and 16, respectively.

Infections of avian influenza virus strains at 24 h significantly modulated OCRG expressions. We used a Venn diagram and MPA to examine avian influenza virus modulation of OCRGs. The result showed that 87 OCRGs were upregulated and 190 OCRGs were downregulated in influenza virus strains H1N1-, H7N9-, H7N7-, H5N1-, H3N2-, and H9N2-infected cells. MX1 and SYNPO2 were the commonly upregulated OCRGs shared by these six strains of influenza virus-infected cells; there were no commonly downregulated OCRGs by these six influenza virus-infected cells. Besides the overlapped 47 OCRGs, 40 OCRGs were upregulated (Supplementary Table 5A) and were from 12 functional groups except MT fusion, MT biogenesis, autophagosome–lysosome fusion, and endosome–Golgi complex (GC) groups; 143 OCRGs were downregulated and were from all the 16 functional groups (Figures 6A,C). The significant pathways for upregulated OCRGs included protein localization, mitochondrion organization, membrane fusion, organelle fusion, regulation of calcium ion transmembrane transporter activity, regulation of viral process, endomembrane system organization, metabolism of steroids, regulation of organelle assembly, and leukocyte chemotaxis. The top 10 pathways for downregulated OCRGs included mitochondrion organization, autophagy, MT fusion, MT transport, negative regulation of mitochondrion organization, lysosomal transport, organelle localization, endosomal transport, endocytosis, and protein localization (Supplementary Figures 3A,B).

Since MERS-CoV infection in human microvascular ECs induced significant modulation of OCRGs, we further used a Venn diagram and MPA to examine MERS-CoV modulation of OCRGs. As shown in Figure 6B, MERS-CoV infection resulted in upregulation of 62 OCRGs and downregulation of 65 OCRGs. The majority of upregulated OCRGs in 12, 24, 36, and 48 h post infections were shared in at least two time points; four OCRGs were shared in all four time points such as HSPD1, MX1, STX1A, and GTPBP2. Seven downregulated OCRGs such as OPTN, BNIP3L, VAMP8, VPS41, AASS, EPS15, and PDCD6IP were shared in all four time points. Besides the overlapped three genes, the upregulated 59 OCRGs were from 15 functional groups, except autophagosome–lysosome fusion; and the downregulated 62 OCRGs were from 11 functional groups, except ER–PM junctions, sarcoplasmic reticulum–MT, MT biogenesis, ER–GC interaction, and endosome–GC groups (Figure 6C). As shown in Supplementary Table 5B, downregulation of five including VAMP8, VPS41, ATG14, STX17, and RILP out of nine regulators were in autophagosome–endosome/lysosome fusion; and downregulation of one out of four regulators, TIRAP, was in the autophagosome–lysosome fusion group. The top 10 pathways for upregulated OCRGs included mitochondrion organization, synaptic vesicle budding from presynaptic endocytic zone membrane, regulation of mitochondrion organization, autophagy, protein localization, response to unfolded protein, cargo recognition for clathrin-mediated endocytosis, divalent metal ion transport, MT DNA metabolic process, and tissue remodeling. The top 10 pathways for downregulated OCRGs included macroautophagy, organelle fusion, autophagosome maturation, protein localization, vacuolar transport, regulation of MT fission, membrane trafficking, autophagosome membrane docking, endocytosis, and MT transport. The Venn diagram indicated that MT transport, endocytosis, regulated exocytosis, and autophagosome maturation pathways were commonly downregulated in influenza virus- and MERS infectious clone (icMERS)-inoculated cells (Supplementary Figures 3C,D). Taken together, our results have demonstrated that (1) virus infection in ECs and lung epithelial cells significantly modulate OCRGs in all the functional groups; (2) decreased one group autophagosome–lysosome fusion and four signaling pathways including MT transport, endocytosis, regulated exocytosis, and autophagosome maturation are the significant organelle crosstalk features of viral infections. The significant modulation of OCRGs by MERS-CoV in human microvascular ECs may be the underlying mechanism for much higher (41–50%) acute kidney injuries caused by MERS-CoV than that of SARS-CoV (6.7%) and COVID-19 (3%) (85). These results suggest that increased organelle crosstalk in all 15 functional groups (except autophagosome–lysosome fusion) but decreased lysosome degradation are required for viral replication; and significant modulation of organelle crosstalk in human microvascular ECs in coronavirus family infection represented by MERS-CoV may be the important underlying mechanism for COVID-19 (caused by SARS-CoV 2)-induced cardiovascular complications (86, 87).

### Organelle Crosstalk Regulators Upregulated by Pro-atherogenic Damage-Associated Molecular Patterns in Endothelial Cells Are Classified Endothelial Cell-Activation/Inflammation-Promoting Organelle Crosstalk Regulator Groups

Our previous reports showed that sterile inflammatory stimuli can cause intracellular organelle stress that occurs not only in cancer cells but also in vascular ECs (37). We reported that lysolipids are capable of transdifferentiating human aortic ECs (HAECs) into innate immune cells, including induction of potent DAMP receptors, such as CD36 molecule (14). CD36 pathways are activated by several distinct ligands, which converged on these pathways and results in inflammatory responses and endothelial dysfunction. CD36 pathway may be an underlying cause of CVDs and cerebrovascular diseases (88). Oxidized lipids upregulated by CD36 can modulate endothelial properties and may contribute to atherogenesis (89). However, how DAMP receptor signaling modulates expression of organelle crosstalking in ECs remains poorly understood. We examined the expression changes of OCRGs in ECs under stimulations of various pathogen-associated molecular patterns (PAMPs)/DAMPs (90–92). As shown in Table 3, influenza virus infection in HUVECs upregulated 27 OCRGs and downregulated 137 OCRGs. Kaposi sarcoma-associated herpes virus infection in human dermal ECs upregulated six OCRGs and downregulated 10 OCRGs, suggesting that acute inflammatory virus infection has higher OCRG expression modulation than chronic tumorigenic virus infection. Lipopolysaccharide (LPS) stimulation of human lung microvascular ECs (HLMECs) for 4, 8, and 24 h upregulated 8, 17, and 9 OCRGs, respectively, and downregulated 7, 15, and 14 OCRGs, respectively. LPS stimulation of mouse aortic ECs (MAECs) for 4 h upregulated three OCRGs and downregulated five OCRGs, which were similar to those found in HLMECs. It has been reported that Notch1 signaling is involved in regulating MT biogenesis (93). On the other hand, organelle crosstalking also regulates Notch1 signaling. For example, the endocytic trafficking of Notch receptor leads to either transportation to lysosomes for degradation via multi-vesicular bodies (MVBs) and late endosomes or recycling back to the plasma membrane for ligand binding activation (94). However, how modulation of Notch signaling regulates organelle crosstalking in ECs remains poorly characterized. Inhibition of endothelial NOTCH1 (inflammation promoting) signaling with siRNA in the absence or presence of inflammatory cytokine interleukin-1β (IL-1β) stimulation for 24 h in HUVECs resulted in no modulation or one OCRG downregulation (with IL-1β) (95). However, another report showed that reduction of NOTCH1 expression in HAECs by siRNA, in the absence of stimulations with inflammatory lipids or cytokines, increased inflammatory molecules and binding of monocytes (96). In the datasets of this paper, we found that inhibition of NOTCH1 expression with siRNA in HAECs upregulated three OCRGs and downregulated six OCRGs. In addition, interferon-α (IFNα), IFNβ and IFNγ treatment of HUVECs led to upregulation of 7, 11, and 6 OCRGs, respectively, but no downregulation of OCRGs. Of note, oscillatory shear vs. laminar shear (fibrosa), oscillatory shear vs. laminar shear (ventricularis) (97), and MAECs from atherogenic apolipoprotein E (ApoE)-deficient mice vs. MAECs from wild-type control mice had no modulation of OCRG expressions potentially due to a long-term chronic adaptation process in response to shear stress or hyperlipidemia. Pro-atherogenic DAMP stimuli oxidized low-density lipoprotein (oxLDL) (98) and proinflammatory oxidized 1-palmitoyl-2-arachidonoyl-sn-glycerol-3-phosphatidylcholine (oxPAPC) stimulation of MAECs presumably via transient receptor potential ankyrin 1 (TRPA1) (99) upregulated three, zero, and seven OCRGs and downregulated five, six, and seven OCRGs (Table 4).

**Figure 7:**
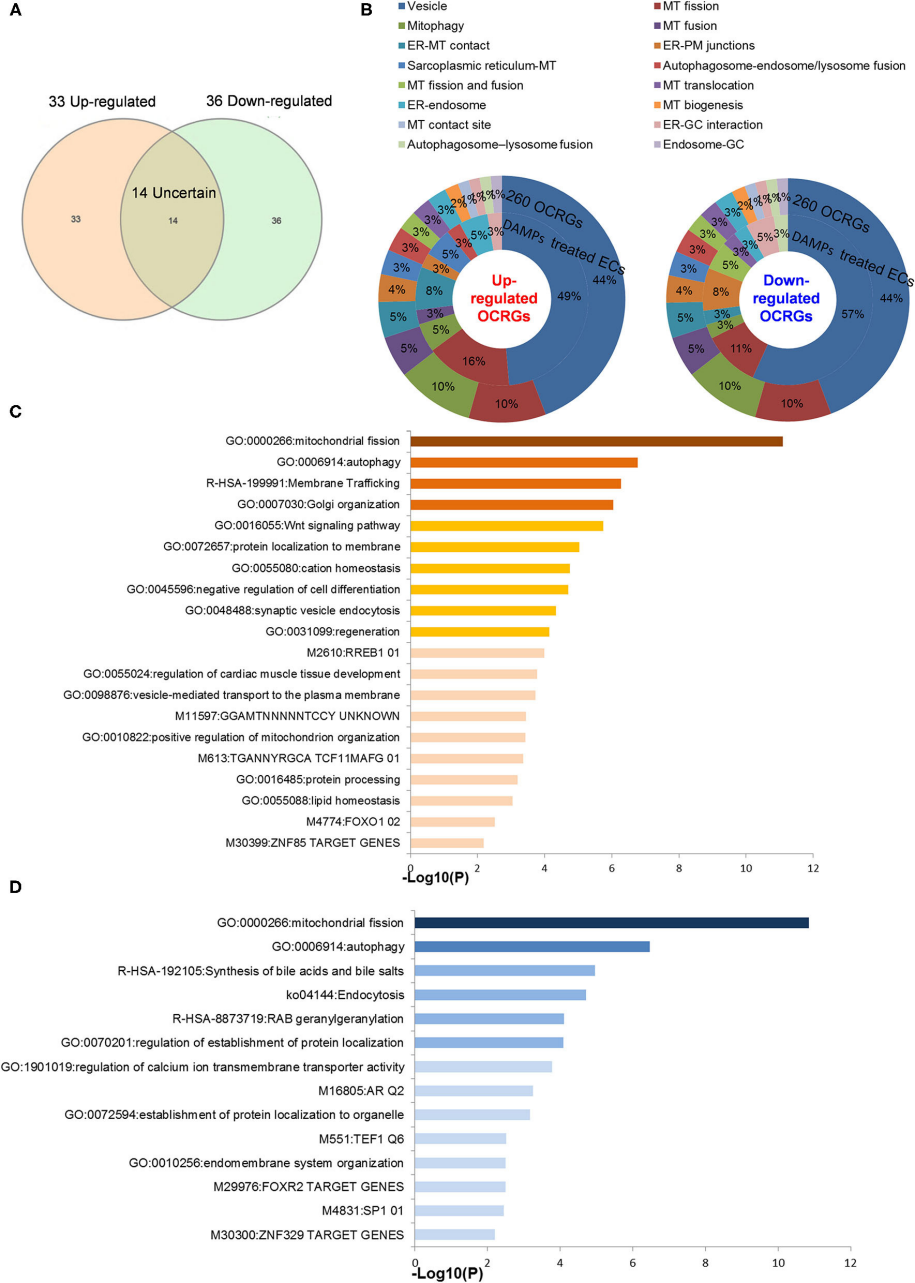
Classifications of OCRGs differentially expressed in endothelial cells treated with pro-atherogenic DAMPs such as oxLDL, LPS, oxPAPC, and IFNs treated. **(A)** When the overlapped genes in all upregulated OCRGs and in all downregulated OCRGs were removed, 33 genes are upregulated and 36 genes are downregulated exclusively in inflammatory factor-treated endothelial cells. **(B)** Donut chart shows the ratio of classification of upregulated OCRGs (left) and downregulated OCRGs (right) in pro-atherogenic DAMP-treated endothelial cells. There are 10 classifications in 33 upregulated and 36 downregulated OCRGs, respectively. There are no MT fission and fusion, MT translocation, MT biogenesis, MT contact site, autophagosome–lysosome fusion, and endosome–GC OCRGs in upregulated genes and not MT fusion, sarcoplasmic reticulum–MT, autophagosome–endosome/lysosome fusion, MT biogenesis, MT contact site, and endosome–GC OCRGs in downregulated genes. **(C)** There are 20 significant GO enrichment results in 33 upregulated OCRGs of pro-atherogenic DAMP-treated endothelial cells. Top 3 GO enrichments of OCRGs are membrane trafficking, autophagy, and mitochondrial fission. Wnt signaling pathway is upregulated, and this change suggests inflammatory factors may be via Wnt signaling pathway, promoting inflammation induced by OCRGs. **(D)** There are 14 significant GO enrichment results in 36 downregulated OCRGs in inflammatory factor-treated endothelial cells. Calcium ion transmembrane transporter activity signaling is downregulated, and this change suggests that inflammatory factors can change calcium ion transmembrane transporter activity via OCRGs in endothelial cells. Enrichment analysis was performed by using Metascape software (http://metascape.org/gp/index.html#/main/step1; PMID: 30944313).

As shown in Figures 7A,B (Supplementary Table 6 showed the detailed differentially expressed OCRGs), the 33 upregulated OCRGs in ECs included 10 out of 16 functional groups of OCRGs (except MT fission and fusion, MT translocation, MT biogenesis, MT contact site, autophagosome–lysosome fusion, and endosome–GC groups): (1) six MT fission regulators, DNM2, INF2, TMEM135, COX10, DDHD1, and LRRK2; (2) two mitophagy regulators, SQSTM1 (also vesicle group) and OPTN; (3) two ER–MT contact and sarcoplasmic reticulum–MT regulators, HSPA9 and ITPR1; (4) one ER–PM junction regulator, JPH2; (5) one MT fusion regulator, BAK1 (also ER–MT contact group); (6) one autophagosome–endosome/lysosome fusion regulator, STX17; and (7) the rest of the 17 vesicle regulators including BMP2, CD24, CSF2, POU2F2, ATP23, CLTC, P4HA2, VPS26A, ATP11A, CCZ1, EIF4ENIF1, RPS6KC1, GTPBP2, NOV, PICALM, PNPLA2, and RUNX1. The 36 downregulated OCRGs in ECs also included 10 out of 16 functional groups of OCRGs (except MT fusion, sarcoplasmic reticulum–MT, autophagosome–endosome/lysosome fusion, MT biogenesis, MT contact site, and endosome–GC groups). The upregulated top 5 pathways involved in DAMP-stimulated ECs included MT fission, autophagy, membrane trafficking, Golgi organization, and WNT pathway (Figure 7C). The downregulated top 5 pathways included MT fission, autophagy, synthesis of bile acids and bile salts, endocytosis, and RAB geranylgeranylation (Figure 7D). By comparison, 20 OCRGs were upregulated and were classified into five groups (Supplementary Figures 5A,B); the top pathways involved in upregulating OCRGs in LPS-stimulated ECs included MT fission, endosomal transport, receptor internalization, cytokine signaling in immune system, and ZNF410 TARGET GENES (Supplementary Figure 5C), latter four top pathways of which were different from those in DAMP-stimulated upregulation of OCRGs in ECs. The top pathways involved in downregulating OCRGs in LPS-stimulated ECs included MT fission, regulation of calcium ion transmembrane transporter activity, negative regulation of transferase activity, receptor metabolic process, and establishment of organelle localization, also latter four top pathways of which were different from those in DAMP-stimulated upregulation of OCRGs in ECs (Supplementary Figure 5D). Taken together, our results have demonstrated that (1) acute inflammatory (influenza) virus infection has higher OCRG modulation than in chronic tumorigenic (herpes) virus infection in ECs; (2) EC activation stimuli such as TLR4 stimulation by LPS and oxLDL result in modulation of OCRGs in HAECs and MAECs; (3) modulation of OCRGs by inhibition of NOTCH1 in HAEC is associated with increased inflammatory status and increased atherosclerosis in endothelial NOTCH1 heterozygous mice; (4) proinflammatory cytokines type 1 IFN (α, β, and probably ω, κ, and ε) signaling and type II IFN (γ) (100) modulated OCRG expression in ECs; and (5) oscillatory shear vs. laminar shear vs. laminar shear and ApoE KO MAECs have no modulation of OCRGs, potentially due to adaptation of ApoE KO MAECs and interplays of multiple pathways bone morphogenetic protein (BMP)-transforming growth factor-β (TGFβ), Wingless and Int-1 (WNT), Notch membrane receptors (NOTCH), hypoxia inducible factor-α (HIF1α a transcription factor), Twist family basic helix-loop-helix (BHLH) transcription factor 1 (TWIST1), and a subset of homeobox genes (HOX family) (101).

### The Expressions of Organelle Crosstalk Regulators Are Modulated in Regulatory T Cells in Comparison With Those of CD4^+^CD25^–^ T Effector Controls; Upregulated Organelle Crosstalk Regulators Are More Than Downregulated Organelle Crosstalk Regulators in Regulatory T Cells

Recent reports showed that the MT membrane potential and the proliferation inhibition function of CD4^+^Foxp3^+^ Treg from patients with myasthenia gravis are enhanced by autophagy inducing agent rapamycin and suppressed by phosphoinositide 3-kinase (PI3 kinase) and autophagy inhibitor 3-methyladenine (3-MA) (102) and that during autoimmune conditions, Treg function alterations associate with MT oxidative stress, dysfunctional mitophagy, and enhanced DNA damage and cell death (103, 104). We hypothesized that the expressions of OCRGs are modulated in CD4^+^CD25^high^FOXP3^+^ Treg in comparison with those of CD4^+^CD25^−^ T effector cell controls. As shown in Table 5, Treg from eight different tissues including lymph nodes (LNs), visceral adipose tissue, brown adipose tissue (cold), brown adipose tissue (warm), spleen from mice with skeletal muscle injury for 4 days, spleen from mice with skeletal muscle injury for 14 days, and spleen from male and female mice had no downregulated OCRGs. In addition, Treg from five tissues such as warm spleen, skeletal muscle from mice with skeletal muscle injury for 4 days, skeletal muscle from mice with skeletal muscle injury for 14 days, intestine, and peripheral blood had one, four, two, one, and one downregulated OCRGs, respectively. Moreover, Treg in spleen from female mice had no upregulated OCRGs; and Treg from the rest of the 12 groups had one to 12 upregulated OCRGs (Supplementary Table 7 listed, in detail, the significant expressed OCRGs in Treg compared with conventional T cells). To examine a hypothesis that the expressions of OCRGs are required for the suppressive functions of Treg, we determined this hypothesis with Treg signature gene deficient microarray datasets. The 15 microarrays of Treg regulator deficiency datasets were collected to analyze the expression changes of OCRGs (Table 6). In the deficiency datasets of Treg signature genes GATA binding protein 3 (Gata3), histone deacetylase 9 (Hdac9), and peroxisome proliferator activated receptor gamma (Pparg) in LNs, there were no significant expression changes of OCRGs. There were seven significantly upregulated OCRGs and no downregulated OCRGs in Treg signature transcription factor B-cell lymphoma 6 (Bcl6) knockout Treg from spleen and LN. There were six significantly upregulated OCRGs and one downregulated OCRG in Foxo1 knockout Treg from the thymus, spleen, and LNs. In Dicer 1 (ribonuclease III, microRNA-maturation enzyme) knockout CD4^+^ T cells, there were five upregulated OCRGs and one downregulated OCRG. In tripartite motif containing 28 [E3 small ubiquitin-like modifier (SUMO)-protein ligase Trim28] knockout Treg, there were three upregulated OCRGs and five downregulated OCRGs. In B lymphocyte-induced maturation protein 1 (Blimp1) deficiency Treg, there were three upregulated OCRGs and six downregulated OCRGs.

**Figure 8:**
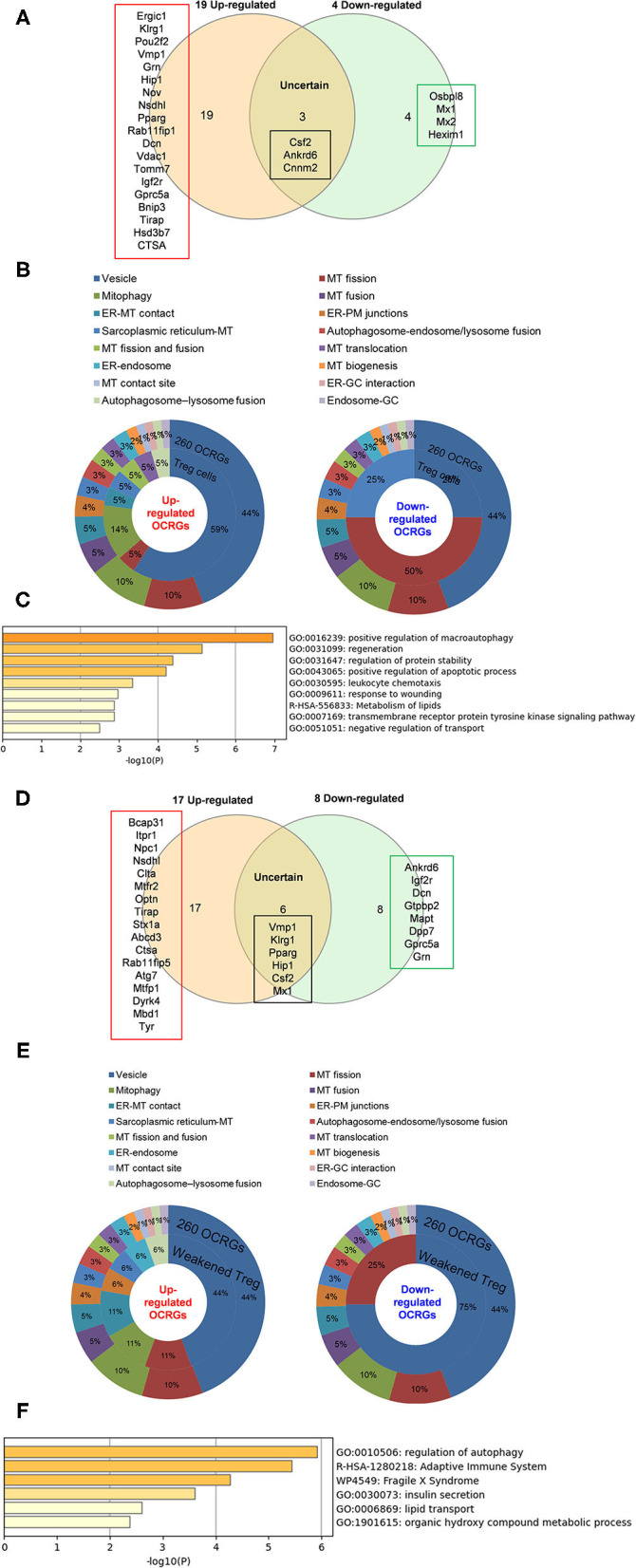
Less classification of OCRGs differentially expressed in regulatory T cells and weakened Treg cells. **(A)** When the overlapped three genes in all upregulated OCRGs and in all downregulated OCRGs were removed, 19 genes are upregulated and four genes are downregulated exclusively in regulatory T cells. **(B)** Donut chart shows the ratio of classification of upregulated OCRGs (left) and downregulated OCRGs (right) in regulatory T cells. There are eight classifications in 19 upregulated OCRGs and three classifications in four downregulated OCRGs. **(C)** Enrichment analysis results of 19 upregulated genes show signaling pathways of positive regulation of macroautophagy, regeneration, regulation of protein stability, positive regulation of apoptotic process, leukocyte chemotaxis, response to wounding, metabolism of lipids, transmembrane receptor protein tyrosine kinase signaling pathway, and negative regulation of transport were upregulated. **(D)** When the overlapped five genes in all upregulated genes and in all downregulated genes were removed, 17 genes are upregulated and eight genes are downregulated exclusively in regulatory T-cell signature gene deficiency (weakened regulatory T cells). **(E)** Donut chart shows the ratio of classification of upregulated OCRGs (left) and downregulated OCRGs (right) in weakened regulatory T cells. There are eight classifications (vesicle, MT fission, Mitophagy, ER–MT contact, ER–PM junctions, sarcoplasmic reticulum–MT, ER–endosome, and autophagosome–lysosome fusion) in 17 upregulated OCRGs and two classifications (vesicle and MT fission) in eight downregulated OCRGs, respectively. **(F)** Enrichment analysis results of 17 upregulated genes show signaling pathways of regulation of autophagy, adaptive immune system, Fragile X syndrome, insulin secretion, lipid transport, and organic hydroxy compound metabolic process.

As shown in Figure 8A, when combining all the upregulated and downregulated OCRGs in the Treg, besides the overlapping genes, four OCRGs downregulated in Treg included sarcoplasmic reticulum–mitochondria contact regulator OSBP-related protein 8 (Osbpl8) (105), MT fission regulators MX1 and MX2, and one vesicle regulator Hexim1 (Supplementary Table 8). There were no significant pathways of the upregulated OCRGs from MPA. The 19 upregulated OCRGs included one autophagosome–lysosome fusion regulator Tirap, one mitophagy regulator Vmp1, one MT fission gene Dcn, one MT translocation Tomm7, Vdac1 with more function (mitophagy, ER–MT contact, sarcoplasmic reticulum–MT), MT fission and fusion, mitophagy regulator Bnip3, and the other 13 vesicle regulators (Supplementary Table 8 and Figure 8B). The top signaling pathways of these upregulated OCRGs from the MPA included positive regulation of macroautophagy, regeneration, regulation of protein stability, positive regulation of apoptotic process, and leukocyte chemotaxis (Figure 8C). These results have demonstrated for the first time that Treg from various tissues and mice with injured skeletal muscle have upregulation of OCRGs compared with those of CD4^+^CD25^−^ T effector controls; and Treg slightly upregulate a few OCRGs in Treg from visceral adipose tissue, skeletal muscle, and intestine. The results of weakened Treg cells from 11 microarrays (Treg signature gene knockout datasets) showed that the upregulated OCRGs were more than downregulated OCRGs (Table 6 and Supplementary Table 9 list in detail differentially expressed OCRGs). As shown in Figure 8D, the 17 upregulated OCRGs included one autophagosome–lysosome fusion gene Tirap, one ER–endosome contact gene Npc1, two ER–MT contact genes Bcap31 and Itpr1 (also sarcoplasmic reticulum–MT group), one ER–PM junctions Stx1a, two mitophagy genes Optn and Atg7, two MT fission genes Mtfr2 and Mtfp1, and the other eight vesicle regulators (Nsdhl, Clta, Abcd3, Ctsa, Rab11fip5, Dyrk4, Mbd1, and Tyr). The eight downregulated OCRGs included two MT fission regulators Dcn and Mapt and six vesicle regulators (Ankrd6, Igf2r, Gtpbp2, Dpp7, Gprc5a, and Grn) (Supplementary Table 9 and Figure 8E). There were no significant pathways in the downregulated OCRGs from MPA. The signaling pathways of these upregulated OCRGs included regulation of autophagy, adaptive immune system, Fragile X syndrome, insulin secretion lipid transport, and organic hydroxy compound metabolic process (Figure 8F). Taken together, our results have demonstrated that (1) the expression of OCRGs are modulated in CD4^+^FOXP3^+^ Treg and Treg cells with signature genes deficiencies; and (2) upregulated OCRGs in Treg are more than downregulated OCRGs. In weakened Treg when Treg signature genes are deficient, upregulated OCRGs are more than downregulated OCRGs; and (3) positive regulation of macroautophagy, regeneration, regulation of protein stability, positive regulation of apoptotic process, and leukocyte chemotaxis signaling are upregulated in Treg; and regulation of autophagy, adaptive immune system, Fragile X syndrome, insulin secretion, lipid transport, and organic hydroxy compound metabolic process are upregulated in weakened Treg.

### Toll-Like Receptors, Reactive Oxygen Species Regulator Nuclear Factor Erythroid 2-Related Factor 2, and Inflammasome-Activated Caspase-1 Regulate the Expressions of Organelle Crosstalk Regulators

**Figure 9:**
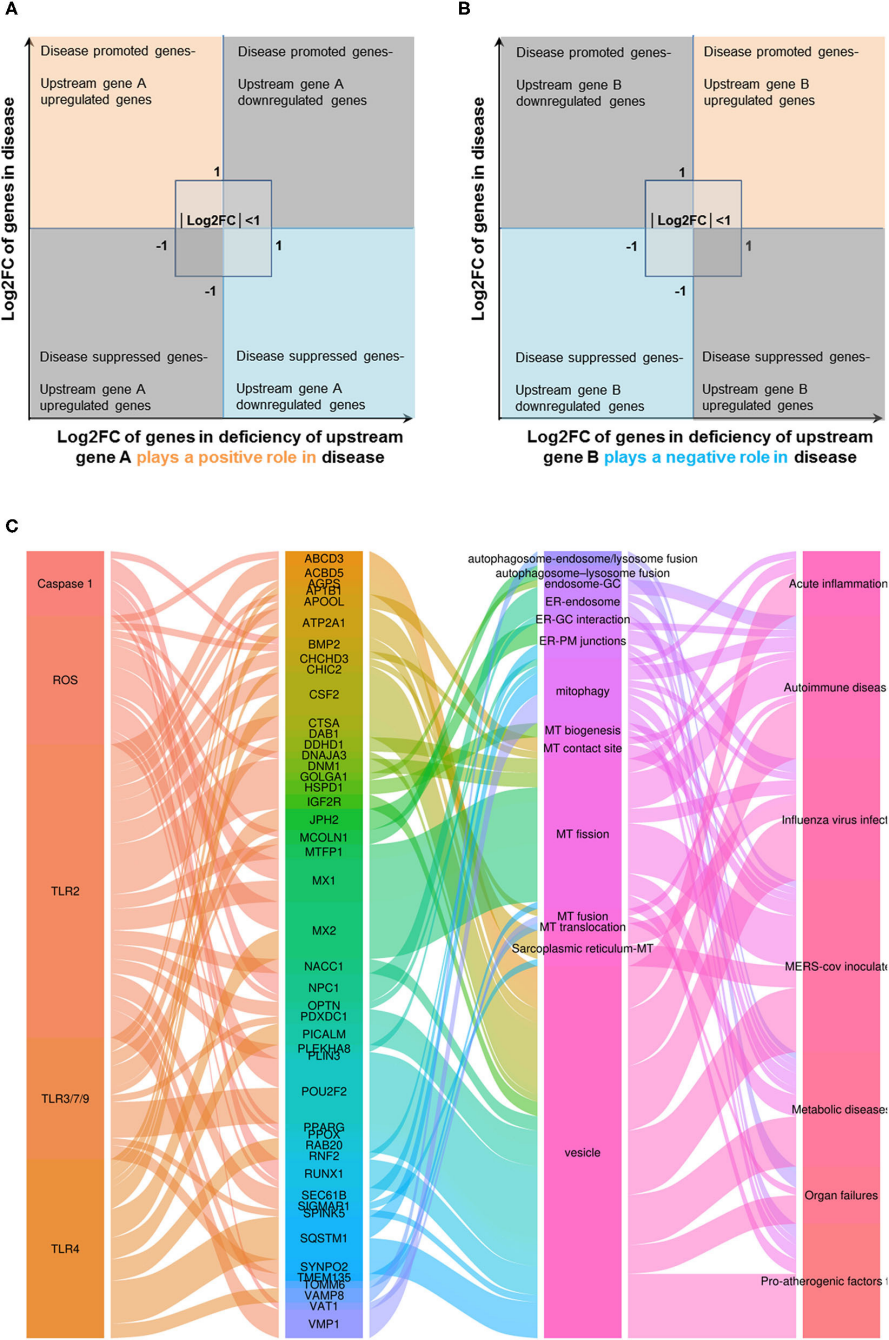
Three types of regulators (TLRs, caspase-1, and ROS regulators) upregulated a total of 46 OCRGs in different diseases, and virus- and pro-atherogenic factor-treated cells. **(A)** Two models were used to explore the mechanisms of expression changes of OCRGs in the upstream gene-deficient microarrays. When the upstream gene A plays a positive role (promote disease) in disease (or in virus, pro-atherogenic DAMP-treated cells), OCRGs upregulated in the disease will be downregulated in deficiency of upstream gene A (upper left box). OCRGs downregulated in the disease will be upregulated in deficiency of upstream gene A (bottom right box). **(B)** When the upstream gene B plays a negative role (suppress disease) in disease (or in virus, pro-atherogenic DAMP-treated cells), OCRGs upregulated in the disease will be upregulated in deficiency of upstream gene B (upper right box). OCRGs downregulated in the disease will be downregulated in deficiency of upstream gene B (bottom left box). **(C)** Alluvial plot shows the regulated 46 OCRGs by TLR2, TLR4, TLR3/7/9, caspase-1, and ROS regulator Nrf2; and these 46 OCRGs classified 14 groups of all 16 groups (except for MT fission and fusion, and ER–MT contact OCRGs), and vesicle, MT fission, and mitophagy are the top 3 groups. TLR2 regulated 24 OCRGs and caspase-1 regulated five OCRGs. Twelve OCRGs (i.e., CSF2, MX1, ABCD3, ATP2A1, APOOL, PDXDC1, MX2, CSF2, POU2F2, MCOLN1, NPC1, RUNX1, and BMP2) were regulated by two upstream regulators; other OCRGs were regulated by one upstream regulator. The regulated OCRGs were more in autoimmune diseases and virus-infected cells than in other diseases. Alluvial plot was plotted by http://www.bioinformatics.com.cn, an online platform for data analysis and visualization. The detailed relation between OCRGs and upstream regulators is listed in Supplementary Table 10.

In order to determine the mechanisms underlying the expression changes of OCRGs in diseases, virus-infected cells, and pro-atherogenic DAMP-treated ECs, we examined expressions of OCRGs in nine microarrays including six TLRs (106), one ROS regulator Nrf2 (23), and two caspase-1-deficient microarrays. Caspase-1 is activated in the DAMP/PAMP-sensing protein complexes termed inflammasomes (3, 11, 16, 36, 39, 90, 106–110) (Tables 7–9). The results showed that TLR2, TLR4, and TLR3/7/9 deficiencies upregulated 29, seven, and nine OCRGs and downregulated 37, 12, and 12 OCRGs, respectively (Supplementary Figure 6). In addition, ROS negative regulator Nrf2 deficiency upregulated 14 and downregulated seven OCRGs. Moreover, caspase-1 deficiency upregulated seven and downregulated five OCRGs. Then we proposed two models to explore the mechanisms underlying the expression changes of OCRGs regulated by DAMP/PAMP sensing TLRs and caspase-1/inflammasomes (Figures 9A,B). A Venn diagram was used to identify the regulated OCRGs in diseases, virus infections, and cells stimulated by pro-atherogenic DAMPs (Supplementary Figures 7–11). As shown in Figure 9C and Supplementary Table 10, TLRs, caspase-1, and ROS regulators play significant roles in upregulating a total of 46 OCRGs (17.7%) from 14 groups (except for MT fission and fusion and ER–MT contact OCRGs) in different diseases, virus, and pro-atherogenic factor-treated cells. Of note, the signaling pathways in upregulating 12 OCRGs (i.e., CSF2, MX1, ABCD3, ATP2A1, APOOL, PDXDC1, MX2, CSF2, POU2F2, MCOLN1, NPC1, RUNX1, and BMP2) are shared by two groups of upstream regulators. These findings suggest that these 12 OCRGs may play important roles in pathophysiologies upregulated by DAMP/PAMP signaling.

### Organelle Crosstalk Regulators Are Significantly Modulated in Cancer; Upregulations of Organelle Crosstalk Regulators in Liver Hepatocellular Carcinoma and Lung Squamous Cell Carcinoma Are Correlated With Immune Infiltrates; Mitochondrial Import Machine Is Upregulated in Cancers and Is Associated With Immune Infiltrates

It has been reported that mitophagy pathways are intricately linked to the metabolic rewiring of cancer cells to support the high bioenergetic demand of the tumors (111); autophagy, acting as a cancer-suppressive function, is inclined to hinder metastasis by selectively downregulating critical transcription factors of the epithelial–mesenchymal transition (EMT) in the early phases (112). The molecular mechanisms underlying fusion, with either lysosomes or plasma membrane, are key determinants to maintain cell homeostasis upon stressing stimuli. The accumulation of undigested substrates leads to cancer and other diseases such as lysosomal storage disorders and age-related neurodegenerative diseases (113). We hypothesized that carcinogenesis modulates the expressions of OCRGs. To examine this hypothesis, we collected the expression data of 260 OCRGs in 28 cancer datasets in the NIH-NCI TCGA (https://www.cancer.gov/about-nci/organization/ccg/research/structural-genomics/tcga/using-tcga)/GTEx database (GTEx Portal, https://www.gtexportal.org/home/) from EGPIA2 (http://gepia2.cancer-pku.cn/#index). As shown in Table 10, all the 28 cancers from the gastrointestinal, respiratory, brain, genitourinary, hematopoietic, and digestive systems significantly modulated the expressions of OCRGs. The upregulated OCRGs range from one OCRG in lung adenocarcinoma (LUAD) to 166 OCRGs in thymoma; and downregulated OCRGs range from three in pancreatic adenocarcinoma (PAAD) to 65 in testicular germ cell tumor.

**Figure 10:**
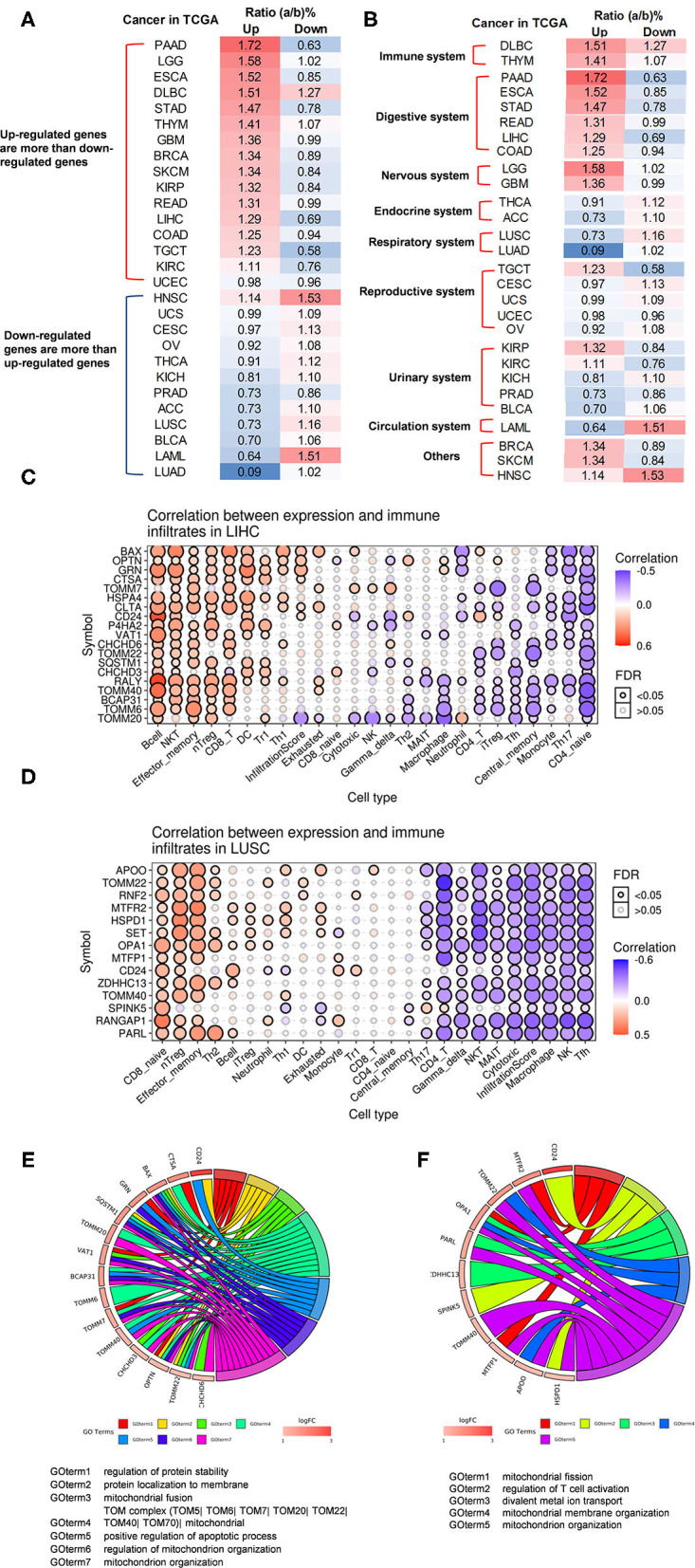
The ratio of upregulated and downregulated OCRGs has different dominance in tumors of different systems, and differentially expressed OCRGs are correlated with immune infiltrates in liver hepatocellular carcinoma (LIHC) and lung squamous cell carcinoma (LUSC). **(A)** Tumors are listed according to the ratio of upregulated and downregulated OCRGs. The results show that in pancreatic adenocarcinoma (PAAD), brain lower-grade glioma (LCC), and esophageal carcinoma (ESCA), these 15 cancers [from PAAD to uterine corpus endometrial carcinoma (UCEC)] upregulated OCRGs more than the downregulated OCRGs; in lung adenocarcinoma (LUAD), adrenocortical carcinoma (ACC), and acute myeloid leukemia (LAML), these 13 cancers (from HNSC to LUAD) downregulated OCRGs more than the upregulated genes. In order to rule out the influence of the overall expression profile on our interest OCRGs, we take the ratio of number of OCRGs and number of total differently expressed genes as the comparison criteria for the number of upregulated and downregulated genes. Result show that 16 cancers have upregulated OCRG percentage >1% and that 12 cancers have upregulated OCRGs percentages <1%. **(B)** Expression of OCRGs in tumors was roughly classified according to the system. The numbers of upregulated OCRGs are more than downregulated genes in tumors of immune system, digestive system, and nervous system. The numbers of downregulated OCRGs are more than upregulated OCRGs in tumors of the endocrine system, respiratory system, and circulation system and most tumors of the reproductive system and urinary system. In breast invasive carcinoma (BRCA) and skin cutaneous melanoma (SKCM), the numbers of upregulated OCRGs are more than those of downregulated OCRGs. In head and neck squamous cell carcinoma (HNSC), the numbers of upregulated OCRGs are less than those of downregulated OCRGs. **(C)** Almost 19 upregulated OCRGs in LIHC are positively correlated with B cell, NKT, effector memory, nTreg, CD8 T, DC, Tr1, and Th1 infiltration but negatively correlated with macrophage, neutrophil, CD4 T, iTreg, central memory, monocyte, Th17, and CD4 naïve cell infiltration. Expressions of BAX, OPTN, GRN, CTSA, CLTA, and CD24 are positively correlated with infiltration score, and expression of TOMM20 is negatively correlated with infiltration score. **(D)** Fourteen upregulated OCRGs in LUSC are positively correlated with CD8 T, nTreg, and effector-memory cell infiltration and negatively correlated with CD4 T, gamma-delta, NKT, MAIT, cytotoxic macrophage, NK, and Tfh cell infiltration. Expressions of all 14 OCRGs are negatively correlated with infiltration score. Correlations between expression of upregulated OCRGs and immune infiltrates were analyzed in LIHC and LUSC in Gene Set Cancer Analysis (GSCA) database (http://bioinfo.life.hust.edu.cn/GSCA/#/immune). **(E)** Fifteen out 19 upregulated OCRGs in LIHC enriched in seven significant pathways including regulation of protein stability, protein localization to membrane, mitochondrial fusion, TOM mitochondrial, positive regulation of apoptotic process, regulation of mitochondrion organization, and mitochondrion organization. More OCRGs enriched in TOM complex mitochondrial and mitochondrion organization (GOterm4 and GOterm7). **(F)** Eleven out of 14 OCRGs in LUSC enriched in five significant pathways including mitochondrial fission, regulation of T cell activation, divalent metal ion transport, mitochondrial membrane organization, and mitochondrion organization. More OCRGs (TOMM40, APOO, PARL, HSPD1, MTFP1, TOMM22, OPA1, and MTFR2) enriched mitochondrion organization pathway (GOterm5). GO (Gene Ontology) chord was generated by http://www.bioinformatics.com.cn, an online platform for data analysis and visualization.

To identify the common features of OCRG modulation in cancers, we calculated the percentage (a/b%) of upregulated OCRGs (a) over total upregulated expressed genes and (b) in the cancer microarrays, and the ratios of downregulated OCRGs over total downregulated genes. We noticed that 16 cancers had upregulated OCRG percentage >1% and 12 cancers had upregulated OCRGs percentages <1%. Cancers were roughly classified into eight systems to explore the expression changes of OCRGs. The numbers of upregulated OCRGs were more than those of downregulated genes in tumors of the immune system, digestive system, and nervous system. The numbers of downregulated OCRGs were more than those of the upregulated OCRGs in tumors of the endocrine system, respiratory system, and circulation system and most tumors of the reproductive system and urinary system. In breast invasive carcinoma (BRCA) and skin cutaneous melanoma (SKCM), the numbers of upregulated OCRGs were more than those of downregulated OCRGs. In head and neck squamous cell carcinoma (HNSC), the numbers of upregulated OCRGs were less than those of downregulated OCRGs (Figures 10A,B). We then examined a hypothesis that upregulations of OCRGs are correlated with increased immune cell infiltration. To examine this hypothesis, GSCA database (http://bioinfo.life.hust.edu.cn/GSCA/#/immune) (76) was used to analyze the correlation of upregulated OCRGs with immune infiltrates. Liver hepatocellular carcinoma (LIHC) and lung squamous cell carcinoma (LUSC) were studied for the details as an example. The result in Figure 10C shows that almost 19 upregulated OCRGs in LIHC were positively correlated with B cells, natural killer (NK) cells, effector-memory T cells, naturally occurring Treg (nTreg), CD8 T cells, dendritic cells (DCs), type 1 Treg (Tr1), and type 1 T helper cells (Th1) infiltration but negatively correlated with macrophages, neutrophils, CD4 T cells, inducible Treg (iTreg), central memory T cells, monocytes, Th17, and CD4 naïve cell infiltrations. The expressions of BAX, OPTN, GRN, CTSA, CLTA, and CD24 were positively correlated with infiltration scores; and the expressions of TOMM20 were negatively correlated with infiltration scores. Almost 14 upregulated OCRGs in LUSC were positively correlated with CD8 T cells, nTreg, and effector-memory T-cell infiltrations and were negatively correlated with CD4 T cells, gamma-delta T cells, NK cells, mucosal-associated invariant T cells (MAIT), cytotoxic macrophages, NK cells, and follicular T helper (Tfh) cells infiltrations. The expressions of all 14 OCRGs were negatively correlated with immune cell infiltration scores (Figure 10D). Therefore, our findings have demonstrated that the expression modulations of OCRGs can predict the degrees of tumor immune infiltrations and serve as potential therapeutic targets. The GO (Gene Ontology) chord analysis showed that more upregulated OCRGs in LIHC and LUSC were enriched in mitochondrion organization pathway than other pathways (Figures 10E,F). In fact, the upregulated OCRGs in most tumors can be enriched in mitochondrion organization pathways (Supplementary Figure 12).

We further examined five digestive cancers such as PAAD, stomach adenocarcinoma (STAD), rectal adenocarcinoma (READ), LIHC, and colon adenocarcinoma (COAD) among the cancer groups with >1% OCRGs and performed the Venn diagram analysis on upregulated OCRGs of these five digestive cancers. As shown in Supplementary Figure 13A, the results showed that PAAD had 80 specific OCRGs (54.8%) out of 146 upregulated OCRGs. In contrast, the other cancers had much lower specific OCRGs. For example, STAD, READ, LIHC, and COAD had three (5.6%), one (2.8%), two (11.1%), and zero (0%) specific OCRGs, respectively. Of note, one OCRG shared by all five cancer groups was translocase of outer MT membrane 40 (TOMM40). We also found that TOMM40 was upregulated in 14 out of 33 types of cancers (42.4%) (Supplementary Figure 13B). Our results are well-correlated with previous reports that TOMM40 (TOM40) and significant numbers of MT protein import machineries overexpressed in cancers such as the TOM complex (TOM20, TOM40, TOM7, and TOM70), TIM23 complex, TIM22 complex, small Tim chaperons and Mia40, and Tim 44 (111, 114). To elucidate the functional aspects of TOMM40, we searched the PPI network database in the STRING database (https://string-db.org/). As shown in Supplementary Figure 13C, the top 10 proteins functionally interacted with TOMM40 including translocase of outer MT membrane (TOM) complex members TOMM7, TOMM20, TOMM70A, and TOMM22; translocase of inner MT membrane (TIM) complex members TIMM50, TIMM44, TIMM13, and TIMM10; sorting and assembly machinery complex (SAM) member SAMM50; and CYC1 (cytochrome C1, complex III subunit 4). As shown in Supplementary Figures 13D,E, the most relative top 10 genes to TOMM40 were localized in two types of protein complexes, the translocase of outer MT membrane complexes (TOMs) and translocase of inner MT membrane complexes (TIMs), and were mainly enriched in protein targeting to mitochondria, protein transporter activity and MT protein complex in biology process, molecular function and cell component (115, 116). The enriched pathways of these 11 genes were MT protein import, Pink/Parkin-mediated mitophagy, and Ub-specific processing proteases (117). We further determined whether increased expressions of TOMM40 and 10 interaction proteins in cancers lead to better prognosis by analyzing the overall survival map of hazard ratio (HR) and disease-free survival map [relapse-free survival (RFS)] of TOMM40 and 10 interaction proteins. The result showed that overall survival of patients in most cancers were positively related to these 11 genes (red box in Supplementary Figure 13F); and RFS of patients in most cancers was positively related to upregulation of these 11 genes (red box in Supplementary Figure 13G; the blue box was the negative correlation between the expression of genes and RFS). It has been reported that MT protein transport system is modulated in cancers (118). A previous report showed that TOMM70 acts as receptor of the MT antiviral-signaling protein (MAVS) and thereby participates in the corresponding system of innate immunity against viral infections (119). It remains unknown whether this innate immune function of TOMM70 is correlated with the better prognosis of cancers upregulating TOMM40 complexes. Relations between abundance of tumor-infiltrating lymphocytes, immuno-inhibitors, immuno-stimulators, MHCs, and expression of TOMM40 were explored by using TISIDB database (http://cis.hku.hk/TISIDB/index.php) (75). The results indicated that the expressions of TOMM40 were positively correlated with abundance of act CD8, act CD4, CD56, and monocyte cells in most of the cancers and that immuno-inhibitor PVRL2; immuno-stimulators CD276, PVR, TNFRSF18, TNFRSF25, and TNFRSF4; and HLA-A, HLA-B, HLA-C, HLA-F, TAP1, TAP2, and TAPBP molecules were positively correlated with expression of TOMM40 in most of cancers (Supplementary Figure 14). We speculate that upregulation of TOMM40 may improve the prognosis of patients by promoting tumor immunity.

### Tumor Promoter Factor and Tumor Suppressor Significantly Modulate the Expressions of Organelle Crosstalk Regulators; Vesicle Regulators May Attenuate or Increase Tumorigenesis When Tumor Promoter Factor or Tumor Suppressor Is Deficient

We then hypothesized that oncogenes and tumor suppressors play important roles in regulating the expressions of OCRGs in cancer cells. To test this hypothesis, we collected a total of 19 microarrays from the deficiencies of oncogenes and tumor suppressors in NIH–NCBI–GEO dataset database (https://www.ncbi.nlm.nih.gov/gds/). Since NF-kB and inflammation have been linked to Kras mutations and cancer development, inhibitor of NF-kB kinase subunit b (IKK2) depletion in lung tumor cells significantly attenuates tumor proliferation and prolongs mouse survival (120). As shown in Table 11, IKK2 KO vs. wild-type control upregulated five OCRG and downregulated five OCRGs in mouse lung tumor lines. Four additional IKK and IKK2 knockout (KO) in mouse lung tumor nodules, mouse and human leukemia cells, and human MDA-MB-231 breast cancer epithelial cells did not significantly modulate OCRGs. Deficiencies of NF-kB subunit Rela in mouse lung carcinoma cells and human MDA-MD-231 cells upregulated one and five OCRGs and downregulated two OCRGs. In addition, the Janus kinases (JAKs)/signal transducer and activator of transcription proteins (STATs) signaling pathway provide important roles in contributing to oncogenesis in hematological malignancies and solid tumors (121). JAK2 knockdown (KD) in human acute myelogenous leukemia (AML) cell line, STAT1 KD in human T cell acute lymphocytic leukemia cells, Stat3 KD in mouse mammary tumor, and STAT3 KD in human urothelial cancer cells resulted in zero to two OCRG upregulation and one to five OCRG downregulations. Moreover, tumor suppressor and transcription factor Tp53 KO in mouse liver tumor (122), mouse mammary epithelial cells, oviductal cells, neural stem cells, small intestine, and neu primary tumor led to upregulation of zero up to 23 OCRGs and downregulation of zero up to 48 OCRGs, respectively. Of note, adenomatous polyposis coli (APC) is a multi-functional tumor suppressor gene (123); and a limited role for Tp53 is found in modulating early tumor progression induced by APC loss in mouse intestine (124). APC KO led to six OCRG upregulation and three OCRG downregulation compared with that of wild-type controls, respectively. Furthermore, phosphatidylinositol 3-kinase (PI3-kinase) and phosphatase and tensin homolog deleted on chromosome 10 (PTEN) are major positive and negative regulators of PI3 kinase pathway in regulating cell growth, survival, and proliferation and are two of the most frequently mutated proteins in human cancers (125). PTEN is a tumor suppressor (126); and PTEN KD and KO resulted in zero to seven OCRG upregulation and zero to four OCRG downregulation. In all the significantly modulated OCRGs, there were 13 upregulated and 16 downregulated OCRGs in the oncogene deficiency databases examined and 47 upregulated and 50 downregulated OCRGs in tumor suppressors deficiency databases (Supplementary Figures 15A,B, Supplementary Tables 11, 12 listed, in detail, the modulated OCRGs). These results have demonstrated that in tumor conditions, upregulated oncogenes positively increased the expressions of 16 upregulated OCRGs and 13 downregulated OCRGs. In contrast, tumor suppressors deficiencies upregulated 47 OCRGs in these datasets. Upregulated and downregulated OCRGs were classified into seven groups in oncogene deficiencies and were classified into 12 and 14 groups in tumor suppressors deficiencies (Supplementary Figure 15C).

Our data in Table 11 show that IKK2 depletion upregulated five OCRGs in lung tumor cells, which were well-correlated with the finding that IKK2 depletion attenuated tumor proliferation and prolonged mouse survival. We then tested a hypothesis that IKK2 depletion upregulated OCRGs may partially overlap with LUAD-downregulated OCRGs. As shown in Supplementary Figure 16A, the Venn diagram indicated that vesicle regulator perilipin 4 (PLIN4) was overlapped between the LUAD-downregulated OCRGs and the IKK2 KO-upregulated OCRGs. As shown in Supplementary Figure 16B, the expression profile analysis using cancer and normal gene expression database GEPIA (http://gepia.cancer-pku.cn/index.html) (127) showed that the expression of PLIN4 was decreased in 23 out of 33 tumor types compared with controls. Our data in Table 8 show that tumor suppressor Tp53 KO in liver tumors upregulated 23 OCRGs and downregulated 48 OCRGs, which were associated with induction of carcinomas in Tp53 KO mice (122, 126). Similarly, we then tested a hypothesis that Tp53 KO upregulated OCRGs may partially overlap with that liver tumor (LIHC)-upregulated OCRGs; and Tp53 KO downregulated OCRGs may partially overlap with liver tumor (LIHC)-downregulated OCRGs. As shown in Supplementary Figure 16C, the Venn diagram indicated that prolyl 4-hydroxylase subunit alpha 2 (P4HA2) was shared between the Tp53 KO upregulated OCRGs and the liver tumor (LIHC)-upregulated OCRGs; and alpha-aminoadipic semialdehyde synthase (AASS) was shared between the Tp53 KO downregulated OCRGs and the liver tumor (LIHC)-downregulated OCRGs. The cancer gene expression database analysis showed that the expressions of vesicle regulator P4HA2 were increased in seven out of 33 tumor types compared with controls (Supplementary Figure 16D); and the expression of vesicle regulator AASS in seven out of 33 tumor types was decreased compared with that of controls (Supplementary Figure 16E). Taken together, our results have demonstrated that *first*, tumor promoter IKK2 and tumor suppressor Tp53 significantly modulate the expressions of OCRGs; *second*, lung tumor LUAD-downregulated vesicle regulator PLIN4 may contribute to IKK2 KO-promoted tumor formation; *third*, liver tumor (LIHC)-upregulated vesicle regulator P4HA2 may increase Tp53 KO-promoted liver tumorigenesis; and *fourth*, LIHC-downregulated vesicle regulator AASS may contribute to Tp53 suppression of liver tumorigenesis.

## Discussion

Although alterations to these networks, such as impaired ER–mitochondria MCSs, have been linked to several diseases such as neurodegeneration (59, 60), CVD (61), diabetes (62), kidney diseases (63, 64), and cancers (65, 66), an important question remains poorly characterized how the expressions of organelle crosstalk regulator genes are modulated in various diseases. To examine whether the expressions of 260 OCRGs in 16 functional groups are modulated in 23 diseases and 28 tumors, we performed extensive -omics data mining analyses in a panoramic manner with the method that we pioneered in 2004 (23, 26, 29, 91, 107, 109, 128) and made a set of significant findings: (1) the ratios of upregulated vs. downregulated OCRGs are 1:2.8 in AIs, 1:1 in MDs, 1:1.1 in ADs, and 1:3.8 in OFs; and AIs and OFs upregulate less OCRG groups (three to seven out of 16) than metabolic and ADs (11 to 13 out of 16); (2) sepsis and trauma-upregulated OCRG groups including vesicle, MT fission, and mitophagy but not other groups of OCRGs; thus, the three groups of OCRGs are classified as the acute crisis-handling OCRGs; and similarly, sepsis and trauma plus OFs-upregulated seven OCRG groups including vesicle, MT fission, mitophagy, sarcoplasmic reticulum–MT, MT fusion, autophagosome–lysosome fusion, and autophagosome/endosome–lysosome fusion are classified as the cell failure-handling OCRGs; (3) the majority of upregulated pathways are disease group-specific; upregulated organelle fusion and macroautophagy are shared by MDs, ADs, and OFs; upregulated MT fission is shared by AIs, MDs, and ADs; upregulated vesicle organization is shared by AI and MDs; and upregulated protein localization is shared by MDs and ADs; (4) increased OCRG expressions in all the 15 functional groups except autophagosome–lysosome fusion but decreased autophagosome–lysosome fusion are required for viral replications, which classify this decreased group as the viral replication-suppressed OCRGs. (5) PAMPs from influenza virus and Kaposi sarcoma-associated herpes virus infections and pro-atherogenic DAMPs such as oxLDL, LPS, oxPAPC, and IFNs totally upregulated 33 OCRGs in ECs, which classify vesicle, MT fission, mitophagy, MT fusion, ER–MT contact, ER–PT junction, and autophagosome/endosome–lysosome fusion as the seven EC-activation/inflammation-promoting OCRG groups; (6) the expression of OCRGs is modulated in Treg from the LNs, spleen, peripheral blood, intestine, and brown adipose tissue in comparison with that of CD4^+^CD25^−^ T effector controls; (7) TLR2, TLR4, TLR3/7/9, caspase-1, and ROS regulator Nrf2 can upregulate 46 OCRGs from 14 groups out of all 16 groups (except for MT fission and fusion, and ER–MT contact OCRGs), suggesting that DAMP/PAMP-sensing systems play significant roles in modulating the expressions of OCRGs; (8) OCRG expressions are significantly modulated in all the 28 cancer datasets; upregulated OCRGs in LIHC and LUSC are correlated with immune cell infiltrations, suggesting that upregulation of certain OCRGs may promote immune cell infiltration; MT protein translocase TOMM40 and MT import machine are upregulated in cancers and are positively associated with better prognosis; and TOMM40 is correlated with some immune infiltration signature genes, suggesting that increase of protein transport into mitochondria may facilitate the immune cell infiltration; and (9) tumor promoter factor IKK2 and tumor suppressor Tp53 significantly modulate the expressions of OCRGs; lung tumor-downregulated vesicle regulator PLIN4 may contribute to IKK2 KO-attenuated tumor formation; liver tumor-upregulated vesicle regulator P4HA2 may increase Tp53 deficiency-promoted liver tumorigenesis; and liver tumor-downregulated vesicle regulator AASS may contribute to Tp53 suppression of liver tumorigenesis.

The original microarray experiments we analyzed used different cells, which prevented us from comparing the effects of various regulators in regulating the expressions of OCRGs in the same cell types, although our database mining method was not ideal. However, to fill in the important knowledge gaps, our approach was justified. Actually, this approach has been a common practice that we (23) and others (72, 129) often use in studying gene expression in non-ideal, heterogeneous PBMC populations in disease conditions vs. healthy conditions, and PBMCs are actually composed of many cell types, such as B cells (~15%), T cells (~70%), monocytes (~5%), and NK cells (~10%) (130).

**Figure 11:**
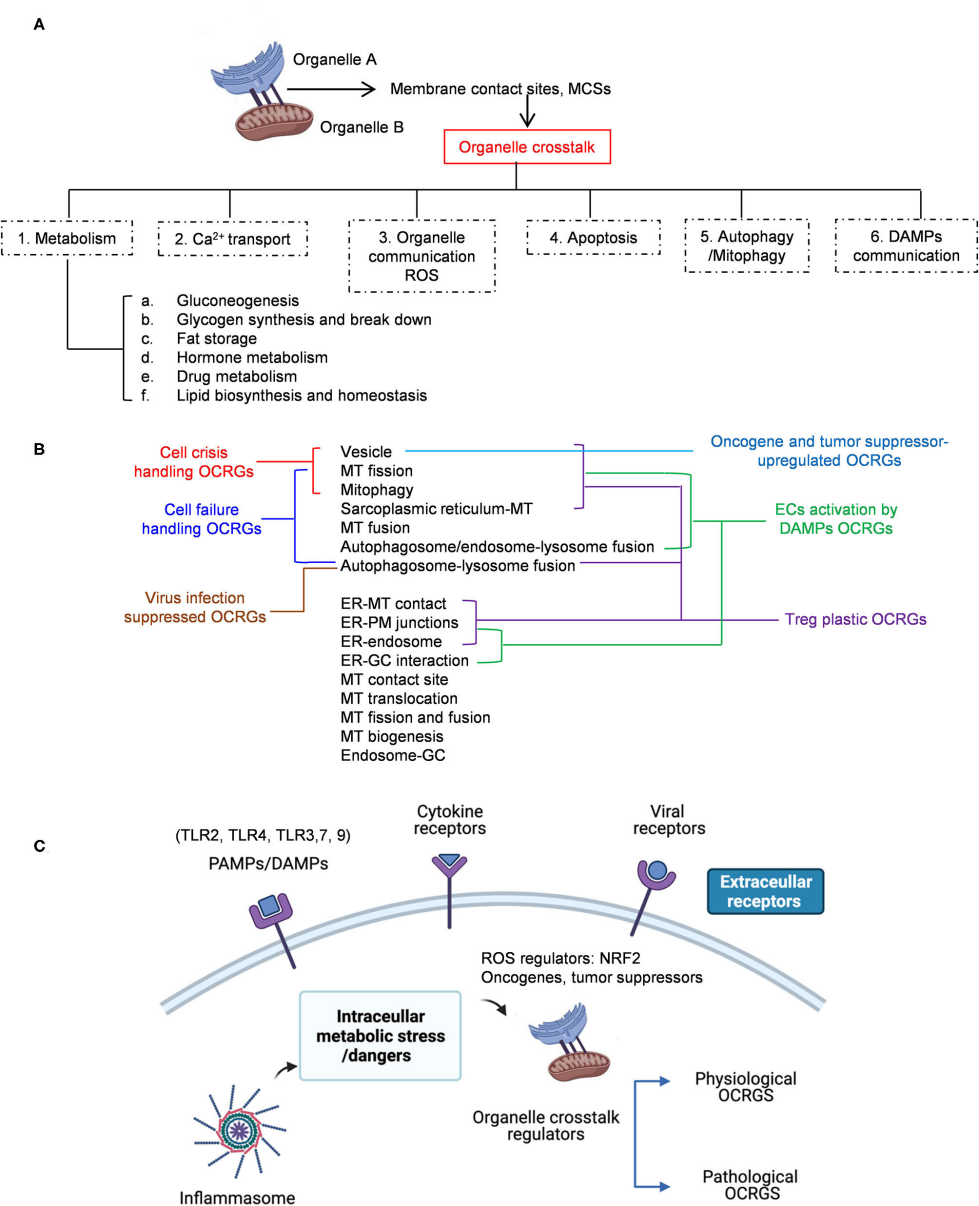
A novel working model shows the function, classification, and regulated mechanisms of OCRGs in diseases, and DAMP- and virus-treated cells. **(A)** The membrane contact sites between two organelles, at least between ER and mitochondria (Mito, MT), have been found to play several significant functions from previous reports and our study, such as in metabolism (including gluconeogenesis, glycogen synthesis and breakdown, fat storage, hormone metabolism, drug metabolism, lipid biosynthesis, and homeostasis), Ca^2+^ transport, organelle communication ROS, apoptosis, autophagy/mitophagy, and DAMP communication. **(B)** Thirteen of 16 functional groups of OCRGs are differently modulated in various diseases and cancers. Three groups of OCRGs such as vesicle, mitochondrial fission, and mitophagy were identified as the cell crisis-handling OCRGs (red line pointed). Seven groups of OCRGs including three cell crisis-handling OCRGs plus sarcoplasmic reticulum-mito, mitochondrial fusion, autophagosome–lysosome fusion, and autophagosome/endosome–lysosome fusion were identified as the cell failure-handling OCRGs (blue line pointed). Autophagosome–lysosome fusion OCRGs can be regarded as virus infection-suppressed OCRGs (brown line pointed). Eight classification groups (i.e., vesicle, MT fission, mitophagy, sarcoplasmic reticulum–MT, autophagosome–lysosome fusion, ER–MT contact, ER–PM junctions, and ER–endosome) are Treg plastic OCRGs (purple line pointed). Ten groups of ECs activation by DAMP OCRGs include seven Treg plastic OCRGs (except autophagosome–lysosome fusion) and add MT fusion, autophagosome/endosome–lysosome fusion, and ER–GC interaction OCRGs (green line pointed). Vesicle regulators can be regarded as oncogenes and tumor suppressor-promoted OCRGs (light blue line pointed). **(C)** Extracellular pathogen-associated molecular patterns (PAMPs)/damage-associated molecular patterns (DAMPs), cytokines, and viruses act via their receptors including TLRs on the cell membrane and intracellular locations (Nod-like receptors and inflammasomes) to activate OCRGs directly or indirectly by activating intracellular organelle dangers first and then OCRGs. And ROS regulator NRF2 oncogenes and tumor suppressors also regulate the expression of OCRGs in diseases and tumors. A new concept that the organelle crosstalk serves as both new DAMP sensing and communicating network and new cell crisis and cell failure-handling network to fulfill both physiological functions and pathological functions. *Created with BioRender.com.

To summarize our findings from a panoramic analysis on the expression changes of 260 OCRGs in 51 diseases and cancers presented here, we propose a novel working model to integrate these results, as follows, in Figure 11A: *first*, the MCSs between two organelles, at least between ER and mitochondria (Mito, MT), have been found to play several significant functions such as metabolism and Ca^2+^ transport as we reported (4); organelle communicating ROS as we recently reported (4, 38, 40–42, 131); apoptosis as we have reported (132, 133); and autophagy, mitophagy, and DAMP communication as we have reported (37). The metabolism regulatory functions between ER–MT contact sites include gluconeogenesis, glycogen synthesis and breakdown, fat storage, hormone metabolism, drug metabolism, lipid biosynthesis, and homeostasis (134); *second*, since our study analyzed the 260 OCRGs in 51 major diseases and cancers and many other master regulator deficient transcriptomic data, we have demonstrated for the first time that 16 functional groups of OCRGs are not equally modulated in various diseases and cancers. As shown in Figure 11B, we identified three groups of OCRGs such as vesicle, MT fission, and mitophagy as the cell crisis-handling OCRGs, at least in partial functions of the three groups; seven groups of OCRGs including three cell crisis-handling OCRGs plus sarcoplasmic reticulum–MT, MT fusion, autophagosome–lysosome fusion, and autophagosome/endosome–lysosome fusion as the cell failure-handling OCRGs; and autophagosome–lysosome fusion as virus-suppressed OCRGs. Previous reports showed that growing numbers of viruses are found to weaponize the ubiquitin modification system to suppress anti-viral type I IFN pathways (135). Similarly, we found that virus infections weaponize the suppression of autophagosome–lysosome fusion group potentially for virus replication. Since most viruses use endocytosis to enter the host cell, 11 clinically approved generic drugs are identified as potential candidates for repurposing as blockers of several potential routes for severe acute SARS-CoV-2 endocytosis (136), which needs to be further re-considered since we found that coronavirus and other virus infections in ECs and epithelial cells suppress endocytosis–lysosome pathways (Supplementary Figure 4); 10 groups of OCRGs activated by DAMPs in ECs including vesicle (137), MT fission, mitophagy, autophagosome/endosome–lysosome fusion, ER–MT contact, ER–PM junctions, sarcoplasmic reticulum–MT, MT fusion, ER–endosome, and ER–GC interaction. Treg plastic OCRGs, and oncogenes and tumor suppressors promoted vesicle regulators; and *third*, extracellular PAMPs/DAMPs, cytokines, and viruses act via their receptors including TLRs on the cell membrane and intracellular locations (Nod-like receptors and inflammasomes) (90) to activate OCRGs directly or indirectly by activating intracellular organelle dangers first and then OCRGs. Therefore, similar to our recently proposed new concept that ROS are an integrated system for sensing and alarming organelle metabolic homeostasis and dysfunction (38), here, we propose a new concept that the organelle crosstalk serves as both new DAMPs sensing and communicating network and new cell crisis and cell failure-handling network (37) to fulfill both physiological functions and pathological functions (Figure 11C).

One limitation of the current study is that due to the low throughput nature of verification techniques in every laboratory including ours, we could not verify every result we identified with the analyses of high-throughput data, which are similar to all the papers with RNA-Seq, single-cell RNA-Seq, and other -omics data. We acknowledge that carefully designed *in vitro* and *in vivo* experimental models will be needed in the future to verify regulator gene deficiency-upregulated OCRGs further and the underlying mechanisms we report here. Nevertheless, our findings provide novel insights on the roles of upregulated OCRGs in the pathogenesis of inflammatory diseases and cancers, novel pathways for the future therapeutic interventions for inflammations, sepsis, trauma, OFs, ADs, metabolic CVDs, and cancers.

## Data Availability Statement

The datasets generated for this study can be found in online repositories. The names of the repository/repositories and accession number(s) can be found in the article/Supplementary Material.

## Author Contributions

ML carried out the data gathering and data analysis and prepared the tables and figures. NW, KX, FS, EV, YS, RZ, JW, HS, WY, YL, YS, CD, LLiu, LLi, WH, JY, DP, JS, XJ, and HW aided with analysis of the data. XY supervised the experimental design, data analysis, and manuscript writing. All authors read and approved the final manuscript.

## Conflict of Interest

The authors declare that the research was conducted in the absence of any commercial or financial relationships that could be construed as a potential conflict of interest.
