# Supplementary material for: Organelle Crosstalk Regulators Are Regulated in Diseases, Tumors, and Regulatory T Cells: Novel Classification of Organelle Crosstalk Regulators
Source: Front Cardiovasc Med. 2021 Jul 22;8:713170. doi: 10.3389/fcvm.2021.713170 (PMC8339352; doi:10.3389/fcvm.2021.713170)
Supplement: Supplementary file 19 [file Presentation_1.pptx]

## Slide 1
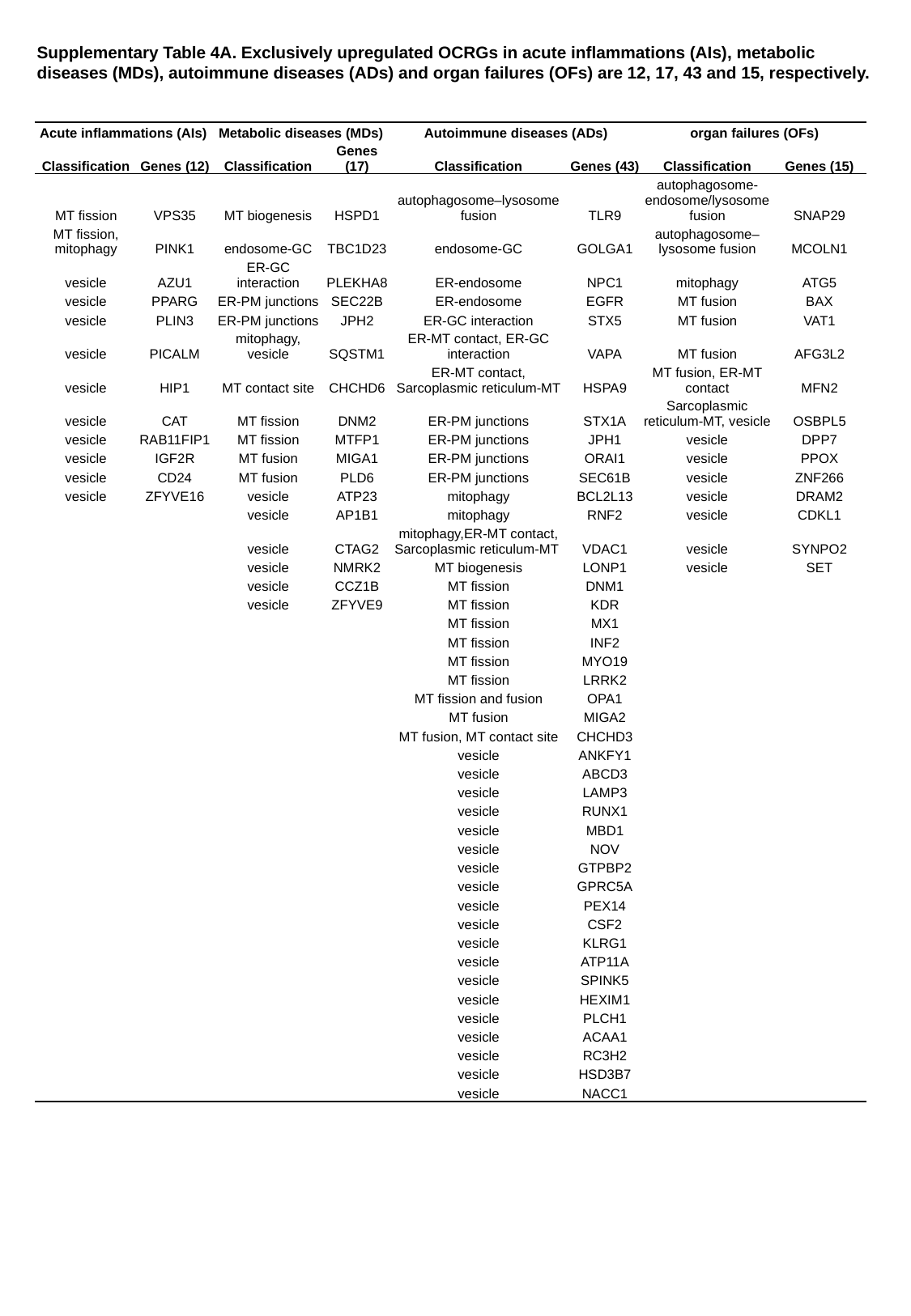

Supplementary Table 4A. Exclusively upregulated OCRGs in acute inflammations (AIs), metabolic diseases (MDs), autoimmune diseases (ADs) and organ failures (OFs) are 12, 17, 43 and 15, respectively.
| Acute inflammations (AIs) | | Metabolic diseases (MDs) | | Autoimmune diseases (ADs) | | organ failures (OFs) | |
| --- | --- | --- | --- | --- | --- | --- | --- |
| Classification | Genes (12) | Classification | Genes (17) | Classification | Genes (43) | Classification | Genes (15) |
| MT fission | VPS35 | MT biogenesis | HSPD1 | autophagosome–lysosome fusion | TLR9 | autophagosome-endosome/lysosome fusion | SNAP29 |
| MT fission, mitophagy | PINK1 | endosome-GC | TBC1D23 | endosome-GC | GOLGA1 | autophagosome–lysosome fusion | MCOLN1 |
| vesicle | AZU1 | ER-GC interaction | PLEKHA8 | ER-endosome | NPC1 | mitophagy | ATG5 |
| vesicle | PPARG | ER-PM junctions | SEC22B | ER-endosome | EGFR | MT fusion | BAX |
| vesicle | PLIN3 | ER-PM junctions | JPH2 | ER-GC interaction | STX5 | MT fusion | VAT1 |
| vesicle | PICALM | mitophagy, vesicle | SQSTM1 | ER-MT contact, ER-GC interaction | VAPA | MT fusion | AFG3L2 |
| vesicle | HIP1 | MT contact site | CHCHD6 | ER-MT contact, Sarcoplasmic reticulum-MT | HSPA9 | MT fusion, ER-MT contact | MFN2 |
| vesicle | CAT | MT fission | DNM2 | ER-PM junctions | STX1A | Sarcoplasmic reticulum-MT, vesicle | OSBPL5 |
| vesicle | RAB11FIP1 | MT fission | MTFP1 | ER-PM junctions | JPH1 | vesicle | DPP7 |
| vesicle | IGF2R | MT fusion | MIGA1 | ER-PM junctions | ORAI1 | vesicle | PPOX |
| vesicle | CD24 | MT fusion | PLD6 | ER-PM junctions | SEC61B | vesicle | ZNF266 |
| vesicle | ZFYVE16 | vesicle | ATP23 | mitophagy | BCL2L13 | vesicle | DRAM2 |
| | | vesicle | AP1B1 | mitophagy | RNF2 | vesicle | CDKL1 |
| | | vesicle | CTAG2 | mitophagy,ER-MT contact, Sarcoplasmic reticulum-MT | VDAC1 | vesicle | SYNPO2 |
| | | vesicle | NMRK2 | MT biogenesis | LONP1 | vesicle | SET |
| | | vesicle | CCZ1B | MT fission | DNM1 | | |
| | | vesicle | ZFYVE9 | MT fission | KDR | | |
| | | | | MT fission | MX1 | | |
| | | | | MT fission | INF2 | | |
| | | | | MT fission | MYO19 | | |
| | | | | MT fission | LRRK2 | | |
| | | | | MT fission and fusion | OPA1 | | |
| | | | | MT fusion | MIGA2 | | |
| | | | | MT fusion, MT contact site | CHCHD3 | | |
| | | | | vesicle | ANKFY1 | | |
| | | | | vesicle | ABCD3 | | |
| | | | | vesicle | LAMP3 | | |
| | | | | vesicle | RUNX1 | | |
| | | | | vesicle | MBD1 | | |
| | | | | vesicle | NOV | | |
| | | | | vesicle | GTPBP2 | | |
| | | | | vesicle | GPRC5A | | |
| | | | | vesicle | PEX14 | | |
| | | | | vesicle | CSF2 | | |
| | | | | vesicle | KLRG1 | | |
| | | | | vesicle | ATP11A | | |
| | | | | vesicle | SPINK5 | | |
| | | | | vesicle | HEXIM1 | | |
| | | | | vesicle | PLCH1 | | |
| | | | | vesicle | ACAA1 | | |
| | | | | vesicle | RC3H2 | | |
| | | | | vesicle | HSD3B7 | | |
| | | | | vesicle | NACC1 | | |

## Slide 2
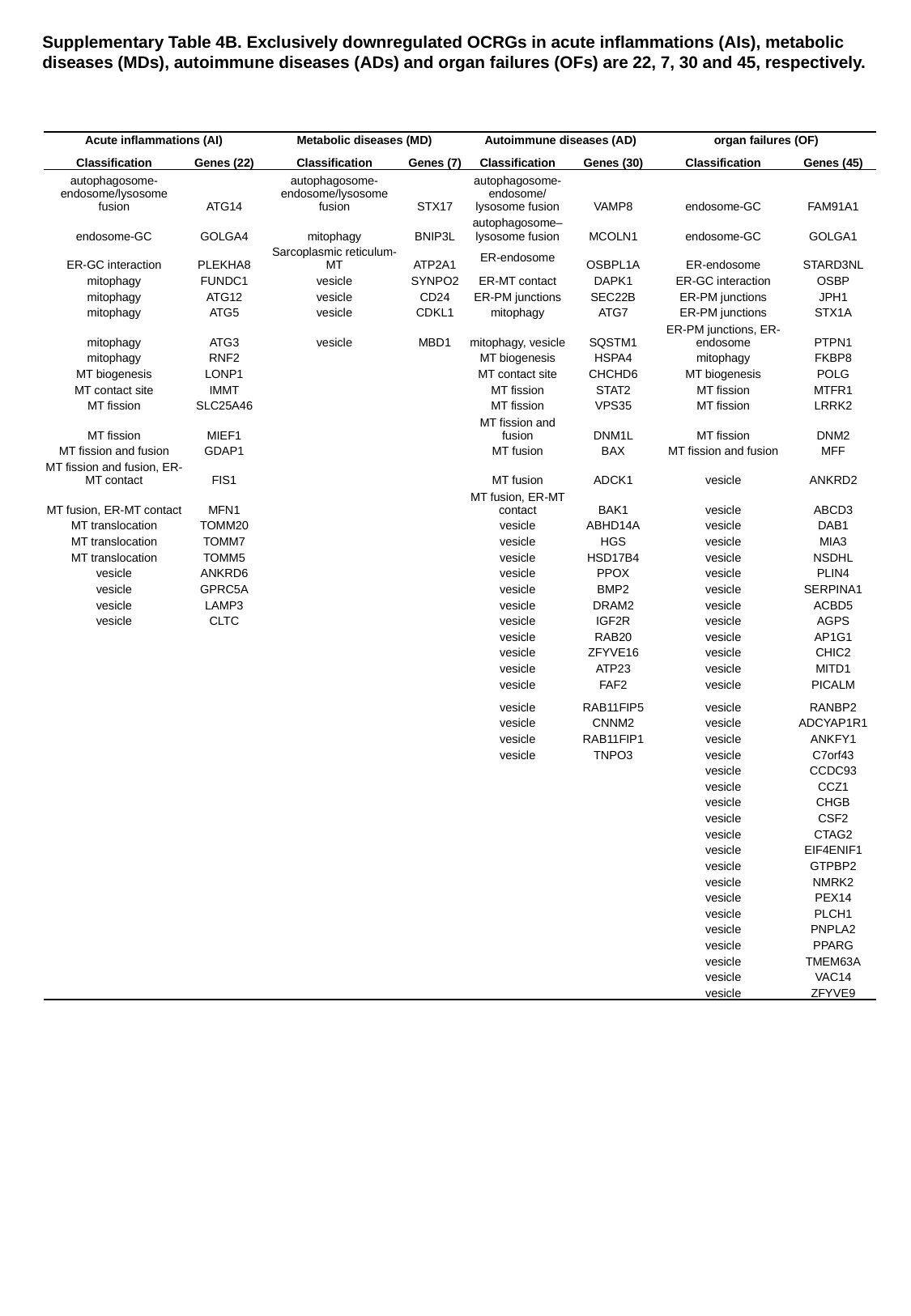

Supplementary Table 4B. Exclusively downregulated OCRGs in acute inflammations (AIs), metabolic diseases (MDs), autoimmune diseases (ADs) and organ failures (OFs) are 22, 7, 30 and 45, respectively.
| Acute inflammations (AI) | | Metabolic diseases (MD) | | Autoimmune diseases (AD) | | organ failures (OF) | |
| --- | --- | --- | --- | --- | --- | --- | --- |
| Classification | Genes (22) | Classification | Genes (7) | Classification | Genes (30) | Classification | Genes (45) |
| autophagosome-endosome/lysosome fusion | ATG14 | autophagosome-endosome/lysosome fusion | STX17 | autophagosome-endosome/lysosome fusion | VAMP8 | endosome-GC | FAM91A1 |
| endosome-GC | GOLGA4 | mitophagy | BNIP3L | autophagosome–lysosome fusion | MCOLN1 | endosome-GC | GOLGA1 |
| ER-GC interaction | PLEKHA8 | Sarcoplasmic reticulum-MT | ATP2A1 | ER-endosome | OSBPL1A | ER-endosome | STARD3NL |
| mitophagy | FUNDC1 | vesicle | SYNPO2 | ER-MT contact | DAPK1 | ER-GC interaction | OSBP |
| mitophagy | ATG12 | vesicle | CD24 | ER-PM junctions | SEC22B | ER-PM junctions | JPH1 |
| mitophagy | ATG5 | vesicle | CDKL1 | mitophagy | ATG7 | ER-PM junctions | STX1A |
| mitophagy | ATG3 | vesicle | MBD1 | mitophagy, vesicle | SQSTM1 | ER-PM junctions, ER-endosome | PTPN1 |
| mitophagy | RNF2 | | | MT biogenesis | HSPA4 | mitophagy | FKBP8 |
| MT biogenesis | LONP1 | | | MT contact site | CHCHD6 | MT biogenesis | POLG |
| MT contact site | IMMT | | | MT fission | STAT2 | MT fission | MTFR1 |
| MT fission | SLC25A46 | | | MT fission | VPS35 | MT fission | LRRK2 |
| MT fission | MIEF1 | | | MT fission and fusion | DNM1L | MT fission | DNM2 |
| MT fission and fusion | GDAP1 | | | MT fusion | BAX | MT fission and fusion | MFF |
| MT fission and fusion, ER-MT contact | FIS1 | | | MT fusion | ADCK1 | vesicle | ANKRD2 |
| MT fusion, ER-MT contact | MFN1 | | | MT fusion, ER-MT contact | BAK1 | vesicle | ABCD3 |
| MT translocation | TOMM20 | | | vesicle | ABHD14A | vesicle | DAB1 |
| MT translocation | TOMM7 | | | vesicle | HGS | vesicle | MIA3 |
| MT translocation | TOMM5 | | | vesicle | HSD17B4 | vesicle | NSDHL |
| vesicle | ANKRD6 | | | vesicle | PPOX | vesicle | PLIN4 |
| vesicle | GPRC5A | | | vesicle | BMP2 | vesicle | SERPINA1 |
| vesicle | LAMP3 | | | vesicle | DRAM2 | vesicle | ACBD5 |
| vesicle | CLTC | | | vesicle | IGF2R | vesicle | AGPS |
| | | | | vesicle | RAB20 | vesicle | AP1G1 |
| | | | | vesicle | ZFYVE16 | vesicle | CHIC2 |
| | | | | vesicle | ATP23 | vesicle | MITD1 |
| | | | | vesicle | FAF2 | vesicle | PICALM |
| | | | | vesicle | RAB11FIP5 | vesicle | RANBP2 |
| | | | | vesicle | CNNM2 | vesicle | ADCYAP1R1 |
| | | | | vesicle | RAB11FIP1 | vesicle | ANKFY1 |
| | | | | vesicle | TNPO3 | vesicle | C7orf43 |
| | | | | | | vesicle | CCDC93 |
| | | | | | | vesicle | CCZ1 |
| | | | | | | vesicle | CHGB |
| | | | | | | vesicle | CSF2 |
| | | | | | | vesicle | CTAG2 |
| | | | | | | vesicle | EIF4ENIF1 |
| | | | | | | vesicle | GTPBP2 |
| | | | | | | vesicle | NMRK2 |
| | | | | | | vesicle | PEX14 |
| | | | | | | vesicle | PLCH1 |
| | | | | | | vesicle | PNPLA2 |
| | | | | | | vesicle | PPARG |
| | | | | | | vesicle | TMEM63A |
| | | | | | | vesicle | VAC14 |
| | | | | | | vesicle | ZFYVE9 |

## Slide 3
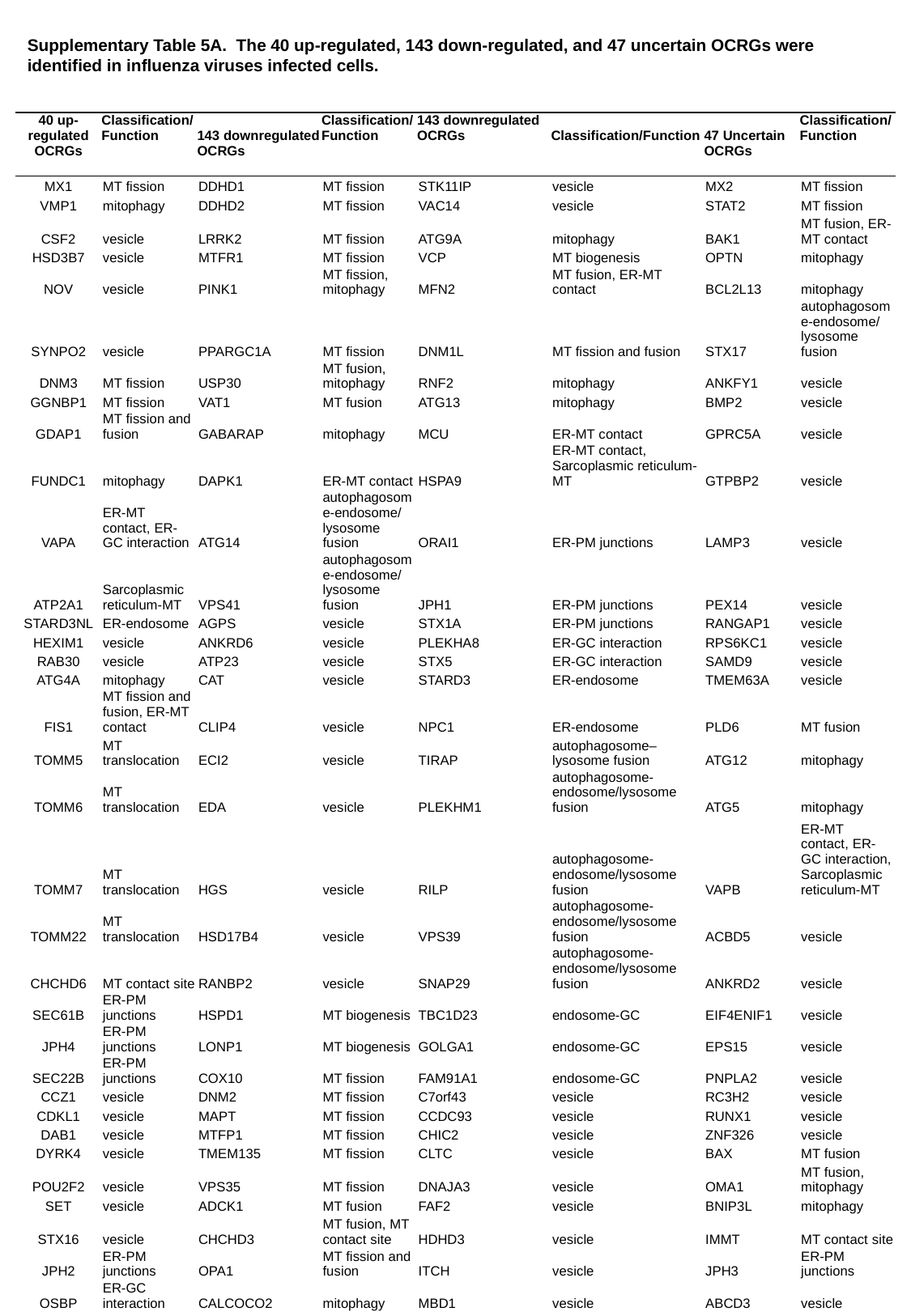

Supplementary Table 5A. The 40 up-regulated, 143 down-regulated, and 47 uncertain OCRGs were identified in influenza viruses infected cells.
| 40 up-regulated OCRGs | Classification/Function | 143 downregulated OCRGs | Classification/Function | 143 downregulated OCRGs | Classification/Function | 47 Uncertain OCRGs | Classification/Function |
| --- | --- | --- | --- | --- | --- | --- | --- |
| | | | | | | | |
| MX1 | MT fission | DDHD1 | MT fission | STK11IP | vesicle | MX2 | MT fission |
| VMP1 | mitophagy | DDHD2 | MT fission | VAC14 | vesicle | STAT2 | MT fission |
| CSF2 | vesicle | LRRK2 | MT fission | ATG9A | mitophagy | BAK1 | MT fusion, ER-MT contact |
| HSD3B7 | vesicle | MTFR1 | MT fission | VCP | MT biogenesis | OPTN | mitophagy |
| NOV | vesicle | PINK1 | MT fission, mitophagy | MFN2 | MT fusion, ER-MT contact | BCL2L13 | mitophagy |
| SYNPO2 | vesicle | PPARGC1A | MT fission | DNM1L | MT fission and fusion | STX17 | autophagosome-endosome/lysosome fusion |
| DNM3 | MT fission | USP30 | MT fusion, mitophagy | RNF2 | mitophagy | ANKFY1 | vesicle |
| GGNBP1 | MT fission | VAT1 | MT fusion | ATG13 | mitophagy | BMP2 | vesicle |
| GDAP1 | MT fission and fusion | GABARAP | mitophagy | MCU | ER-MT contact | GPRC5A | vesicle |
| FUNDC1 | mitophagy | DAPK1 | ER-MT contact | HSPA9 | ER-MT contact, Sarcoplasmic reticulum-MT | GTPBP2 | vesicle |
| VAPA | ER-MT contact, ER-GC interaction | ATG14 | autophagosome-endosome/lysosome fusion | ORAI1 | ER-PM junctions | LAMP3 | vesicle |
| ATP2A1 | Sarcoplasmic reticulum-MT | VPS41 | autophagosome-endosome/lysosome fusion | JPH1 | ER-PM junctions | PEX14 | vesicle |
| STARD3NL | ER-endosome | AGPS | vesicle | STX1A | ER-PM junctions | RANGAP1 | vesicle |
| HEXIM1 | vesicle | ANKRD6 | vesicle | PLEKHA8 | ER-GC interaction | RPS6KC1 | vesicle |
| RAB30 | vesicle | ATP23 | vesicle | STX5 | ER-GC interaction | SAMD9 | vesicle |
| ATG4A | mitophagy | CAT | vesicle | STARD3 | ER-endosome | TMEM63A | vesicle |
| FIS1 | MT fission and fusion, ER-MT contact | CLIP4 | vesicle | NPC1 | ER-endosome | PLD6 | MT fusion |
| TOMM5 | MT translocation | ECI2 | vesicle | TIRAP | autophagosome–lysosome fusion | ATG12 | mitophagy |
| TOMM6 | MT translocation | EDA | vesicle | PLEKHM1 | autophagosome-endosome/lysosome fusion | ATG5 | mitophagy |
| TOMM7 | MT translocation | HGS | vesicle | RILP | autophagosome-endosome/lysosome fusion | VAPB | ER-MT contact, ER-GC interaction, Sarcoplasmic reticulum-MT |
| TOMM22 | MT translocation | HSD17B4 | vesicle | VPS39 | autophagosome-endosome/lysosome fusion | ACBD5 | vesicle |
| CHCHD6 | MT contact site | RANBP2 | vesicle | SNAP29 | autophagosome-endosome/lysosome fusion | ANKRD2 | vesicle |
| SEC61B | ER-PM junctions | HSPD1 | MT biogenesis | TBC1D23 | endosome-GC | EIF4ENIF1 | vesicle |
| JPH4 | ER-PM junctions | LONP1 | MT biogenesis | GOLGA1 | endosome-GC | EPS15 | vesicle |
| SEC22B | ER-PM junctions | COX10 | MT fission | FAM91A1 | endosome-GC | PNPLA2 | vesicle |
| CCZ1 | vesicle | DNM2 | MT fission | C7orf43 | vesicle | RC3H2 | vesicle |
| CDKL1 | vesicle | MAPT | MT fission | CCDC93 | vesicle | RUNX1 | vesicle |
| DAB1 | vesicle | MTFP1 | MT fission | CHIC2 | vesicle | ZNF326 | vesicle |
| DYRK4 | vesicle | TMEM135 | MT fission | CLTC | vesicle | BAX | MT fusion |
| POU2F2 | vesicle | VPS35 | MT fission | DNAJA3 | vesicle | OMA1 | MT fusion, mitophagy |
| SET | vesicle | ADCK1 | MT fusion | FAF2 | vesicle | BNIP3L | mitophagy |
| STX16 | vesicle | CHCHD3 | MT fusion, MT contact site | HDHD3 | vesicle | IMMT | MT contact site |
| JPH2 | ER-PM junctions | OPA1 | MT fission and fusion | ITCH | vesicle | JPH3 | ER-PM junctions |
| OSBP | ER-GC interaction | CALCOCO2 | mitophagy | MBD1 | vesicle | ABCD3 | vesicle |
| | | SPINK5 | vesicle | | | | |

## Slide 4
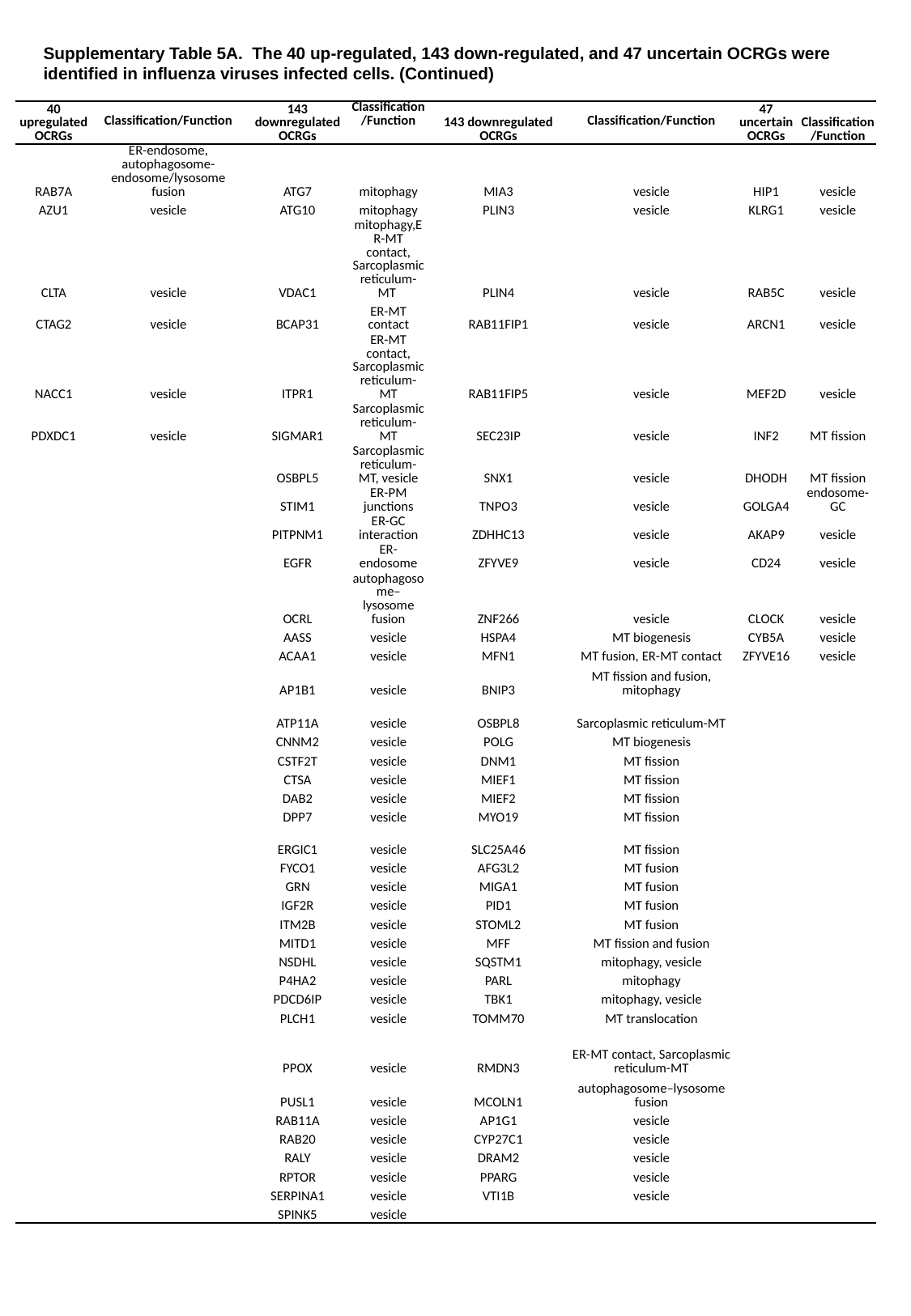

Supplementary Table 5A. The 40 up-regulated, 143 down-regulated, and 47 uncertain OCRGs were identified in influenza viruses infected cells. (Continued)
| 40 upregulated OCRGs | Classification/Function | 143 downregulated OCRGs | Classification/Function | 143 downregulated OCRGs | Classification/Function | 47 uncertain OCRGs | Classification/Function |
| --- | --- | --- | --- | --- | --- | --- | --- |
| RAB7A | ER-endosome, autophagosome-endosome/lysosome fusion | ATG7 | mitophagy | MIA3 | vesicle | HIP1 | vesicle |
| AZU1 | vesicle | ATG10 | mitophagy | PLIN3 | vesicle | KLRG1 | vesicle |
| CLTA | vesicle | VDAC1 | mitophagy,ER-MT contact, Sarcoplasmic reticulum-MT | PLIN4 | vesicle | RAB5C | vesicle |
| CTAG2 | vesicle | BCAP31 | ER-MT contact | RAB11FIP1 | vesicle | ARCN1 | vesicle |
| NACC1 | vesicle | ITPR1 | ER-MT contact, Sarcoplasmic reticulum-MT | RAB11FIP5 | vesicle | MEF2D | vesicle |
| PDXDC1 | vesicle | SIGMAR1 | Sarcoplasmic reticulum-MT | SEC23IP | vesicle | INF2 | MT fission |
| | | OSBPL5 | Sarcoplasmic reticulum-MT, vesicle | SNX1 | vesicle | DHODH | MT fission |
| | | STIM1 | ER-PM junctions | TNPO3 | vesicle | GOLGA4 | endosome-GC |
| | | PITPNM1 | ER-GC interaction | ZDHHC13 | vesicle | AKAP9 | vesicle |
| | | EGFR | ER-endosome | ZFYVE9 | vesicle | CD24 | vesicle |
| | | OCRL | autophagosome–lysosome fusion | ZNF266 | vesicle | CLOCK | vesicle |
| | | AASS | vesicle | HSPA4 | MT biogenesis | CYB5A | vesicle |
| | | ACAA1 | vesicle | MFN1 | MT fusion, ER-MT contact | ZFYVE16 | vesicle |
| | | AP1B1 | vesicle | BNIP3 | MT fission and fusion, mitophagy | | |
| | | ATP11A | vesicle | OSBPL8 | Sarcoplasmic reticulum-MT | | |
| | | CNNM2 | vesicle | POLG | MT biogenesis | | |
| | | CSTF2T | vesicle | DNM1 | MT fission | | |
| | | CTSA | vesicle | MIEF1 | MT fission | | |
| | | DAB2 | vesicle | MIEF2 | MT fission | | |
| | | DPP7 | vesicle | MYO19 | MT fission | | |
| | | ERGIC1 | vesicle | SLC25A46 | MT fission | | |
| | | FYCO1 | vesicle | AFG3L2 | MT fusion | | |
| | | GRN | vesicle | MIGA1 | MT fusion | | |
| | | IGF2R | vesicle | PID1 | MT fusion | | |
| | | ITM2B | vesicle | STOML2 | MT fusion | | |
| | | MITD1 | vesicle | MFF | MT fission and fusion | | |
| | | NSDHL | vesicle | SQSTM1 | mitophagy, vesicle | | |
| | | P4HA2 | vesicle | PARL | mitophagy | | |
| | | PDCD6IP | vesicle | TBK1 | mitophagy, vesicle | | |
| | | PLCH1 | vesicle | TOMM70 | MT translocation | | |
| | | PPOX | vesicle | RMDN3 | ER-MT contact, Sarcoplasmic reticulum-MT | | |
| | | PUSL1 | vesicle | MCOLN1 | autophagosome–lysosome fusion | | |
| | | RAB11A | vesicle | AP1G1 | vesicle | | |
| | | RAB20 | vesicle | CYP27C1 | vesicle | | |
| | | RALY | vesicle | DRAM2 | vesicle | | |
| | | RPTOR | vesicle | PPARG | vesicle | | |
| | | SERPINA1 | vesicle | VTI1B | vesicle | | |
| | | SPINK5 | vesicle | | | | |

## Slide 5
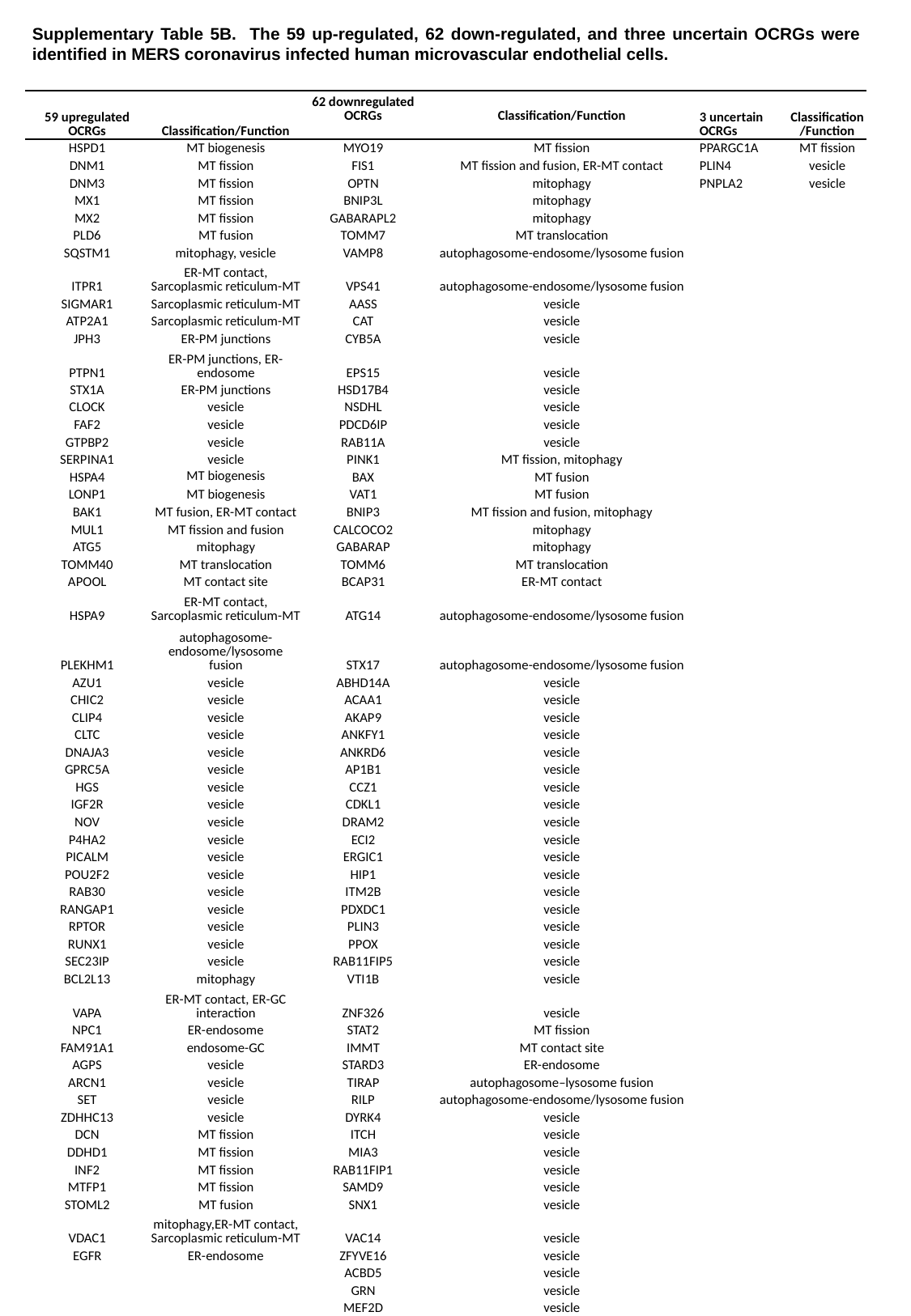

Supplementary Table 5B. The 59 up-regulated, 62 down-regulated, and three uncertain OCRGs were identified in MERS coronavirus infected human microvascular endothelial cells.
| 59 upregulated OCRGs | Classification/Function | 62 downregulated OCRGs | Classification/Function | 3 uncertain OCRGs | Classification/Function |
| --- | --- | --- | --- | --- | --- |
| HSPD1 | MT biogenesis | MYO19 | MT fission | PPARGC1A | MT fission |
| DNM1 | MT fission | FIS1 | MT fission and fusion, ER-MT contact | PLIN4 | vesicle |
| DNM3 | MT fission | OPTN | mitophagy | PNPLA2 | vesicle |
| MX1 | MT fission | BNIP3L | mitophagy | | |
| MX2 | MT fission | GABARAPL2 | mitophagy | | |
| PLD6 | MT fusion | TOMM7 | MT translocation | | |
| SQSTM1 | mitophagy, vesicle | VAMP8 | autophagosome-endosome/lysosome fusion | | |
| ITPR1 | ER-MT contact, Sarcoplasmic reticulum-MT | VPS41 | autophagosome-endosome/lysosome fusion | | |
| SIGMAR1 | Sarcoplasmic reticulum-MT | AASS | vesicle | | |
| ATP2A1 | Sarcoplasmic reticulum-MT | CAT | vesicle | | |
| JPH3 | ER-PM junctions | CYB5A | vesicle | | |
| PTPN1 | ER-PM junctions, ER-endosome | EPS15 | vesicle | | |
| STX1A | ER-PM junctions | HSD17B4 | vesicle | | |
| CLOCK | vesicle | NSDHL | vesicle | | |
| FAF2 | vesicle | PDCD6IP | vesicle | | |
| GTPBP2 | vesicle | RAB11A | vesicle | | |
| SERPINA1 | vesicle | PINK1 | MT fission, mitophagy | | |
| HSPA4 | MT biogenesis | BAX | MT fusion | | |
| LONP1 | MT biogenesis | VAT1 | MT fusion | | |
| BAK1 | MT fusion, ER-MT contact | BNIP3 | MT fission and fusion, mitophagy | | |
| MUL1 | MT fission and fusion | CALCOCO2 | mitophagy | | |
| ATG5 | mitophagy | GABARAP | mitophagy | | |
| TOMM40 | MT translocation | TOMM6 | MT translocation | | |
| APOOL | MT contact site | BCAP31 | ER-MT contact | | |
| HSPA9 | ER-MT contact, Sarcoplasmic reticulum-MT | ATG14 | autophagosome-endosome/lysosome fusion | | |
| PLEKHM1 | autophagosome-endosome/lysosome fusion | STX17 | autophagosome-endosome/lysosome fusion | | |
| AZU1 | vesicle | ABHD14A | vesicle | | |
| CHIC2 | vesicle | ACAA1 | vesicle | | |
| CLIP4 | vesicle | AKAP9 | vesicle | | |
| CLTC | vesicle | ANKFY1 | vesicle | | |
| DNAJA3 | vesicle | ANKRD6 | vesicle | | |
| GPRC5A | vesicle | AP1B1 | vesicle | | |
| HGS | vesicle | CCZ1 | vesicle | | |
| IGF2R | vesicle | CDKL1 | vesicle | | |
| NOV | vesicle | DRAM2 | vesicle | | |
| P4HA2 | vesicle | ECI2 | vesicle | | |
| PICALM | vesicle | ERGIC1 | vesicle | | |
| POU2F2 | vesicle | HIP1 | vesicle | | |
| RAB30 | vesicle | ITM2B | vesicle | | |
| RANGAP1 | vesicle | PDXDC1 | vesicle | | |
| RPTOR | vesicle | PLIN3 | vesicle | | |
| RUNX1 | vesicle | PPOX | vesicle | | |
| SEC23IP | vesicle | RAB11FIP5 | vesicle | | |
| BCL2L13 | mitophagy | VTI1B | vesicle | | |
| VAPA | ER-MT contact, ER-GC interaction | ZNF326 | vesicle | | |
| NPC1 | ER-endosome | STAT2 | MT fission | | |
| FAM91A1 | endosome-GC | IMMT | MT contact site | | |
| AGPS | vesicle | STARD3 | ER-endosome | | |
| ARCN1 | vesicle | TIRAP | autophagosome–lysosome fusion | | |
| SET | vesicle | RILP | autophagosome-endosome/lysosome fusion | | |
| ZDHHC13 | vesicle | DYRK4 | vesicle | | |
| DCN | MT fission | ITCH | vesicle | | |
| DDHD1 | MT fission | MIA3 | vesicle | | |
| INF2 | MT fission | RAB11FIP1 | vesicle | | |
| MTFP1 | MT fission | SAMD9 | vesicle | | |
| STOML2 | MT fusion | SNX1 | vesicle | | |
| VDAC1 | mitophagy,ER-MT contact, Sarcoplasmic reticulum-MT | VAC14 | vesicle | | |
| EGFR | ER-endosome | ZFYVE16 | vesicle | | |
| | | ACBD5 | vesicle | | |
| | | GRN | vesicle | | |
| | | MEF2D | vesicle | | |

## Slide 6
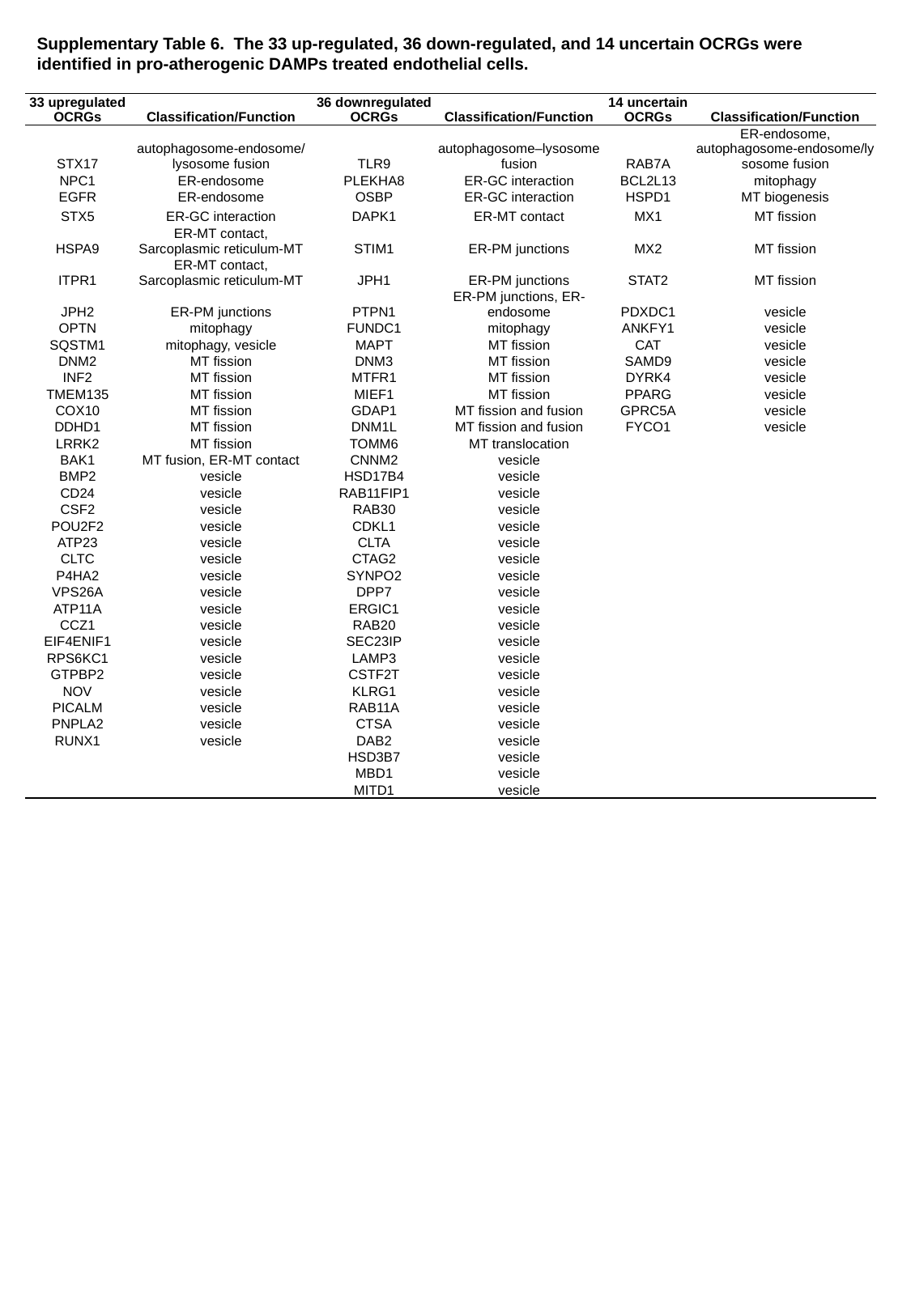

Supplementary Table 6. The 33 up-regulated, 36 down-regulated, and 14 uncertain OCRGs were identified in pro-atherogenic DAMPs treated endothelial cells.
| 33 upregulated OCRGs | Classification/Function | 36 downregulated OCRGs | Classification/Function | 14 uncertain OCRGs | Classification/Function |
| --- | --- | --- | --- | --- | --- |
| STX17 | autophagosome-endosome/lysosome fusion | TLR9 | autophagosome–lysosome fusion | RAB7A | ER-endosome, autophagosome-endosome/lysosome fusion |
| NPC1 | ER-endosome | PLEKHA8 | ER-GC interaction | BCL2L13 | mitophagy |
| EGFR | ER-endosome | OSBP | ER-GC interaction | HSPD1 | MT biogenesis |
| STX5 | ER-GC interaction | DAPK1 | ER-MT contact | MX1 | MT fission |
| HSPA9 | ER-MT contact, Sarcoplasmic reticulum-MT | STIM1 | ER-PM junctions | MX2 | MT fission |
| ITPR1 | ER-MT contact, Sarcoplasmic reticulum-MT | JPH1 | ER-PM junctions | STAT2 | MT fission |
| JPH2 | ER-PM junctions | PTPN1 | ER-PM junctions, ER-endosome | PDXDC1 | vesicle |
| OPTN | mitophagy | FUNDC1 | mitophagy | ANKFY1 | vesicle |
| SQSTM1 | mitophagy, vesicle | MAPT | MT fission | CAT | vesicle |
| DNM2 | MT fission | DNM3 | MT fission | SAMD9 | vesicle |
| INF2 | MT fission | MTFR1 | MT fission | DYRK4 | vesicle |
| TMEM135 | MT fission | MIEF1 | MT fission | PPARG | vesicle |
| COX10 | MT fission | GDAP1 | MT fission and fusion | GPRC5A | vesicle |
| DDHD1 | MT fission | DNM1L | MT fission and fusion | FYCO1 | vesicle |
| LRRK2 | MT fission | TOMM6 | MT translocation | | |
| BAK1 | MT fusion, ER-MT contact | CNNM2 | vesicle | | |
| BMP2 | vesicle | HSD17B4 | vesicle | | |
| CD24 | vesicle | RAB11FIP1 | vesicle | | |
| CSF2 | vesicle | RAB30 | vesicle | | |
| POU2F2 | vesicle | CDKL1 | vesicle | | |
| ATP23 | vesicle | CLTA | vesicle | | |
| CLTC | vesicle | CTAG2 | vesicle | | |
| P4HA2 | vesicle | SYNPO2 | vesicle | | |
| VPS26A | vesicle | DPP7 | vesicle | | |
| ATP11A | vesicle | ERGIC1 | vesicle | | |
| CCZ1 | vesicle | RAB20 | vesicle | | |
| EIF4ENIF1 | vesicle | SEC23IP | vesicle | | |
| RPS6KC1 | vesicle | LAMP3 | vesicle | | |
| GTPBP2 | vesicle | CSTF2T | vesicle | | |
| NOV | vesicle | KLRG1 | vesicle | | |
| PICALM | vesicle | RAB11A | vesicle | | |
| PNPLA2 | vesicle | CTSA | vesicle | | |
| RUNX1 | vesicle | DAB2 | vesicle | | |
| | | HSD3B7 | vesicle | | |
| | | MBD1 | vesicle | | |
| | | MITD1 | vesicle | | |

## Slide 7
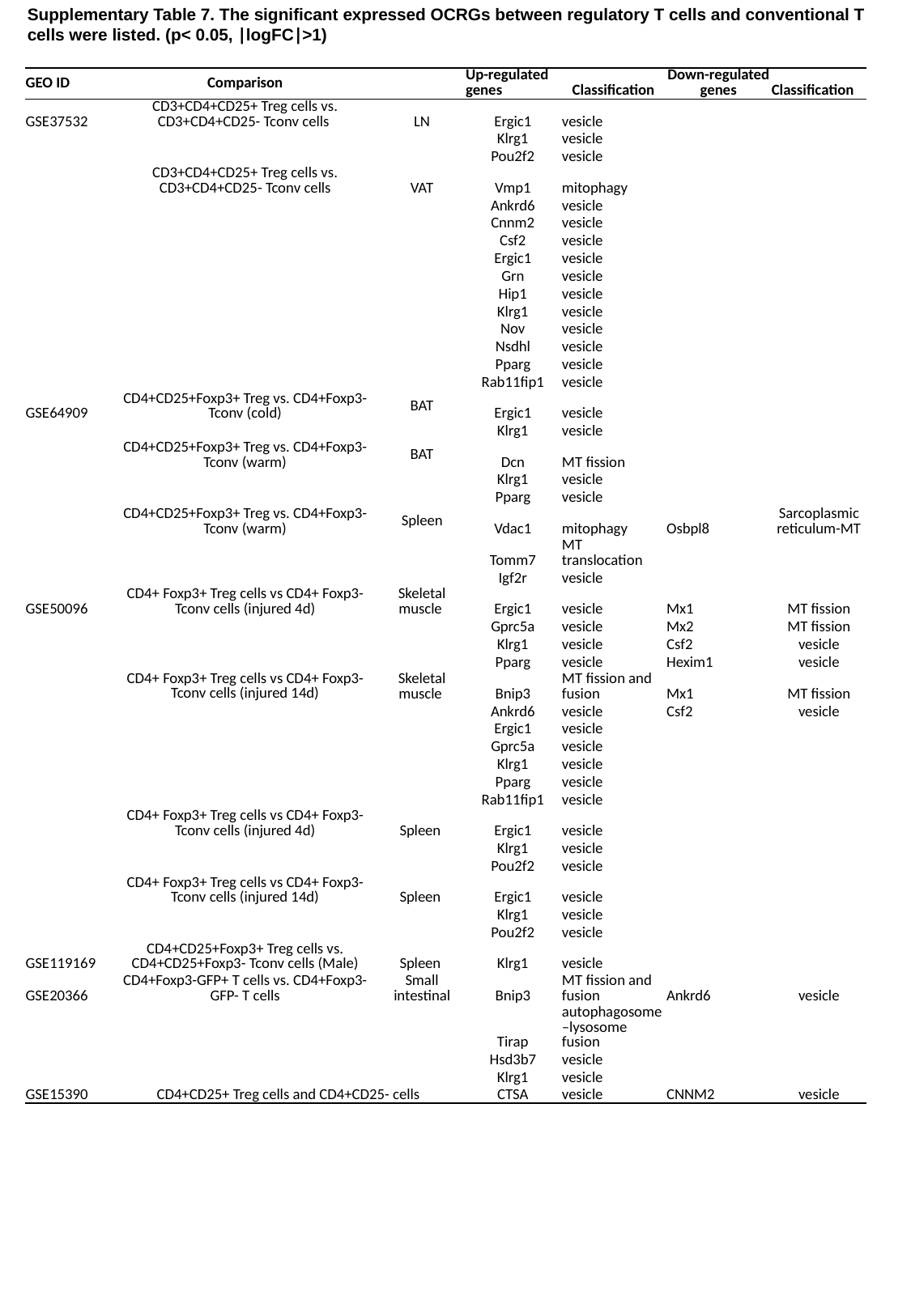

Supplementary Table 7. The significant expressed OCRGs between regulatory T cells and conventional T cells were listed. (p< 0.05, ∣logFC∣>1)
| GEO ID | Comparison | | Up-regulated genes | Classification | Down-regulated genes | Classification |
| --- | --- | --- | --- | --- | --- | --- |
| GSE37532 | CD3+CD4+CD25+ Treg cells vs. CD3+CD4+CD25- Tconv cells | LN | Ergic1 | vesicle | | |
| | | | Klrg1 | vesicle | | |
| | | | Pou2f2 | vesicle | | |
| | CD3+CD4+CD25+ Treg cells vs. CD3+CD4+CD25- Tconv cells | VAT | Vmp1 | mitophagy | | |
| | | | Ankrd6 | vesicle | | |
| | | | Cnnm2 | vesicle | | |
| | | | Csf2 | vesicle | | |
| | | | Ergic1 | vesicle | | |
| | | | Grn | vesicle | | |
| | | | Hip1 | vesicle | | |
| | | | Klrg1 | vesicle | | |
| | | | Nov | vesicle | | |
| | | | Nsdhl | vesicle | | |
| | | | Pparg | vesicle | | |
| | | | Rab11fip1 | vesicle | | |
| GSE64909 | CD4+CD25+Foxp3+ Treg vs. CD4+Foxp3- Tconv (cold) | BAT | Ergic1 | vesicle | | |
| | | | Klrg1 | vesicle | | |
| | CD4+CD25+Foxp3+ Treg vs. CD4+Foxp3- Tconv (warm) | BAT | Dcn | MT fission | | |
| | | | Klrg1 | vesicle | | |
| | | | Pparg | vesicle | | |
| | CD4+CD25+Foxp3+ Treg vs. CD4+Foxp3- Tconv (warm) | Spleen | Vdac1 | mitophagy | Osbpl8 | Sarcoplasmic reticulum-MT |
| | | | Tomm7 | MT translocation | | |
| | | | Igf2r | vesicle | | |
| GSE50096 | CD4+ Foxp3+ Treg cells vs CD4+ Foxp3- Tconv cells (injured 4d) | Skeletal muscle | Ergic1 | vesicle | Mx1 | MT fission |
| | | | Gprc5a | vesicle | Mx2 | MT fission |
| | | | Klrg1 | vesicle | Csf2 | vesicle |
| | | | Pparg | vesicle | Hexim1 | vesicle |
| | CD4+ Foxp3+ Treg cells vs CD4+ Foxp3- Tconv cells (injured 14d) | Skeletal muscle | Bnip3 | MT fission and fusion | Mx1 | MT fission |
| | | | Ankrd6 | vesicle | Csf2 | vesicle |
| | | | Ergic1 | vesicle | | |
| | | | Gprc5a | vesicle | | |
| | | | Klrg1 | vesicle | | |
| | | | Pparg | vesicle | | |
| | | | Rab11fip1 | vesicle | | |
| | CD4+ Foxp3+ Treg cells vs CD4+ Foxp3- Tconv cells (injured 4d) | Spleen | Ergic1 | vesicle | | |
| | | | Klrg1 | vesicle | | |
| | | | Pou2f2 | vesicle | | |
| | CD4+ Foxp3+ Treg cells vs CD4+ Foxp3- Tconv cells (injured 14d) | Spleen | Ergic1 | vesicle | | |
| | | | Klrg1 | vesicle | | |
| | | | Pou2f2 | vesicle | | |
| GSE119169 | CD4+CD25+Foxp3+ Treg cells vs. CD4+CD25+Foxp3- Tconv cells (Male) | Spleen | Klrg1 | vesicle | | |
| GSE20366 | CD4+Foxp3-GFP+ T cells vs. CD4+Foxp3-GFP- T cells | Small intestinal | Bnip3 | MT fission and fusion | Ankrd6 | vesicle |
| | | | Tirap | autophagosome–lysosome fusion | | |
| | | | Hsd3b7 | vesicle | | |
| | | | Klrg1 | vesicle | | |
| GSE15390 | CD4+CD25+ Treg cells and CD4+CD25- cells | | CTSA | vesicle | CNNM2 | vesicle |

## Slide 8
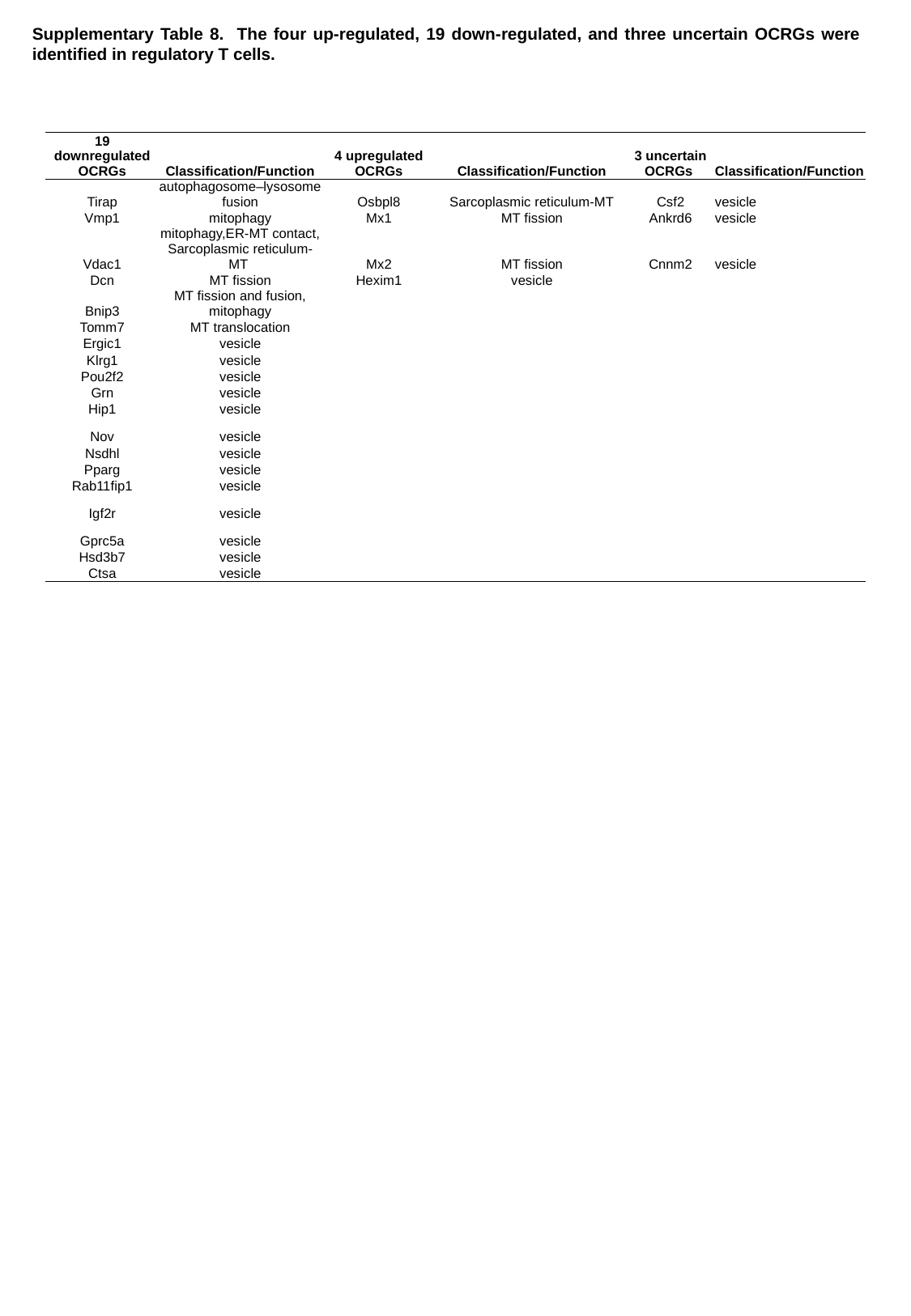

Supplementary Table 8. The four up-regulated, 19 down-regulated, and three uncertain OCRGs were identified in regulatory T cells.
| 19 downregulated OCRGs | Classification/Function | 4 upregulated OCRGs | Classification/Function | 3 uncertain OCRGs | Classification/Function | |
| --- | --- | --- | --- | --- | --- | --- |
| Tirap | autophagosome–lysosome fusion | Osbpl8 | Sarcoplasmic reticulum-MT | Csf2 | vesicle | |
| Vmp1 | mitophagy | Mx1 | MT fission | Ankrd6 | vesicle | |
| Vdac1 | mitophagy,ER-MT contact, Sarcoplasmic reticulum-MT | Mx2 | MT fission | Cnnm2 | vesicle | |
| Dcn | MT fission | Hexim1 | vesicle | | | |
| Bnip3 | MT fission and fusion, mitophagy | | | | | |
| Tomm7 | MT translocation | | | | | |
| Ergic1 | vesicle | | | | | |
| Klrg1 | vesicle | | | | | |
| Pou2f2 | vesicle | | | | | |
| Grn | vesicle | | | | | |
| Hip1 | vesicle | | | | | |
| Nov | vesicle | | | | | |
| Nsdhl | vesicle | | | | | |
| Pparg | vesicle | | | | | |
| Rab11fip1 | vesicle | | | | | |
| Igf2r | vesicle | | | | | |
| Gprc5a | vesicle | | | | | |
| Hsd3b7 | vesicle | | | | | |
| Ctsa | vesicle | | | | | |

## Slide 9
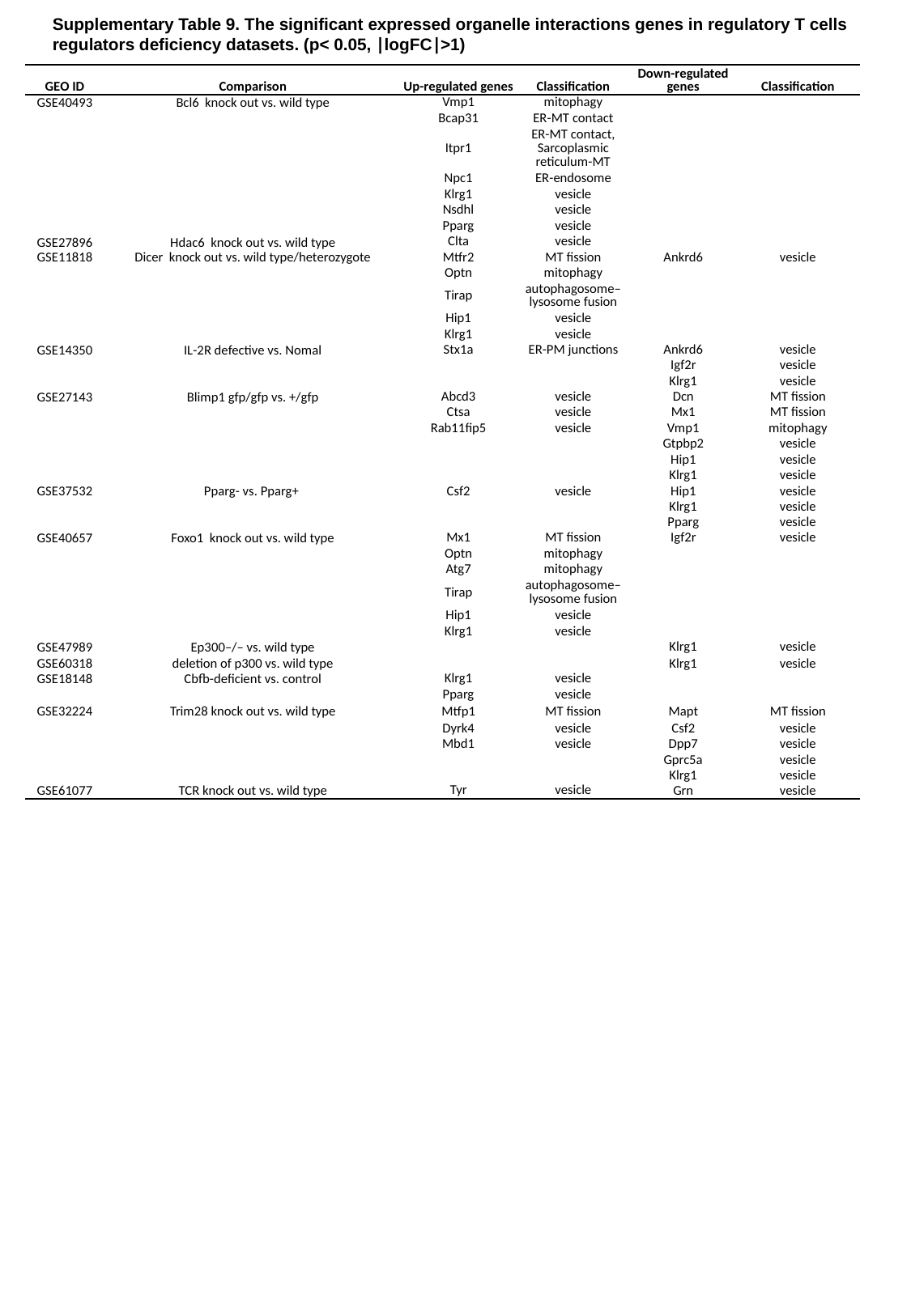

Supplementary Table 9. The significant expressed organelle interactions genes in regulatory T cells regulators deficiency datasets. (p< 0.05, ∣logFC∣>1)
| GEO ID | Comparison | Up-regulated genes | Classification | Down-regulated genes | Classification |
| --- | --- | --- | --- | --- | --- |
| GSE40493 | Bcl6 knock out vs. wild type | Vmp1 | mitophagy | | |
| | | Bcap31 | ER-MT contact | | |
| | | Itpr1 | ER-MT contact, Sarcoplasmic reticulum-MT | | |
| | | Npc1 | ER-endosome | | |
| | | Klrg1 | vesicle | | |
| | | Nsdhl | vesicle | | |
| | | Pparg | vesicle | | |
| GSE27896 | Hdac6 knock out vs. wild type | Clta | vesicle | | |
| GSE11818 | Dicer knock out vs. wild type/heterozygote | Mtfr2 | MT fission | Ankrd6 | vesicle |
| | | Optn | mitophagy | | |
| | | Tirap | autophagosome–lysosome fusion | | |
| | | Hip1 | vesicle | | |
| | | Klrg1 | vesicle | | |
| GSE14350 | IL-2R defective vs. Nomal | Stx1a | ER-PM junctions | Ankrd6 | vesicle |
| | | | | Igf2r | vesicle |
| | | | | Klrg1 | vesicle |
| GSE27143 | Blimp1 gfp/gfp vs. +/gfp | Abcd3 | vesicle | Dcn | MT fission |
| | | Ctsa | vesicle | Mx1 | MT fission |
| | | Rab11fip5 | vesicle | Vmp1 | mitophagy |
| | | | | Gtpbp2 | vesicle |
| | | | | Hip1 | vesicle |
| | | | | Klrg1 | vesicle |
| GSE37532 | Pparg- vs. Pparg+ | Csf2 | vesicle | Hip1 | vesicle |
| | | | | Klrg1 | vesicle |
| | | | | Pparg | vesicle |
| GSE40657 | Foxo1 knock out vs. wild type | Mx1 | MT fission | Igf2r | vesicle |
| | | Optn | mitophagy | | |
| | | Atg7 | mitophagy | | |
| | | Tirap | autophagosome–lysosome fusion | | |
| | | Hip1 | vesicle | | |
| | | Klrg1 | vesicle | | |
| GSE47989 | Ep300–/– vs. wild type | | | Klrg1 | vesicle |
| GSE60318 | deletion of p300 vs. wild type | | | Klrg1 | vesicle |
| GSE18148 | Cbfb-deficient vs. control | Klrg1 | vesicle | | |
| | | Pparg | vesicle | | |
| GSE32224 | Trim28 knock out vs. wild type | Mtfp1 | MT fission | Mapt | MT fission |
| | | Dyrk4 | vesicle | Csf2 | vesicle |
| | | Mbd1 | vesicle | Dpp7 | vesicle |
| | | | | Gprc5a | vesicle |
| | | | | Klrg1 | vesicle |
| GSE61077 | TCR knock out vs. wild type | Tyr | vesicle | Grn | vesicle |

## Slide 10
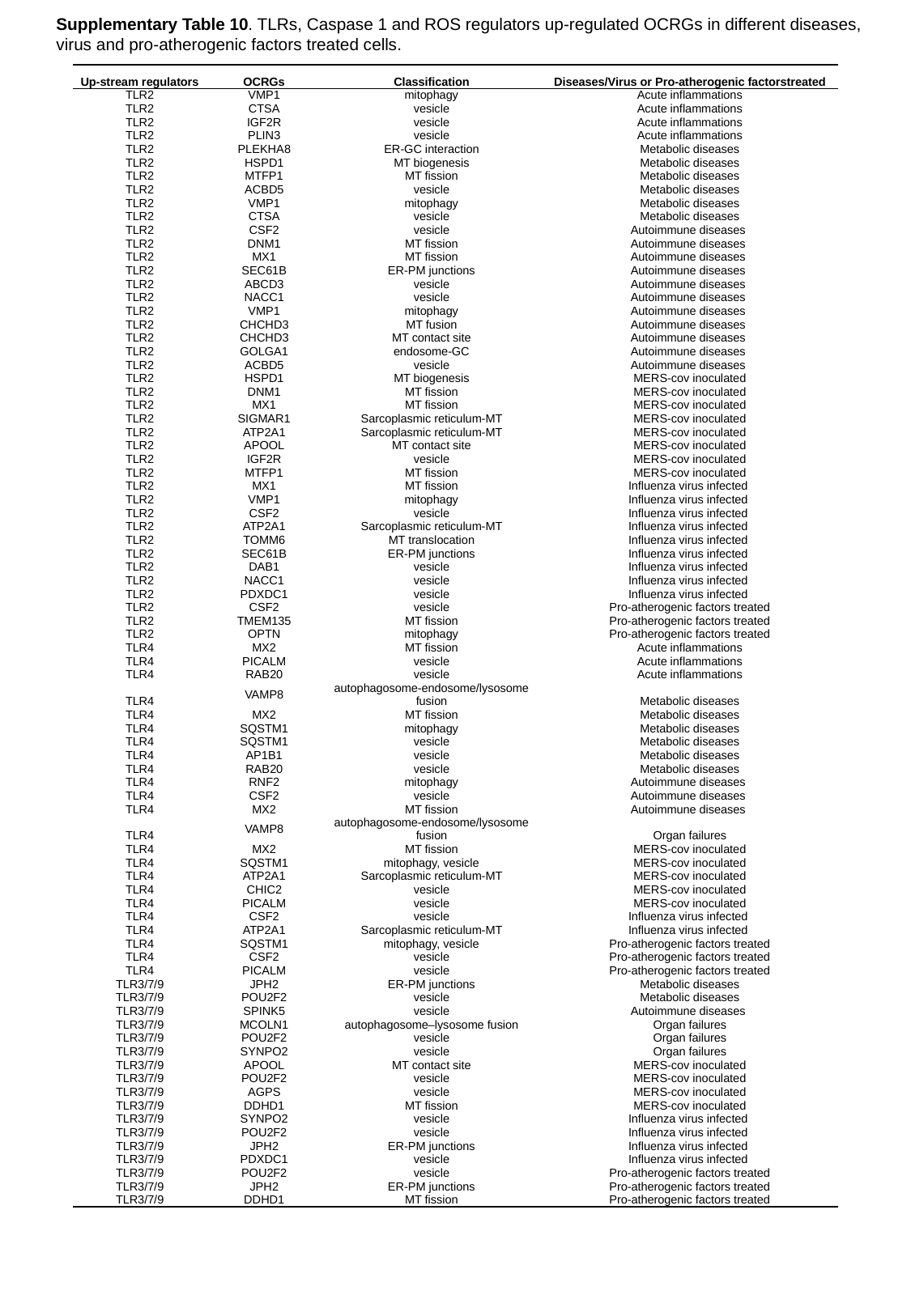

Supplementary Table 10. TLRs, Caspase 1 and ROS regulators up-regulated OCRGs in different diseases, virus and pro-atherogenic factors treated cells.
| Up-stream regulators | OCRGs | Classification | Diseases/Virus or Pro-atherogenic factorstreated |
| --- | --- | --- | --- |
| TLR2 | VMP1 | mitophagy | Acute inflammations |
| TLR2 | CTSA | vesicle | Acute inflammations |
| TLR2 | IGF2R | vesicle | Acute inflammations |
| TLR2 | PLIN3 | vesicle | Acute inflammations |
| TLR2 | PLEKHA8 | ER-GC interaction | Metabolic diseases |
| TLR2 | HSPD1 | MT biogenesis | Metabolic diseases |
| TLR2 | MTFP1 | MT fission | Metabolic diseases |
| TLR2 | ACBD5 | vesicle | Metabolic diseases |
| TLR2 | VMP1 | mitophagy | Metabolic diseases |
| TLR2 | CTSA | vesicle | Metabolic diseases |
| TLR2 | CSF2 | vesicle | Autoimmune diseases |
| TLR2 | DNM1 | MT fission | Autoimmune diseases |
| TLR2 | MX1 | MT fission | Autoimmune diseases |
| TLR2 | SEC61B | ER-PM junctions | Autoimmune diseases |
| TLR2 | ABCD3 | vesicle | Autoimmune diseases |
| TLR2 | NACC1 | vesicle | Autoimmune diseases |
| TLR2 | VMP1 | mitophagy | Autoimmune diseases |
| TLR2 | CHCHD3 | MT fusion | Autoimmune diseases |
| TLR2 | CHCHD3 | MT contact site | Autoimmune diseases |
| TLR2 | GOLGA1 | endosome-GC | Autoimmune diseases |
| TLR2 | ACBD5 | vesicle | Autoimmune diseases |
| TLR2 | HSPD1 | MT biogenesis | MERS-cov inoculated |
| TLR2 | DNM1 | MT fission | MERS-cov inoculated |
| TLR2 | MX1 | MT fission | MERS-cov inoculated |
| TLR2 | SIGMAR1 | Sarcoplasmic reticulum-MT | MERS-cov inoculated |
| TLR2 | ATP2A1 | Sarcoplasmic reticulum-MT | MERS-cov inoculated |
| TLR2 | APOOL | MT contact site | MERS-cov inoculated |
| TLR2 | IGF2R | vesicle | MERS-cov inoculated |
| TLR2 | MTFP1 | MT fission | MERS-cov inoculated |
| TLR2 | MX1 | MT fission | Influenza virus infected |
| TLR2 | VMP1 | mitophagy | Influenza virus infected |
| TLR2 | CSF2 | vesicle | Influenza virus infected |
| TLR2 | ATP2A1 | Sarcoplasmic reticulum-MT | Influenza virus infected |
| TLR2 | TOMM6 | MT translocation | Influenza virus infected |
| TLR2 | SEC61B | ER-PM junctions | Influenza virus infected |
| TLR2 | DAB1 | vesicle | Influenza virus infected |
| TLR2 | NACC1 | vesicle | Influenza virus infected |
| TLR2 | PDXDC1 | vesicle | Influenza virus infected |
| TLR2 | CSF2 | vesicle | Pro-atherogenic factors treated |
| TLR2 | TMEM135 | MT fission | Pro-atherogenic factors treated |
| TLR2 | OPTN | mitophagy | Pro-atherogenic factors treated |
| TLR4 | MX2 | MT fission | Acute inflammations |
| TLR4 | PICALM | vesicle | Acute inflammations |
| TLR4 | RAB20 | vesicle | Acute inflammations |
| TLR4 | VAMP8 | autophagosome-endosome/lysosome fusion | Metabolic diseases |
| TLR4 | MX2 | MT fission | Metabolic diseases |
| TLR4 | SQSTM1 | mitophagy | Metabolic diseases |
| TLR4 | SQSTM1 | vesicle | Metabolic diseases |
| TLR4 | AP1B1 | vesicle | Metabolic diseases |
| TLR4 | RAB20 | vesicle | Metabolic diseases |
| TLR4 | RNF2 | mitophagy | Autoimmune diseases |
| TLR4 | CSF2 | vesicle | Autoimmune diseases |
| TLR4 | MX2 | MT fission | Autoimmune diseases |
| TLR4 | VAMP8 | autophagosome-endosome/lysosome fusion | Organ failures |
| TLR4 | MX2 | MT fission | MERS-cov inoculated |
| TLR4 | SQSTM1 | mitophagy, vesicle | MERS-cov inoculated |
| TLR4 | ATP2A1 | Sarcoplasmic reticulum-MT | MERS-cov inoculated |
| TLR4 | CHIC2 | vesicle | MERS-cov inoculated |
| TLR4 | PICALM | vesicle | MERS-cov inoculated |
| TLR4 | CSF2 | vesicle | Influenza virus infected |
| TLR4 | ATP2A1 | Sarcoplasmic reticulum-MT | Influenza virus infected |
| TLR4 | SQSTM1 | mitophagy, vesicle | Pro-atherogenic factors treated |
| TLR4 | CSF2 | vesicle | Pro-atherogenic factors treated |
| TLR4 | PICALM | vesicle | Pro-atherogenic factors treated |
| TLR3/7/9 | JPH2 | ER-PM junctions | Metabolic diseases |
| TLR3/7/9 | POU2F2 | vesicle | Metabolic diseases |
| TLR3/7/9 | SPINK5 | vesicle | Autoimmune diseases |
| TLR3/7/9 | MCOLN1 | autophagosome–lysosome fusion | Organ failures |
| TLR3/7/9 | POU2F2 | vesicle | Organ failures |
| TLR3/7/9 | SYNPO2 | vesicle | Organ failures |
| TLR3/7/9 | APOOL | MT contact site | MERS-cov inoculated |
| TLR3/7/9 | POU2F2 | vesicle | MERS-cov inoculated |
| TLR3/7/9 | AGPS | vesicle | MERS-cov inoculated |
| TLR3/7/9 | DDHD1 | MT fission | MERS-cov inoculated |
| TLR3/7/9 | SYNPO2 | vesicle | Influenza virus infected |
| TLR3/7/9 | POU2F2 | vesicle | Influenza virus infected |
| TLR3/7/9 | JPH2 | ER-PM junctions | Influenza virus infected |
| TLR3/7/9 | PDXDC1 | vesicle | Influenza virus infected |
| TLR3/7/9 | POU2F2 | vesicle | Pro-atherogenic factors treated |
| TLR3/7/9 | JPH2 | ER-PM junctions | Pro-atherogenic factors treated |
| TLR3/7/9 | DDHD1 | MT fission | Pro-atherogenic factors treated |

## Slide 11
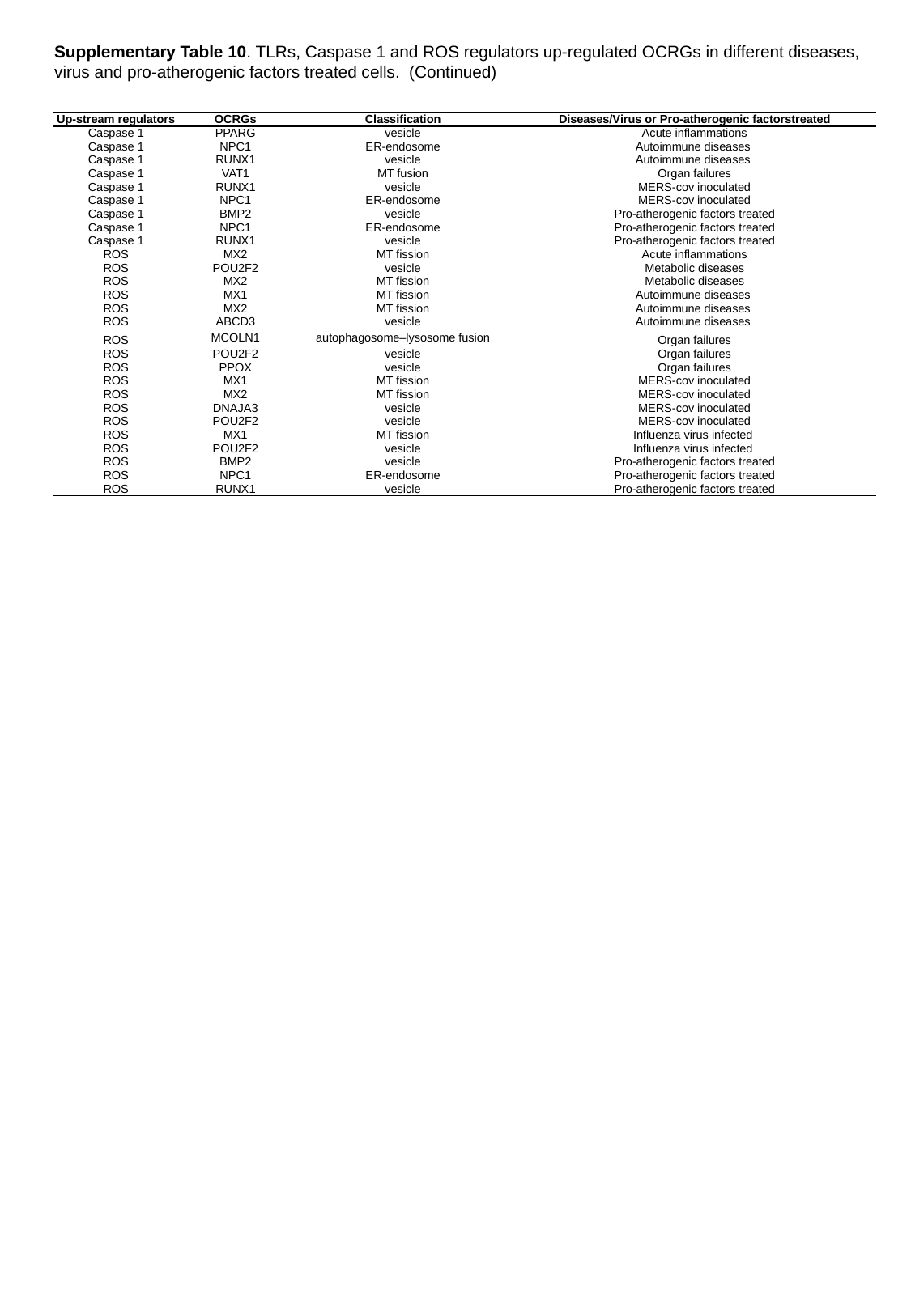

Supplementary Table 10. TLRs, Caspase 1 and ROS regulators up-regulated OCRGs in different diseases, virus and pro-atherogenic factors treated cells. (Continued)
| Up-stream regulators | OCRGs | Classification | Diseases/Virus or Pro-atherogenic factorstreated |
| --- | --- | --- | --- |
| Caspase 1 | PPARG | vesicle | Acute inflammations |
| Caspase 1 | NPC1 | ER-endosome | Autoimmune diseases |
| Caspase 1 | RUNX1 | vesicle | Autoimmune diseases |
| Caspase 1 | VAT1 | MT fusion | Organ failures |
| Caspase 1 | RUNX1 | vesicle | MERS-cov inoculated |
| Caspase 1 | NPC1 | ER-endosome | MERS-cov inoculated |
| Caspase 1 | BMP2 | vesicle | Pro-atherogenic factors treated |
| Caspase 1 | NPC1 | ER-endosome | Pro-atherogenic factors treated |
| Caspase 1 | RUNX1 | vesicle | Pro-atherogenic factors treated |
| ROS | MX2 | MT fission | Acute inflammations |
| ROS | POU2F2 | vesicle | Metabolic diseases |
| ROS | MX2 | MT fission | Metabolic diseases |
| ROS | MX1 | MT fission | Autoimmune diseases |
| ROS | MX2 | MT fission | Autoimmune diseases |
| ROS | ABCD3 | vesicle | Autoimmune diseases |
| ROS | MCOLN1 | autophagosome–lysosome fusion | Organ failures |
| ROS | POU2F2 | vesicle | Organ failures |
| ROS | PPOX | vesicle | Organ failures |
| ROS | MX1 | MT fission | MERS-cov inoculated |
| ROS | MX2 | MT fission | MERS-cov inoculated |
| ROS | DNAJA3 | vesicle | MERS-cov inoculated |
| ROS | POU2F2 | vesicle | MERS-cov inoculated |
| ROS | MX1 | MT fission | Influenza virus infected |
| ROS | POU2F2 | vesicle | Influenza virus infected |
| ROS | BMP2 | vesicle | Pro-atherogenic factors treated |
| ROS | NPC1 | ER-endosome | Pro-atherogenic factors treated |
| ROS | RUNX1 | vesicle | Pro-atherogenic factors treated |

## Slide 12
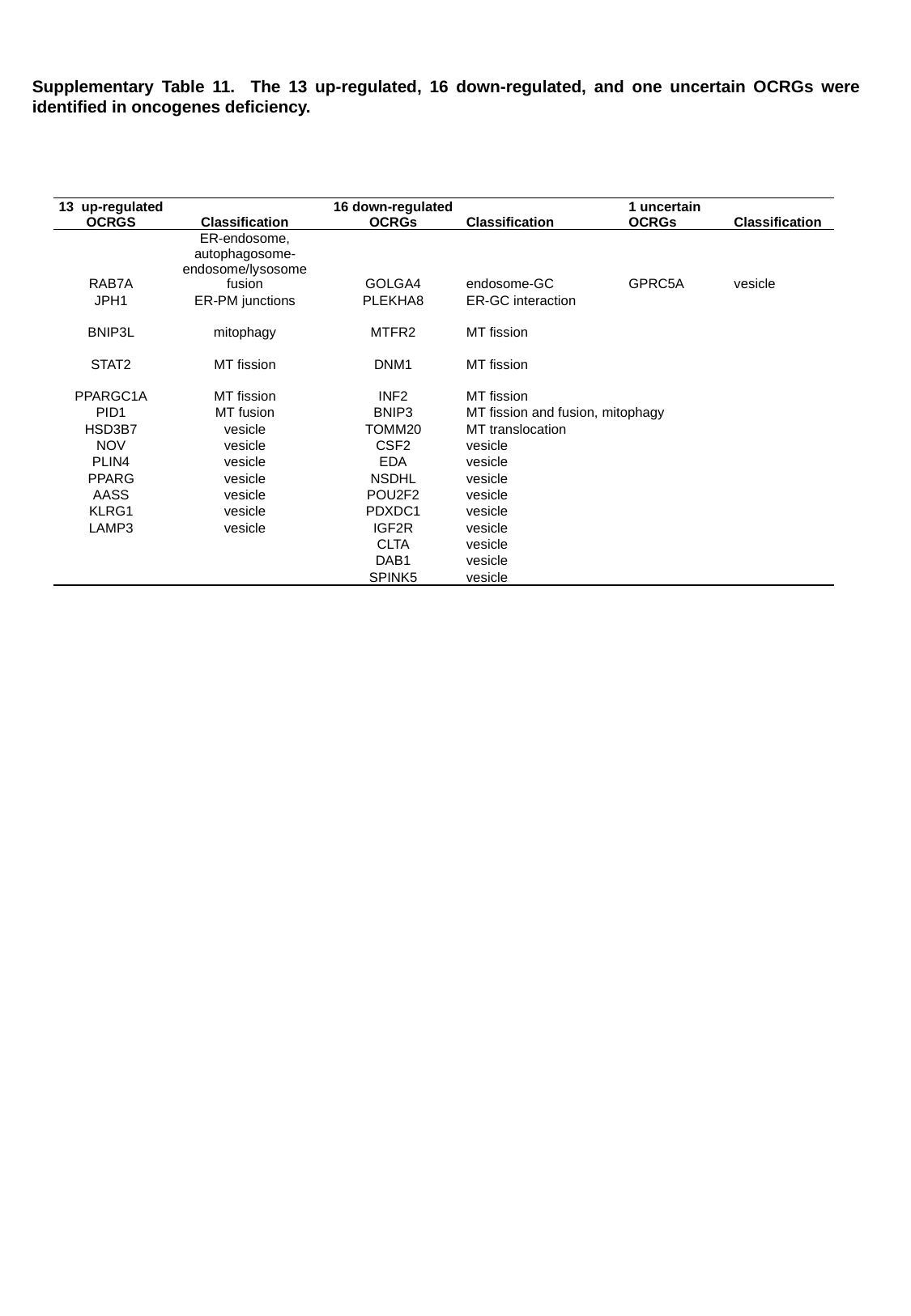

Supplementary Table 11. The 13 up-regulated, 16 down-regulated, and one uncertain OCRGs were identified in oncogenes deficiency.
| 13 up-regulated OCRGS | Classification | 16 down-regulated OCRGs | Classification | | 1 uncertain OCRGs | Classification |
| --- | --- | --- | --- | --- | --- | --- |
| RAB7A | ER-endosome, autophagosome-endosome/lysosome fusion | GOLGA4 | endosome-GC | | GPRC5A | vesicle |
| JPH1 | ER-PM junctions | PLEKHA8 | ER-GC interaction | | | |
| BNIP3L | mitophagy | MTFR2 | MT fission | | | |
| STAT2 | MT fission | DNM1 | MT fission | | | |
| PPARGC1A | MT fission | INF2 | MT fission | | | |
| PID1 | MT fusion | BNIP3 | MT fission and fusion, mitophagy | | | |
| HSD3B7 | vesicle | TOMM20 | MT translocation | | | |
| NOV | vesicle | CSF2 | vesicle | | | |
| PLIN4 | vesicle | EDA | vesicle | | | |
| PPARG | vesicle | NSDHL | vesicle | | | |
| AASS | vesicle | POU2F2 | vesicle | | | |
| KLRG1 | vesicle | PDXDC1 | vesicle | | | |
| LAMP3 | vesicle | IGF2R | vesicle | | | |
| | | CLTA | vesicle | | | |
| | | DAB1 | vesicle | | | |
| | | SPINK5 | vesicle | | | |

## Slide 13
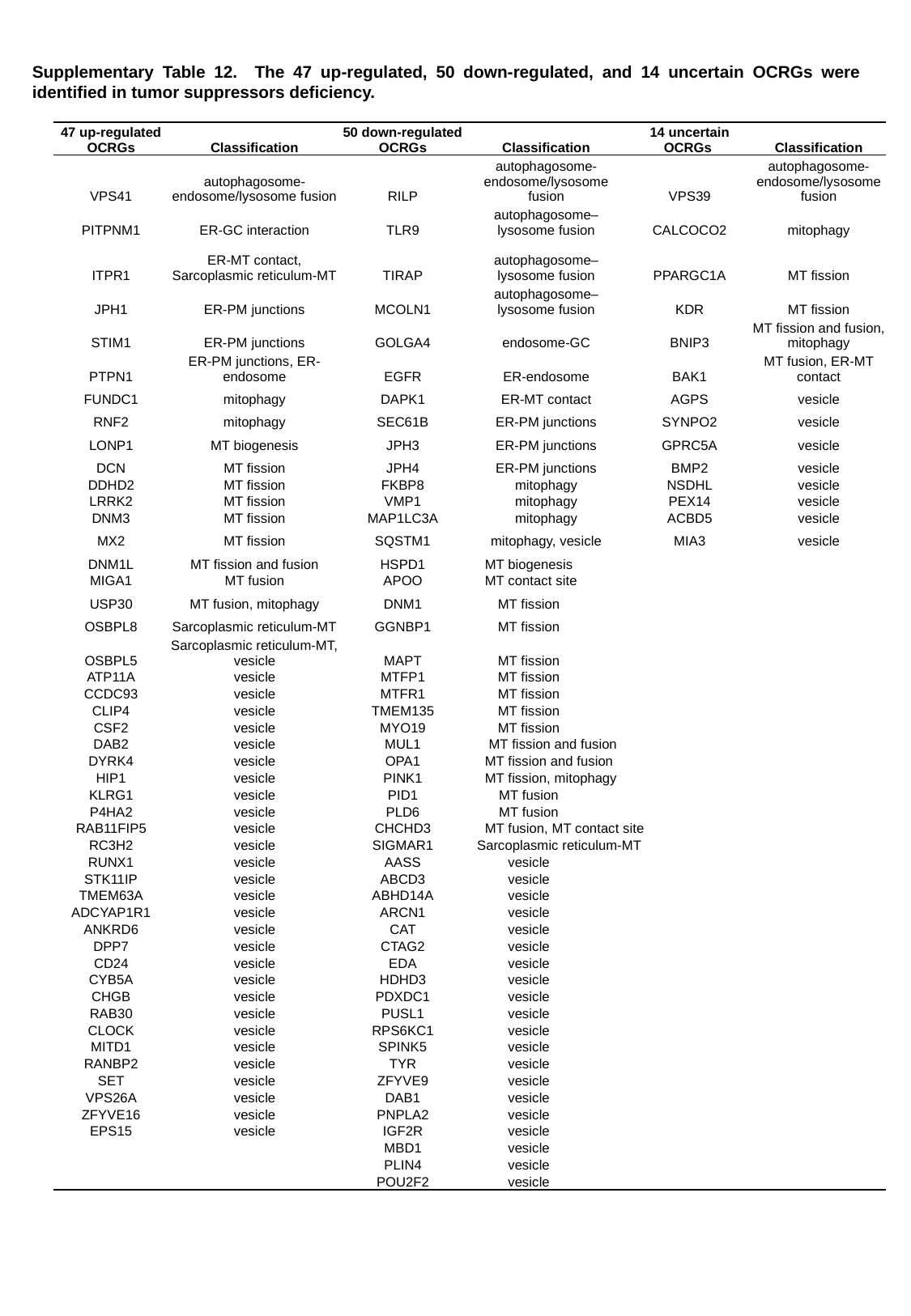

Supplementary Table 12. The 47 up-regulated, 50 down-regulated, and 14 uncertain OCRGs were identified in tumor suppressors deficiency.
| 47 up-regulated OCRGs | Classification | 50 down-regulated OCRGs | Classification | | 14 uncertain OCRGs | Classification |
| --- | --- | --- | --- | --- | --- | --- |
| VPS41 | autophagosome-endosome/lysosome fusion | RILP | autophagosome-endosome/lysosome fusion | | VPS39 | autophagosome-endosome/lysosome fusion |
| PITPNM1 | ER-GC interaction | TLR9 | autophagosome–lysosome fusion | | CALCOCO2 | mitophagy |
| ITPR1 | ER-MT contact, Sarcoplasmic reticulum-MT | TIRAP | autophagosome–lysosome fusion | | PPARGC1A | MT fission |
| JPH1 | ER-PM junctions | MCOLN1 | autophagosome–lysosome fusion | | KDR | MT fission |
| STIM1 | ER-PM junctions | GOLGA4 | endosome-GC | | BNIP3 | MT fission and fusion, mitophagy |
| PTPN1 | ER-PM junctions, ER-endosome | EGFR | ER-endosome | | BAK1 | MT fusion, ER-MT contact |
| FUNDC1 | mitophagy | DAPK1 | ER-MT contact | | AGPS | vesicle |
| RNF2 | mitophagy | SEC61B | ER-PM junctions | | SYNPO2 | vesicle |
| LONP1 | MT biogenesis | JPH3 | ER-PM junctions | | GPRC5A | vesicle |
| DCN | MT fission | JPH4 | ER-PM junctions | | BMP2 | vesicle |
| DDHD2 | MT fission | FKBP8 | mitophagy | | NSDHL | vesicle |
| LRRK2 | MT fission | VMP1 | mitophagy | | PEX14 | vesicle |
| DNM3 | MT fission | MAP1LC3A | mitophagy | | ACBD5 | vesicle |
| MX2 | MT fission | SQSTM1 | mitophagy, vesicle | | MIA3 | vesicle |
| DNM1L | MT fission and fusion | HSPD1 | MT biogenesis | | | |
| MIGA1 | MT fusion | APOO | MT contact site | | | |
| USP30 | MT fusion, mitophagy | DNM1 | MT fission | | | |
| OSBPL8 | Sarcoplasmic reticulum-MT | GGNBP1 | MT fission | | | |
| OSBPL5 | Sarcoplasmic reticulum-MT, vesicle | MAPT | MT fission | | | |
| ATP11A | vesicle | MTFP1 | MT fission | | | |
| CCDC93 | vesicle | MTFR1 | MT fission | | | |
| CLIP4 | vesicle | TMEM135 | MT fission | | | |
| CSF2 | vesicle | MYO19 | MT fission | | | |
| DAB2 | vesicle | MUL1 | MT fission and fusion | | | |
| DYRK4 | vesicle | OPA1 | MT fission and fusion | | | |
| HIP1 | vesicle | PINK1 | MT fission, mitophagy | | | |
| KLRG1 | vesicle | PID1 | MT fusion | | | |
| P4HA2 | vesicle | PLD6 | MT fusion | | | |
| RAB11FIP5 | vesicle | CHCHD3 | MT fusion, MT contact site | | | |
| RC3H2 | vesicle | SIGMAR1 | Sarcoplasmic reticulum-MT | | | |
| RUNX1 | vesicle | AASS | vesicle | | | |
| STK11IP | vesicle | ABCD3 | vesicle | | | |
| TMEM63A | vesicle | ABHD14A | vesicle | | | |
| ADCYAP1R1 | vesicle | ARCN1 | vesicle | | | |
| ANKRD6 | vesicle | CAT | vesicle | | | |
| DPP7 | vesicle | CTAG2 | vesicle | | | |
| CD24 | vesicle | EDA | vesicle | | | |
| CYB5A | vesicle | HDHD3 | vesicle | | | |
| CHGB | vesicle | PDXDC1 | vesicle | | | |
| RAB30 | vesicle | PUSL1 | vesicle | | | |
| CLOCK | vesicle | RPS6KC1 | vesicle | | | |
| MITD1 | vesicle | SPINK5 | vesicle | | | |
| RANBP2 | vesicle | TYR | vesicle | | | |
| SET | vesicle | ZFYVE9 | vesicle | | | |
| VPS26A | vesicle | DAB1 | vesicle | | | |
| ZFYVE16 | vesicle | PNPLA2 | vesicle | | | |
| EPS15 | vesicle | IGF2R | vesicle | | | |
| | | MBD1 | vesicle | | | |
| | | PLIN4 | vesicle | | | |
| | | POU2F2 | vesicle | | | |
