# Supplementary material for: Organelle Crosstalk Regulators Are Regulated in Diseases, Tumors, and Regulatory T Cells: Novel Classification of Organelle Crosstalk Regulators
Source: Front Cardiovasc Med. 2021 Jul 22;8:713170. doi: 10.3389/fcvm.2021.713170 (PMC8339352; doi:10.3389/fcvm.2021.713170)
Supplement: Supplementary file 20 [file Presentation_2.pptx]

## Slide 1
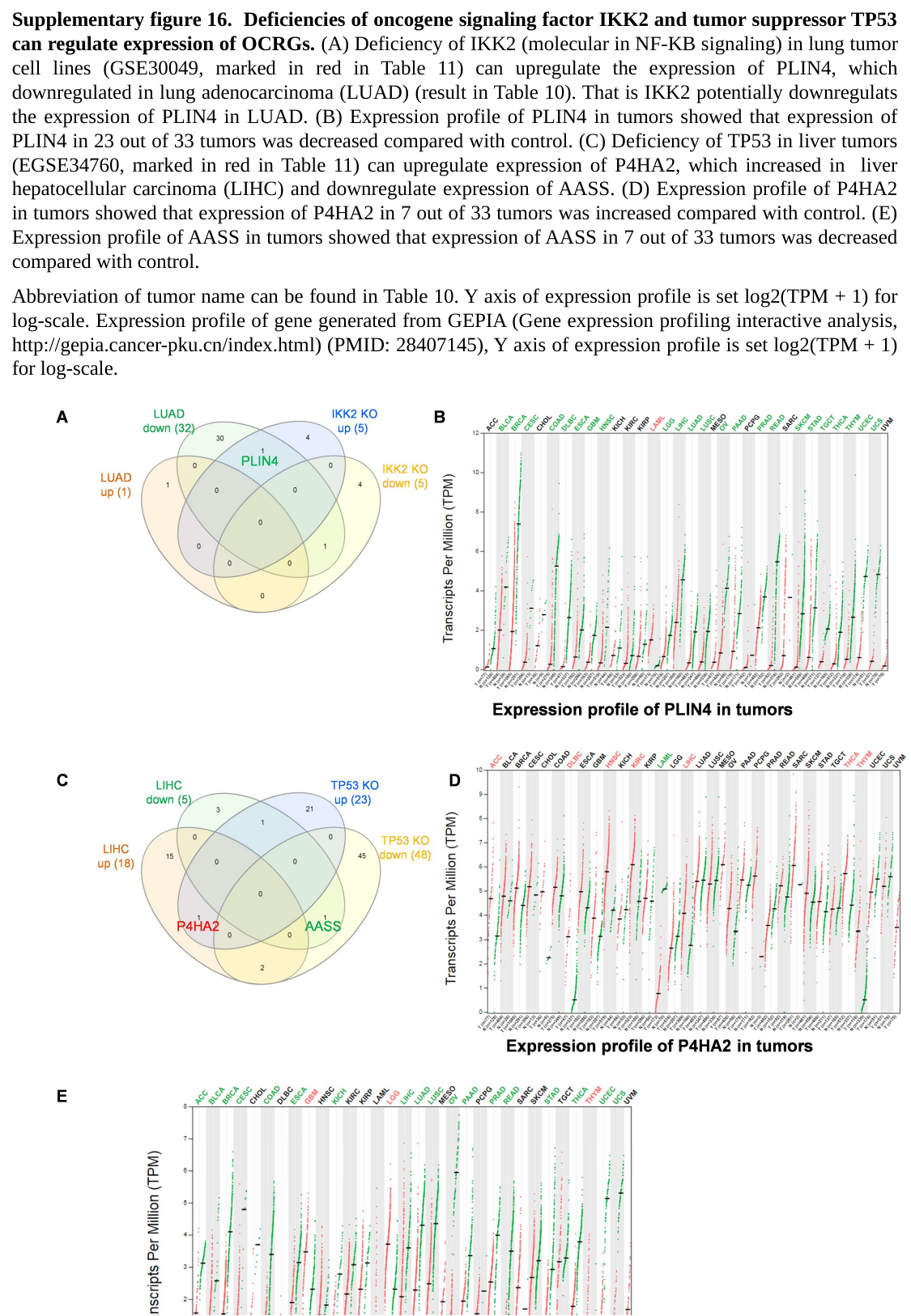

Supplementary figure 16. Deficiencies of oncogene signaling factor IKK2 and tumor suppressor TP53 can regulate expression of OCRGs. (A) Deficiency of IKK2 (molecular in NF-KB signaling) in lung tumor cell lines (GSE30049, marked in red in Table 11) can upregulate the expression of PLIN4, which downregulated in lung adenocarcinoma (LUAD) (result in Table 10). That is IKK2 potentially downregulats the expression of PLIN4 in LUAD. (B) Expression profile of PLIN4 in tumors showed that expression of PLIN4 in 23 out of 33 tumors was decreased compared with control. (C) Deficiency of TP53 in liver tumors (EGSE34760, marked in red in Table 11) can upregulate expression of P4HA2, which increased in liver hepatocellular carcinoma (LIHC) and downregulate expression of AASS. (D) Expression profile of P4HA2 in tumors showed that expression of P4HA2 in 7 out of 33 tumors was increased compared with control. (E) Expression profile of AASS in tumors showed that expression of AASS in 7 out of 33 tumors was decreased compared with control.
Abbreviation of tumor name can be found in Table 10. Y axis of expression profile is set log2(TPM + 1) for log-scale. Expression profile of gene generated from GEPIA (Gene expression profiling interactive analysis, http://gepia.cancer-pku.cn/index.html) (PMID: 28407145), Y axis of expression profile is set log2(TPM + 1) for log-scale.
