# Supplementary material for: Organelle Crosstalk Regulators Are Regulated in Diseases, Tumors, and Regulatory T Cells: Novel Classification of Organelle Crosstalk Regulators
Source: Front Cardiovasc Med. 2021 Jul 22;8:713170. doi: 10.3389/fcvm.2021.713170 (PMC8339352; doi:10.3389/fcvm.2021.713170)
Supplement: Supplementary file 21 [file Data_Sheet_1.docx]

**Supplementary figure 1. Donut chart was used to classify 260 OCRGs.** (A) In the total genes, vesicle-related genes account for 44%, followed by mitophagy genes (10%), MT fission genes (10%), ER-MT contact genes (5%), MT fusion genes (5%), ER-PM junctions (4%), Sarcoplasmic reticulum-MT, autophagosome-endosome/lysosome fusion, MT fission and fusion, MT translocation, and ER-endosome contact genes account for 3%, respectively. MT biogenesis genes account for 2%. MT contact site, ER-GC interaction, autophagosome–lysosome fusion and endosome-GC related genes account for 1%, respectively. (B) PINK1, BAK1, CHCHD3, MFN1, MFN2, OMA1, USP30, BNIP3, FIS1, SQSTM1, TBK1, VDAC1, RMDN3, VAPB, VAPA, ITPR1, HSPA9, OSBPL5, PTPN1 and RAB7A those 20 genes have more than one classification/function.

Note: The 20 genes have more than one classification/function (repetitive genes) which will be counted more than one time.

**Supplementary figure 2. 260 OCRGs were confirmed to enrich in mitochondrion organization, mitochondrial fission, autophagy, vesicle organization and organelle organization.** Enriched Ontology Clusters were generated by using Metascape software (http://metascape.org/gp/index.html#/main/step1; PMID: 30944313). (A) Heat map of ontology clusters across our selected 260 OCRGs. GO:0007005 mitochondrion organization, GO:0006914 autophagy, GO:0008053 mitochondrial fusion, GO:0016050 vesicle organization and R-HSA-9609507 Protein localization were the top5 significant Gene Ontology or signaling that organelle interactionss and vesicle-related genes enriched. (B) The enrichment network of ontology clusters, colored by GO enrichment (left). One term from each cluster is selected to have its term description shown as label. More specifically, each term is represented by a circle node, where its size is proportional to the number of input genes fall into that term, and nodes of the same color belong to the same cluster; The enrichment network of ontology clusters colored by p-Value (right). The dark the color, the more statistically significant the node is. (C) Protein-protein interaction network showed the interaction among OCRGs. Molecular Complex Detection (MCODE, https://omictools.com/mcode-tool) algorithm was applied to this network to identify neighborhoods where proteins are densely connected. Seven MCODE networks were identified and each MCODE network is assigned a unique color.

**Supplementary figure 3. Enrichment analysis results of upregulated OCRGs and downregulated OCRGs in influenza virus- and icMERS-inoculated cells.** (A) Enrichment pathways of 40 upregulated OCRGs in influenza virus-infected cells. (B) Enrichment pathways of 143 downregulated OCRGs in influenza virus-infected cells. (C) Enrichment pathways of 59 upregulated OCRGs in icMERS-inoculated cells. (D) Enrichment pathways of 62 downregulated OCRGs in icMERS-inoculated cells.

**Supplementary figure 4. Venn diagram showed one group and four pathways were downregulated in influenza virus- and icMERS-inoculated cells.** (A) Autophagosome–lysosome fusion group is exclusively downregulated in both influenza virus- and icMERS-inoculated cells. (B) GO:0006839:mitochondrial transport, ko04144:Endocytosis, GO:0045055:regulated exocytosis and GO:0097352:autophagosome maturation were the common downregulated pathways in influenza virus- and icMERS-inoculated cells.

**Supplementary figure 5. organelle interactions and vesicle-related genes were significantly expressed in LPS treated human lung microvascular endothelial cells.** (A) In LPS treated human lung microvascular endothelial cells in different times (4, 8 and 24 hours), CD24, CSF2, POU2F2 were the common upregulated genes, PLEKHA8 and RAB11FIP1 were the common down-regulated genes. Removing the overlapped 4 genes, 20 genes are upregulated and 26 genes are down-regulated in LPS treated endothelial cells. (B) In the 20 up-regulated genes, vesicle (60%) and MT fission (25%) genes were accounted higher proportion, followed by mitophagy (5%), ER-MT contact (5%) and ER-PM junctions related genes (5%); In the 26 downregulated genes, number of vesicle gens are highest (62%) followed by ER-PM junctions related genes (11%) and MT fission genes (11%). (C) Enrichment analysis results of 20 upregulated genes showed Cytokine Signaling in Immune system (red line marked) is upregulated and this change suggests LPS maybe induce inflammation via organelle interactions and vesicle-related genes. (D) Enrichment analysis results of 26 downregulated genes showed regulation of calcium ion transmembrane transporter activity signaling (red line marked) is downregulated and this change suggest LPS can change calcium ion transmembrane transporter activity via organelle interactions and vesicle-related genes in endothelial cells.

**Supplementary figure 6. Venn diagram of the up- and down-regulated OCRGs in deficiencies of Tlr2, Tlr4 and Tlr3/7/9.** (A) The results of significant expression changed genes in deficiencies of Tlr2 were combined (GSE24935, GSE56426 and GSE45861), as a result, a total of 29 genes were upregulated and 57 genes were downregulated in deficiency of Tlr2. (B) The results of significant expression changed genes in deficiencies of Tlr4 after LPS treatment and Lipid A treatment were combined (GSE31066), as a result, a total of seven genes were upregulated and 12 genes were downregulated in deficiency of Tlr4. (C) The results of significant expression changed genes in deficiencies of Tlr3/7/9 after injection with MOPC cells in day 4 and day 6 were combined (GSE92358), as a result, a total of nine genes were upregulated and 12 genes were downregulated in deficiencies of Tlr3/7/9.

**Supplementary figure 7. TLR2 can regulate the expression of OCRGs in diseases, virus and inflammatory factors treated cells.** According to the model we proposed in Figure 8A, the upregulated genes in diseases and downregulated genes in deficiency of TLR2 (marked in red), and the downregulated genes in diseases and upregulated genes in deficiency of TLR2 (marked in green) were OCRGs potentially regulated by TLR2. (A) In acute inflammatory disease (AI), TLR2 potentially upregulated the expression of VMP1, CTSA, IGF2R and PLIN3, and downregulated the expression of CLIP4, PID1, SNX1, GPRC5A and EGFR. (B) In metabolic disease (MD), TLR2 potentially upregulated the expression of PLEKHA8, HSPD1, MTFP1, ACBD5, VMP1 and CTSA, and downregulated the expression of EGFR, VAPA, MBD1 and ATP11A. (C) In autoimmune diseases (AD), TLR2 potentially upregulated the expression of CSF2, DNM1, MX1, SEC61B, ABCD3,, NACC1, VMP1, CHCHD3, GOLGA1 and ACBD5, and downregulated the expression of DAPK1, HIP1, SNX1, DNM1L, BMP2, EDA, CLIP4, RAB11FIP5 and TNPO3. (D) In organ failure diseases (OF), TLR2 potentially down regulated the expression of PID1, EGFR, PLIN4, LRRK2, MFF, VAPA, ATP11A, CLIP4, SNX1, ADCYAP1R1, EDA, HIP1 and TMEM63A. (E) In icMERS treated cells, TLR2 potentially upregulated eight and downregulated six OCRGs. (F) In influenza virus treated cells, TLR2 potentially upregulated nine and downregulated 21 OCRGs. (G) In inflammatory factors treated cells, TLR2 potentially upregulated five and downregulated three OCRGs.

**Supplementary figure 8. TLR4 can regulate the expression of OCRGs in diseases, virus and inflammatory factors treated cells.** According to the model we proposed in Figure 8A, the upregulated genes in diseases and down-regulated genes in deficiency of TLR4 (marked in red), and the downregulated genes in diseases and upregulated genes in deficiency of TLR4 (marked in green) were OCRGs potentially regulated by TLR4. (A) In acute inflammatory disease (AI), TLR4 potentially up regulated the expression of MX2, PICALM and RAB20, and down regulated the expression of TOMM40. (B) In metabolic disease (MD), TLR4 potentially up regulated the expression of VAMP8, MX2, SQSTM1, AP1B1 and RAB20. (C) In autoimmune diseases (AD), TLR4 potentially up regulated the expression of RNF2, CSF2 and MX2, and down regulated the expression of TOMM40 and HIP1. (D) In organ failure diseases (OF), TLR4 potentially up regulated the expression of VAMP8, down regulated the expression of STARD3NL, HIP1 and PPARG. (E) In icMERS treated cells, TLR4 potentially up regulated five and down regulated two OCRGs. (F) In influenza virus treated cells, TLR4 potentially up regulated two and down regulated three OCRGs. (G) In inflammatory factors treated cells, TLR4 potentially up regulated three and down regulated two OCRGs.

**Supplementary figure 9. Mechanism1: TLR3/7/9 can regulate the expression of OCRGs in diseases, virus and inflammatory factors treated cells.** According to the model we proposed in Figure 8A, the upregulated genes in diseases and downregulated genes in deficiency of TLR3/7/9 (marked in red), and the downregulated genes in diseases and upregulated genes in deficiency of TLR3/7/9 (marked in green) were OCRGs potentially regulated by TLR3/7/9. (A) In acute inflammatory disease, TLR3/7/9 potentially down regulated the expression of DDHD2 and EGFR. (B) In metabolic disease, TLR3/7/9 potentially up regulated the expression of JPH2 and POU2F2, and down regulated the expression of DDHD2, EGFR and CDKL1. (C) In autoimmune diseases, TLR3/7/9 potentially up regulated the expression of SPINK5, and down regulated the expression of BMP2, EDA and DDHD2. (D) In organ failure diseases, TLR3/7/9 potentially up regulated the expression of MCOLN1, POU2F2 and SYNPO2, down regulated the expression of EGFR, DDHD2, ACBD5, POLG, EDA and NMRK2. (E) In icMERS treated cells, TLR3/7/9 potentially up regulated four and down regulated two OCRGs. (F) In influenza virus treated cells, TLR3/7/9 potentially up regulated and down regulated four OCRGs, respectively. (G) In inflammatory factors treated cells, TLR3/7/9 potentially up regulated three and down regulated one OCRGs

**Supplementary figure 10. Mechanism 2: ROS regulator Nrf2 can regulate the expression of OCRGs in diseases.** According to the model we proposed in Figure 8B, the upregulated genes in diseases and in deficiency of Nrf2 (marked in red), and the downregulated genes in diseases and in deficiency of Nrf2 (marked in green) were OCRGs potentially regulated by Nrf2. (A) In acute inflammatory disease, Nrf2 potentially regulated the expression of MX2, CLIP4 and GPRC5A. (B) In metabolic disease, Nrf2 potentially up regulated the expression of MX2 and POU2F2. (C) In autoimmune diseases, Nrf2 potentially regulated the expression of MX1, MX2, ABCD3, CLIP4, USP30 and ITCH. (D) In organ failure diseases, Nrf2 potentially up regulated the expression of MCOLN1, POU2F2 and PPOX, down regulated the expression of USP30, JPH1, ITCH, AP1G1, CLIP4 and ADCYAP1R1. (up-regulated genes marked in red and down-regulated genes marked in green)

**Supplementary figure 11. Caspase 1 can regulate the expression of OCRGs in diseases, virus and inflammatory factors treated cells.** According to the model we proposed in Figure 8A, the upregulated genes in diseases and downregulated genes in deficiency of Caspase 1 (marked in red), and the downregulated genes in diseases and upregulated genes in deficiency of Caspase 1 (marked in green) were OCRGs potentially regulated by Caspase 1.

**Supplementary figure 12. Enrichment analysis of tumors in different systems showed the upregulated OCRGs in most tumors can be enriched in mitochondrion organization pathways.**

**Supplementary figure 13. Mitochondrial protein translocation gene TOMM40 were upregulated in cancers.** (A) Venn diagram of the upregulated OCRGs showed TOMM40 were shared gene in five digestive system cancers (Data in Table 2 was used). (B) Expression of TOMM40 was significantly increased in 14 out of 33 types of cancers and was decreased in Acute Myeloid Leukemia (LAML). (C-E) PPI and enrichment were analyzed using STRING database (https://string-db.org/). Enrichment analysis results of the shared gene and top p10 connected proteins were downloaded and visualized by using Cytoscape software 3.7.2 (https://cytoscape.org/). The result showed that the most relative top10 genes to TOMM40 was the TOMMs and TIMMs, and mainly enriched in protein targeting to mitochondrion, protein transporter activity and mitochondrial protein complex in biology process, molecular function and cell component, respectively. The enriched pathway of these 11 genes is mitochondrial protein import, Pink/Parkin mediated mitophagy and Ub-specific processing proteases. (F) Overall Survival map of Hazardous Ratio (HR) of TOMM40 and the interacted 10 genes. The result showed overall survival of patients in most cancers were positively related to these 11 genes (red box). (G) Disease Free Survival (RFS) map of Hazardous Ratio (HR) of TOMM40 and the interacted 10 genes. The result showed RFS of patients in most cancers were positively related to these 11 genes (red box). The blue box was the negative correlation between the expression of genes and RFS. Abbreviation of tumor name can be found in Table 10. Expression profile of gene generated from GEPIA (Gene expression profiling interactive analysis, http://gepia.cancer-pku.cn/index.html) (PMID: 28407145), Y axis of expression profile is set log2(TPM + 1) for log-scale. Survival map is from GEPIA2 (http://gepia2.cancer-pku.cn/#index) (PMID: 31114875).

**Supplementary figure 14. Upregulated TOMM40 in digestive cancers were correlated with immune infiltrates.** (A) Relations between abundance of tumor-infiltrating lymphocytes (TILs) and expression of TOMM40 indicated that abundance of act CD8, act CD4, CD56 and monocyte cells were positively correlated with expression of TOMM40 in most of the cancers. Abundance of other TILs was negatively correlated with expression of TOMM40 in most of the cancers. (B) Relations between expression of TOMM40 and immuno-inhibitors indicated PVRL2 was positively correlated with TOMM40 in most of cancers. (C) Relations between expression of TOMM40 and immuno-stimulators indicated CD276, PVR, TNFRSF18, TNFRSF25 and TNFRSF4 were positively correlated with TOMM40 in most of cancers. (D) Relations between expression of TOMM40 and MHCs showed HLA-A, HLA-B, HLA-C, HLA-F, TAP1, TAP2 and TAPBP molecules were positively correlated with expression of TOMM40 in most of cancers. C-F were generated by TISIDB database (http://cis.hku.hk/TISIDB/index.php PMID: 30903160). Abbreviation of tumor name can be found in Table 10.

**Supplementary figure 15. Oncogenes and tumor suppressors regulated expression of OCRGs.** (A) Removing the overlapped five genes in all upregulated genes and in all downregulated genes, 13 genes are upregulated and 16 genes are downregulated exclusively in oncogenes deficiency. (B) Removing the overlapped 14 genes in all upregulated genes and in all downregulated genes, 47 genes are upregulated and 50 genes are downregulated exclusively in tumor suppressors deficiency. (C) Donut chart showed the ratio of classification of upregulated OCRGs (left) and downregulated OCRGs (right) in oncogenes and tumor suppressors deficiency. 13 upregulated OCRGs in oncogenes deficiency were classified seven types (vesicle 50%, MT fission 14%, Mitophagy 7%, MT fusion 7%, ER-PM junctions 7%, Autophagosome-endosome/lysosome fusion 7% and ER-endosome 7%). 47 upregulated OCRGs in tumor suppressors deficiency were classified 12 types (except for MT translocation, MT contact site, Autophagosome–lysosome fusion and Endosome-GC OCRGs) (left). 16 downregulated OCRGs in oncogenes deficiency were classified seven types (vesicle accounts for 53%, MT fission genes accounts for 18%, Mitophagy, MT fission and fusion, MT translocation, ER-GC interaction and Endosome-GC OCRGs accounts for 6%, respectively). 50 down-regulated OCRGs in tumor suppressors deficiency were classified 14 types.

**Supplementary figure 16. Deficiencies of oncogene signaling factor IKK2 and tumor suppressor TP53 can regulate expression of OCRGs.** (A) Deficiency of IKK2 (molecular in NF-KB signaling) in lung tumor cell lines (GSE30049, marked in red in Table 11) can upregulate the expression of PLIN4, which downregulated in lung adenocarcinoma (LUAD) (result in Table 10). That is IKK2 potentially downregulats the expression of PLIN4 in LUAD. (B) Expression profile of PLIN4 in tumors showed that expression of PLIN4 in 23 out of 33 tumors was decreased compared with control. (C) Deficiency of TP53 in liver tumors (EGSE34760, marked in red in Table 11) can upregulate expression of P4HA2, which increased in liver hepatocellular carcinoma (LIHC) and downregulate expression of AASS. (D) Expression profile of P4HA2 in tumors showed that expression of P4HA2 in 7 out of 33 tumors was increased compared with control. (E) Expression profile of AASS in tumors showed that expression of AASS in 7 out of 33 tumors was decreased compared with control.

Abbreviation of tumor name can be found in Table 10. Y axis of expression profile is set log2(TPM + 1) for log-scale. Expression profile of gene generated from GEPIA (Gene expression profiling interactive analysis, http://gepia.cancer-pku.cn/index.html) (PMID: 28407145), Y axis of expression profile is set log2(TPM + 1) for log-scale.
